# Whole-genome sequencing of European autochthonous and commercial pig breeds allows the detection of signatures of selection for adaptation of genetic resources to different breeding and production systems

**DOI:** 10.1186/s12711-020-00553-7

**Published:** 2020-06-26

**Authors:** Samuele Bovo, Anisa Ribani, Maria Muñoz, Estefania Alves, Jose P. Araujo, Riccardo Bozzi, Marjeta Čandek-Potokar, Rui Charneca, Federica Di Palma, Graham Etherington, Ana I. Fernandez, Fabián García, Juan García-Casco, Danijel Karolyi, Maurizio Gallo, Vladimir Margeta, José Manuel Martins, Marie J. Mercat, Giulia Moscatelli, Yolanda Núñez, Raquel Quintanilla, Čedomir Radović, Violeta Razmaite, Juliette Riquet, Radomir Savić, Giuseppina Schiavo, Graziano Usai, Valerio J. Utzeri, Christoph Zimmer, Cristina Ovilo, Luca Fontanesi

**Affiliations:** 1 Department of Agricultural and Food Sciences, Division of Animal Sciences, University of Bologna, Viale Fanin 46, 40127 Bologna, Italy; 2 Departamento Mejora Genética Animal, INIA, Crta. de la Coruña km. 7,5, 28040 Madrid, Spain; 3 Centro de Investigação de Montanha (CIMO), Instituto Politécnico de Viana do Castelo, Escola Superior Agrária, Refóios do Lima, 4990-706 Ponte de Lima, Portugal; 4 DAGRI - Animal Science Section, Università di Firenze, Via delle Cascine 5, 50144 Florence, Italy; 5 Kmetijski Inštitut Slovenije, Hacquetova 17, 1000 Ljubljana, Slovenia; 6 Instituto de Ciências Agrárias e Ambientais Mediterrânicas (ICAAM), Universidade de Évora, Polo da Mitra, Apartado 94, 7006-554 Évora, Portugal; 7 Earlham Institute, Norwich Research Park, Colney Lane, Norwich, NR47UZ UK; 8 Department of Animal Science, Faculty of Agriculture, University of Zagreb, Svetošimunska c. 25, 10000 Zagreb, Croatia; 9 Associazione Nazionale Allevatori Suini (ANAS), Via Nizza 53, 00198 Rome, Italy; 10 Faculty of Agrobiotechnical Sciences, University of Osijek, Vladimira Preloga 1, 31000 Osijek, Croatia; 11 IFIP Institut du porc, La Motte au Vicomte, BP 35104, 35651 Le Rheu Cedex, France; 12 Programa de Genética y Mejora Animal, IRTA, Torre Marimon, 08140 Caldes de Montbui, Barcelona, Spain; 13 Department of Pig Breeding and Genetics, Institute for Animal Husbandry, Belgrade-Zemun, 11080 Serbia; 14 Animal Science Institute, Lithuanian University of Health Sciences, Baisogala, Lithuania; 15 GenPhySE, INRAE, Université de Toulouse, Chemin de Borde-Rouge 24, Auzeville Tolosane, 31326 Castanet Tolosan, France; 16 Faculty of Agriculture, University of Belgrade, Nemanjina 6, Belgrade-Zemun, 11080 Serbia; 17 AGRIS SARDEGNA, Loc. Bonassai, 07100 Sassari, Italy; 18 Bäuerliche Erzeugergemeinschaft Schwäbisch Hall, Schwäbisch Hall, Germany

## Abstract

**Background:**

Natural and artificial directional selection in cosmopolitan and autochthonous pig breeds and wild boars have shaped their genomes and resulted in a reservoir of animal genetic diversity. Signatures of selection are the result of these selection events that have contributed to the adaptation of breeds to different environments and production systems. In this study, we analysed the genome variability of 19 European autochthonous pig breeds (Alentejana, Bísara, Majorcan Black, Basque, Gascon, Apulo-Calabrese, Casertana, Cinta Senese, Mora Romagnola, Nero Siciliano, Sarda, Krškopolje pig, Black Slavonian, Turopolje, Moravka, Swallow-Bellied Mangalitsa, Schwäbisch-Hällisches Schwein, Lithuanian indigenous wattle and Lithuanian White old type) from nine countries, three European commercial breeds (Italian Large White, Italian Landrace and Italian Duroc), and European wild boars, by mining whole-genome sequencing data obtained by using a DNA-pool sequencing approach. Signatures of selection were identified by using a single-breed approach with two statistics [within-breed pooled heterozygosity (H_P_) and fixation index (F_ST_)] and group-based F_ST_ approaches, which compare groups of breeds defined according to external traits and use/specialization/type.

**Results:**

We detected more than 22 million single nucleotide polymorphisms (SNPs) across the 23 compared populations and identified 359 chromosome regions showing signatures of selection. These regions harbour genes that are already known or new genes that are under selection and relevant for the domestication process in this species, and that affect several morphological and physiological traits (e.g. coat colours and patterns, body size, number of vertebrae and teats, ear size and conformation, reproductive traits, growth and fat deposition traits). Wild boar related signatures of selection were detected across all the genome of several autochthonous breeds, which suggests that crossbreeding (accidental or deliberate) occurred with wild boars.

**Conclusions:**

Our findings provide a catalogue of genetic variants of many European pig populations and identify genome regions that can explain, at least in part, the phenotypic diversity of these genetic resources.

## Background

Natural and artificial directional selection have shaped livestock genomes and led to many breeds and populations, which are considered the main reservoir of genetic diversity in farmed animals [1–3]. Under positive selection pressure, the frequency of favourable alleles increases rapidly in a population and generates a high level of population differentiation, with impacts on haplotype structures and extended linkage disequilibrium between the mutated sites and neighbouring loci [4]. Signatures of selection that remain in the livestock genomes are the result of combined human-driven and natural selection events, which have contributed to the adaptation of genetic resources to different environments and production systems.

Livestock genomes can be analysed by applying different genomic and statistical measures and approaches [e.g. Wright’s fixation index (F_ST_) within or between populations and pooled heterozygosity (H_P_), among other statistics] to reveal regions under selection. These regions can provide insights into the biological mechanisms that explain domestication and lead to morphological differentiation, specialized production performances and, in some cases, disease resistance and resilience (e.g. [5–10]).

Since the first domestication events, pig has been subject to artificial directional selection that has profoundly differentiated domestic genetic pools from the original European and Asian wild boar populations [11–14]. Although today the pig industry uses a few cosmopolitan highly selected breeds and lines in intensive production systems, a large number of local breeds still exist in many regions around the world. These autochthonous genetic resources are less performing than commercial populations and, mainly in Europe, they are associated with local and traditional niche markets [15]. Other common characteristics of these breeds are good adaptation to their local agro-climatic and environmental conditions, high rusticity, slower growth rate, high adipogenic potential and, for some of them, superior meat quality traits. They are usually raised under extensive or semi-extensive production systems and in marginal areas [15]. They are also characterized by a variety of coat colour phenotypes and specific morphological traits, which can have interesting scientific values [11, 16, 17]. In order to design sustainable conservation programs for these genetic resources, most of which are still unexplored, the first step is to characterize genetically their populations [18].

To date, a few whole-genome investigations have been performed, which are mainly based on single nucleotide polymorphism (SNP) arrays and include only a few European local pig breeds [19–21]. Most of these studies are based on small numbers of animals and provide preliminary information on their population structure. In the case of the Iberian and Casertana breeds, quantitative trait loci (QTL) and genome scans have identified genomic regions and mutations associated with morphological, production, meat and carcass traits [9, 10, 16, 17, 22–24]. Other studies have compared SNP datasets from Italian autochthonous and commercial breeds to identify population-informative markers and analyse their inbreeding levels [25, 26]. Muñoz et al. [27] and Ribani et al. [28] analysed candidate markers in major genes in 20 European local pig breeds and wild boar populations, which provided information on the segregation of relevant polymorphisms for breeding or traceability purposes [29]. A follow-up study on these breeds included the analysis of linkage disequilibrium, F_ST_ and effective population size using a medium-density SNP array [30].

Other studies that involved a few Asian and European pig breeds investigated signatures of selection in the porcine genome using SNP chip or partial/reduced or whole-genome re-sequencing datasets and highlighted loci of economic importance [7, 31–42].

Thus, it is important to take further the analyses of the genome of autochthonous and cosmopolitan pig genetic resources, including unexplored and poorly investigated breeds, which were developed under different human-driven evolutionary conditions (i.e. selection programs) and production systems.

In this study, we analysed the genome of 19 European autochthonous pig breeds from nine countries, three commercial Italian breeds and wild boars to identify signatures of selection by mining whole-genome sequencing data obtained by using a DNA-pool sequencing approach. SNPs were called and allele frequencies were estimated. Signatures of selection were identified by computing both within-breed H_P_ and F_ST_ statistics and comparing different groups according to domestication/selection levels (i.e. autochthonous vs. commercial vs. wild boars) and morphological (coat colours and patterns and body size) criteria. These breeds, some of them still unexplored, were from various production systems and breeding programmes in Europe. The results should help elucidate the adaptation of pig genetic resources of the European continent to natural and artificial selection pressures.

## Methods

### Animals

**Fig. 1:**
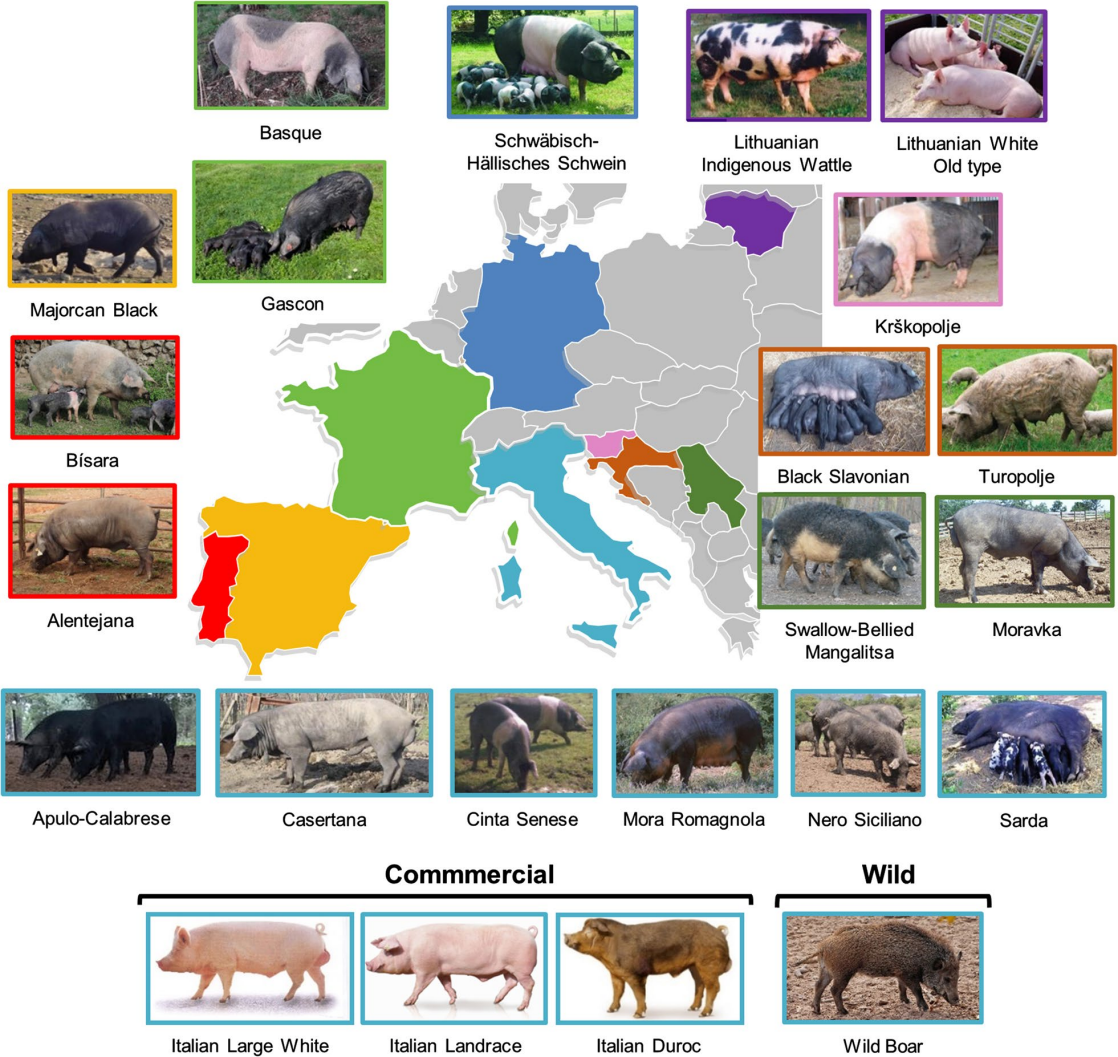
Phenotype and geographical origin of the 22 analysed pig breeds and wild boar populations

Blood samples were collected from 30 to 35 animals from each of the 22 pig breeds included in the study and distributed across nine European countries (from West to East and then North; Fig. 1): two from Portugal (Alentejana and Bísara); one from Spain (Majorcan Black); two from France (Basque and Gascon); six autochthonous (Apulo-Calabrese, Casertana, Cinta Senese, Mora Romagnola, Nero Siciliano and Sarda), and three commercial breeds (Italian Large White, Italian Landrace and Italian Duroc) from Italy; one from Slovenia (Krškopolje pig, thereafter referred to as Krškopolje); two from Croatia (Black Slavonian and Turopolje); two from Serbia (Moravka and Swallow-Bellied Mangalitsa); one from Germany (Schwäbisch-Hällisches Schwein); two from Lithuania (Lithuanian indigenous wattle and Lithuanian White old type). Selection of the individuals for sampling was performed such that highly related animals were avoided (no full- or half-sibs), when possible by balancing between sexes and prioritizing adult individuals or, at least, animals with the morphology of an adult. All sampled animals were registered in their respective Herd Books and had standard breed characteristics. In addition, 35 tissue samples from Italian wild boars, which had previously been tested for the absence of introgressed domestic alleles [28], were used in this study. In Additional file 1: Table S1, more details on the analysed animals and investigated breeds, including geographical distribution and phenotypic description, are provided [15, 28].

### DNA samples and sequencing

Genomic DNA was extracted from 8 to 15 mL of peripheral blood for each pig [collected in Vacutainer tubes containing 10% 0.5 M EDTA (ethylenediaminetetraacetic acid, disodium dihydrate salt) at pH 8.0] or from muscle tissues (for wild boars, provided by forest policemen; [43]). DNA extraction was performed using either a standardized phenol–chloroform protocol [44] or the NucleoSpin^®^Tissue commercial kit (Macherey–Nagel, Düren, Germany). Twenty-three DNA pools were obtained by pooling 30 (or 35) individual DNA samples at equimolar concentrations for each pool (see Additional file 1: Table S1).

The wild boars included in the “wild boar” pool were first genotyped for *MC1R* and *NR6A1* mutations [28] and only the animals that were homozygous for the wild type alleles were used. For each of the 22 DNA pools representing domestic pig breeds, a sequencing library was generated using the Truseq Nano DNA HT Sample preparation Kit (Illumina, USA) following the manufacturer’s recommendations. Then, DNA was sheared randomly to obtain 350-bp fragments, which were end-polished, A-tailed, and ligated with the full-length adapter for Illumina sequencing and subjected to PCR amplification. PCR products were purified (AMPure XP system) and libraries were analysed for size distribution by Agilent2100 Bioanalyzer and quantified using real-time PCR. The qualified libraries were fed into an Illumina HiSeq X Ten sequencer for paired-end sequencing, which resulted in 150-bp long reads. The wild boar DNA pool was sequenced from 250-bp fragment libraries, with 100-bp long paired-end reads, on the BGISeq 500 platform, following the provider’s procedures.

### Quality controls and sequence alignment

The obtained reads underwent several cleaning and filtering steps: removal of (i) adapters, (ii) reads containing more than 10% unknown bases (N), and (iii) reads containing low-quality bases (Q ≤ 5). FASTQ files were the inspected with the FASTQC v.0.11.7 software (https://www.bioinformatics.babraham.ac.uk/projects/fastqc/) that highlighted very high-quality reads. No other filtering procedures were carried out.

Reads were mapped to the latest version of the *Sus scrofa* reference genome (Sscrofa11.1) with the BWA tool 0.7.17 [45] using the MEM function and the parameters for paired-end data. Picard v.2.1.1 (https://broadinstitute.github.io/picard/) was used to remove duplicated reads. Whole-genome sequencing data statistics are in Additional file 1: Table S2.

### Detection of variants from sequencing data

The detection of SNPs was carried out with the CRISP v.122713 software [46]. CRISP parameters were tuned to maximize the discovery of variants (-ctpval − 0.6 -minc 1 -EM 0). A three-step filtering procedure was adopted to retain high-quality variants:

- Step (1): (i) retention of bi-allelic variants only, (ii) with a minimum read depth (RD_min_) in each pool equal to 10, (iii) a minimum number of alternative reads, across the DNA pools, equal to 3, (iv) a maximum read depth (Rd_max_), in each pool, equal to 68 (computed as proposed by Li [47]; RD_max_ = RD_mean_ + 4√RD_mean_, where RD_mean_ = 42), and (v) removal of variants that mapped to low-quality regions or suffered from strand-bias.
- Step (2): implementation of the quality filter procedures described in [48]. In spite of the low rate of false positives with CRISP [46], these procedures allow to filter out other possible false variants. In this step, we used the dbSNP v.150 database ([49]; accessed on February 27 2018; number of variants = 64,535,988). Briefly, variants were initially annotated as reported in dbSNP (“in.dbSNP” class) or not (“novel” class). Then, these two classes were subdivided in “rare” and “common” variants. Rare variants were defined as variants that present a minor allele frequency (MAF) lower than 0.0143. This value represents the “ideal” lower limit of detection (i.e. 1/70), since, in general, pools were composed of 35 diploid individuals (see Additional file 1: Table S1). This is an approximated estimation that did not take the average sequencing depth into account. Then, for the “rare” class of variants, we used the Kolmogorov–Smirnov (KS) test to compare the distributions of the quality score of the variants in the “in.dbSNP” and “novel” sub-classes. The KS test measures the similarity of the two distributions in a quantitative way via the *D*-statistics (a metric ranging from 0 to 1). Lower values of *D* indicate more similar distributions. Different cut-off values ranging from 0 to 50 with steps of 1, were tested. The CRISP quality score (Q_CRISP_) that minimizes the *D* value was selected as the best score (Q_CRISP_ = 21; (see Additional file 2: Figure S1).
- Step (3): to evaluate globally the quality of our dataset, we used the transition-to-transversion ratio (Ts/Tv) as quality indicator [50].

Variants on the mitochondrial genome (that can also be confused with differences in nuclear DNA sequences of mitochondrial origin or NUMTS; [51]) and variants on the sex chromosomes were discarded. Variant detection in the wild boar DNA pool was carried out with Samtools v.1.7 [52] by retaining the variants that were detected in the 22 pig DNA pools and considering an RD_min_ of 3. Variants were annotated using the Variant Effect Predictor (VEP) v.95.0 [53], by predicting their impact on the protein function with SIFT v.5.2.2 [54]. Statistics about detected and annotated variants are Additional file 1: Tables S3 and S4, respectively. Pipelines were developed either in Python v.2.7.12 or in R v.3.4.4 ([55]; the Kolmogorov–Smirnov test was carried out with the function “*ks.test*”).

SNP allele frequencies (AF) were estimated by counting the number of reads that include the SNP position. To evaluate the reliability of the estimation of AF obtained by DNA-pool sequencing, we used SNP chip data from Muñoz et al. [30], since the pigs of the autochthonous breeds included in the DNA pools were also genotyped with the GeneSeek^®^ GGP Porcine HD Genomic Profiler v1 (Illumina Inc, USA) panel (including 68,516 SNPs). Pearson’s correlation coefficient (*r*) was computed between sequencing and chip genotyping derived frequencies, excluding transversions GC ↔ CG i.e. 167) and AT ↔ TA (i.e. 147) and also SNPs that presented more than 10% of missing genotypes within each breed [56].

### Genetic diversity analyses

The genetic distance between pairs of populations was estimated by computing F_ST_ values for each SNP as described by Karlsson et al. [57]. In total, $$253 = \left( {\begin{array}{*{20}c} {23} \\ 2 \\ \end{array} } \right)$$ comparisons were carried out. Then, for each comparison, F_ST_ values were averaged over the number of SNPs analysed. We obtained an F_ST_ matrix of size 23 × 23 that was graphically represented via a heatmap and used to build a Neighbour-Joining (NJ) tree. Genetic distances between local pig breeds were compared with geographical distances via the Mantel test.

Pipelines were developed either in Python or in R (“corrplot”, “nj” and “mantel” functions of the “corrplot”, “ape” [58] and “vegan” libraries, respectively).

### Detection of signatures of selection

Pooled heterozygosity and fixation index statistics were used to identify potential signatures of selection in the analysed populations. Signatures of selection were computed in 100-kb sliding genome windows, with a step size of 100 kb. In total, 23,666 genome windows were computed. Similarly to the procedure described by Rubin et al. [6], each 100-kb window was selected after testing windows of variable sizes (from 50 to 300 kb) for the number of windows with less than 10 SNPs: window counts decreased asymptotically after this threshold (see Additional file 1: Table S5 and Additional file 2: Figure S2). Windows included in the analyses contained on average 1148 ± 551 SNPs. Windows with less than 20 SNPs and for which either the F_ST_ or the H_P_ index was mathematically impossible to compute were discarded (i.e. 399).

The H_P_ index was computed for each window by using the formula described by Rubin et al. [6, 7] and log2 transformed as proposed by Sun et al. [59]. The final H_P_ value, related to each breed, was estimated as the overall mean of H_P_ values.

For each window, the F_ST_ index was estimated according to the formula introduced by Karlsson et al. [57]. F_ST_ was calculated for each breed or for groups of breeds in different comparisons.

For a given breed, the F_ST_ value of each genome window was computed as the average across the *N−* 1 comparisons (*N* = 22; wild boar data were analysed separately) and then the final F_ST_ value, related to the breed, was estimated as the overall mean of F_ST_ values [60]. Analysis of the wild boar population followed the same approach by considering *N* = 23.

F_ST_ was calculated for the following groups of breeds and comparisons: (i) comparisons based on coat colour phenotypes, by grouping together red, white, black, belted and spotted breeds, (ii) comparisons based on the body size of the breeds (small, medium and large), (iii) comparison between autochthonous and commercial breeds, and (iv) comparison between wild boars and domestic breeds. The classification of the breeds was based on the morphological descriptions reported in [15]. Detailed information on the different groups of breeds/populations and comparisons is summarized in Additional file 1: Table S6.

As defined by Rubin et al. [6], putative signatures of selection were identified from genome windows at the extreme lower end of the distributions (see Additional file 1: Tables S7, S8). We considered, as outliers, the genome windows presenting either a H_P_ or an F_ST_ value above the 99.95th percentile of the related distribution. This led to the identification of 12 genome windows for each pool.

Data were graphically represented via Manhattan plots. Pipelines were developed either in Python or in R (“manhattan” function of the “qqman” library [61]). Genomic windows were computed with Bedtools v.2.17.0 [62].

### Annotation of genome windows

Each 100-kb window that displayed signatures of selection was annotated with the Bedtool v.2.17.0 program by retrieving annotated protein coding genes from the Sscrofa11.1 NCBI’s GFF file. Moreover, we extended the annotation by including the genes that were located in the ± 200-kb flanking regions of each window. Allele frequencies of SNPs within each extended genome window were plotted in R using the libraries “gplots” (function: heatmap.2) and “Sushi” [63].

The functional relevance of the genes annotated in regions of signatures of selection was evaluated based on a detailed analysis of the scientific literature and Gene Cards information [64]. Moreover, gene enrichment analysis over sets of human traits was carried out with Enrichr [65] via Fisher’s exact test. Analyses run over the GWAS catalogue 2019 [66], a curated collection of relationships between human phenotypes and genes, which annotate 19,378 genes in 1737 phenotypic gene sets. We ran breed-specific over-representation analyses by using as input into Enrichr the set of genes that were mapped within the genome windows concurrently identified via the H_P_ and F_ST_ analyses. We considered as statistically enriched terms those presenting: (i) at least four genes of the input set related to two or more genome windows and (ii) an adjusted *p*-value lower than 0.05.

Putative deleterious SNPs were identified based on their impact on the protein function, considering as harmful stop gain (SG) mutations, stop lost (SL) mutations and non-synonymous SNPs (nsSNPs) predicted as deleterious by SIFT. Moreover, allele frequency values were evaluated with respect to the wild boar population and the overlap with regions under selection was evaluated.

## Results

### Overview of sequencing data and detected variants

Sequencing of the 22 domestic pig DNA pools produced ~ 18.4 billion of reads, with an average number of sequenced read pairs per pool equal to ~ 418.9 million. Non-duplicated reads covered on average 98.45% of the *S. scrofa* genome with a mean mapped read depth (RD_mean_) of ~ 42×. The wild boar DNA pool genome accounted for ~ 164.2 million of reads, 96.6% of which were non-duplicated and covered 98.2% of the genome with an RD_mean_ of ~ 12×. Summary statistics of the sequencing data are in Additional file 1: Table S2.

A bioinformatic pipeline based on the CRISP tool [46] and the Kolmogorov–Smirnov statistical test [48] was implemented to detect high-quality variants. We detected 36,085,090 variants of which 5,018,696 were removed in the first step and 151,105 were removed in the second step. In total, 26,732,468 high-quality SNPs (autosomes and scaffolds) were used in further analyses. Summary statistics for these variants are in Additional file 1: Table S3. A Ts/Tv ratio of 2.40 was found for the sequence dataset.

VEP annotation of the detected SNPs is summarized in Additional file 1: Table S4. In total, 44,784,029 SNP-annotation pairs (several annotations per SNPs were possible) were retrieved, covering 22,165 genes out of the 22,452 annotated genes on the Sscrofa11.1 genome version. As expected, the largest number of SNPs was found in introns (~ 62%) and in intergenic regions (~ 35%). SNPs that impacted the gene at the protein level (i.e. start/stop gained/lost and missense SNPs) accounted for only 0.34% of all point mutations.

Allele frequencies estimated from DNA pool sequencing and SNP chip genotyping presented an average Pearson’s correlation coefficient *r* = 0.95, ranging from 0.91 to 0.98 (see Additional file 1: Table S9).

### Genetic diversity and relationships among the populations analyzed

**Fig. 2:**
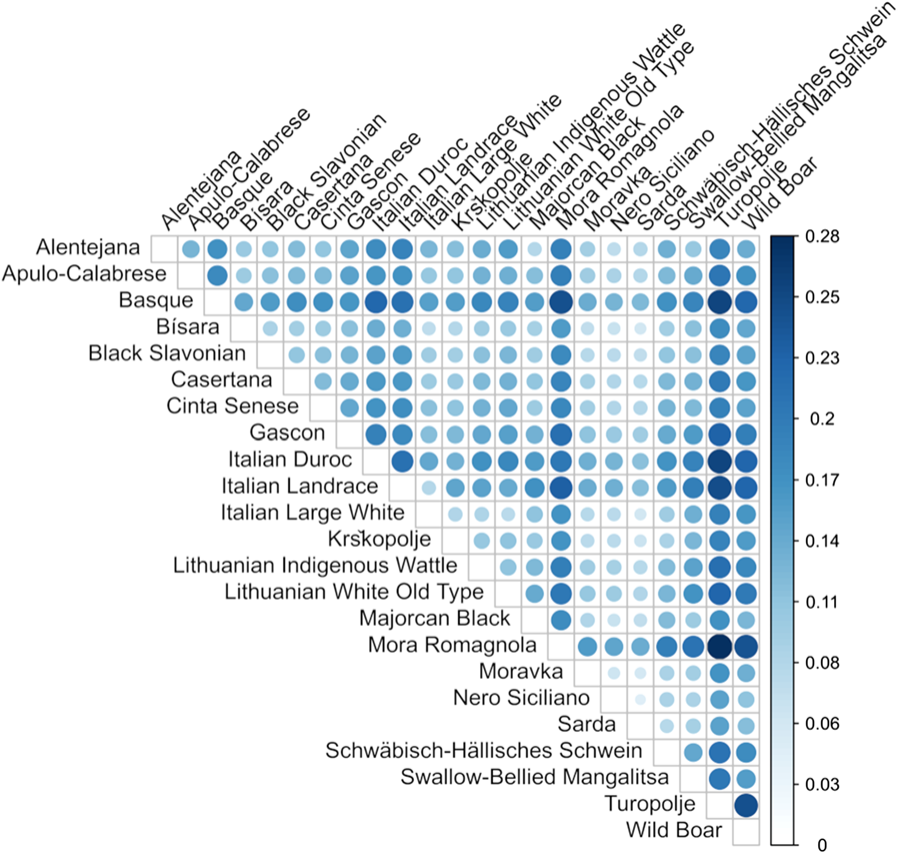
Heatmap plot of F_ST_ distances between breeds/populations

The genetic diversity between pairs of pig populations was investigated with the F_ST_ index (Fig. 2 and see Additional file 1: Table S10). The NJ tree built by using F_ST_ distances (see Additional file 2: Figure S3) depicted clusters that, generally, agreed with the geographical distribution of some of these breeds and their relationships derived from possible introgression and admixture events: (i) the two French breeds (Gascon and Basque) are clustered together; (ii) the two Central-Southern Italian breeds (Casertana and Apulo-Calabrese) are on the same branch; (iii) the two Lithuanian breeds (Lithuanian indigenous wattle and Lithuanian White old type) are clustered together with the Italian Large White and Italian Landrace breeds; Mora Romagnola and Italian Duroc are on the same branch; Krškopolje and Schwäbisch-Hällisches Schwein (two related belted-patterned breeds) are in the same cluster; several other breeds (Alentejana, Majorcan Black, Swallow-Bellied Mangalitsa, Cinta Senese and Black Slavonian) are clustered together with the wild boars, as previously described by Muñoz et al. [30].

Spatial analysis via the Mantel test (see Additional file 2: Figure S4) did not reveal any significant correlation between genetic and geographic distances (*r *= 0.09, *p*-value = 0.3).

### Identification of signatures of selection in 22 domestic pig breeds and in wild boars

Two approaches were applied to capture signatures of selection in the investigated pig breeds and wild boar population: (i) pooled heterozygosity, which identifies signatures of selection by analysing the genetic properties segregating within each breed, and (ii) fixation index, which provides information that is summarized for each single breed/population compared against all other breeds and wild boars.

### Pooled heterozygosity

**Fig. 3:**
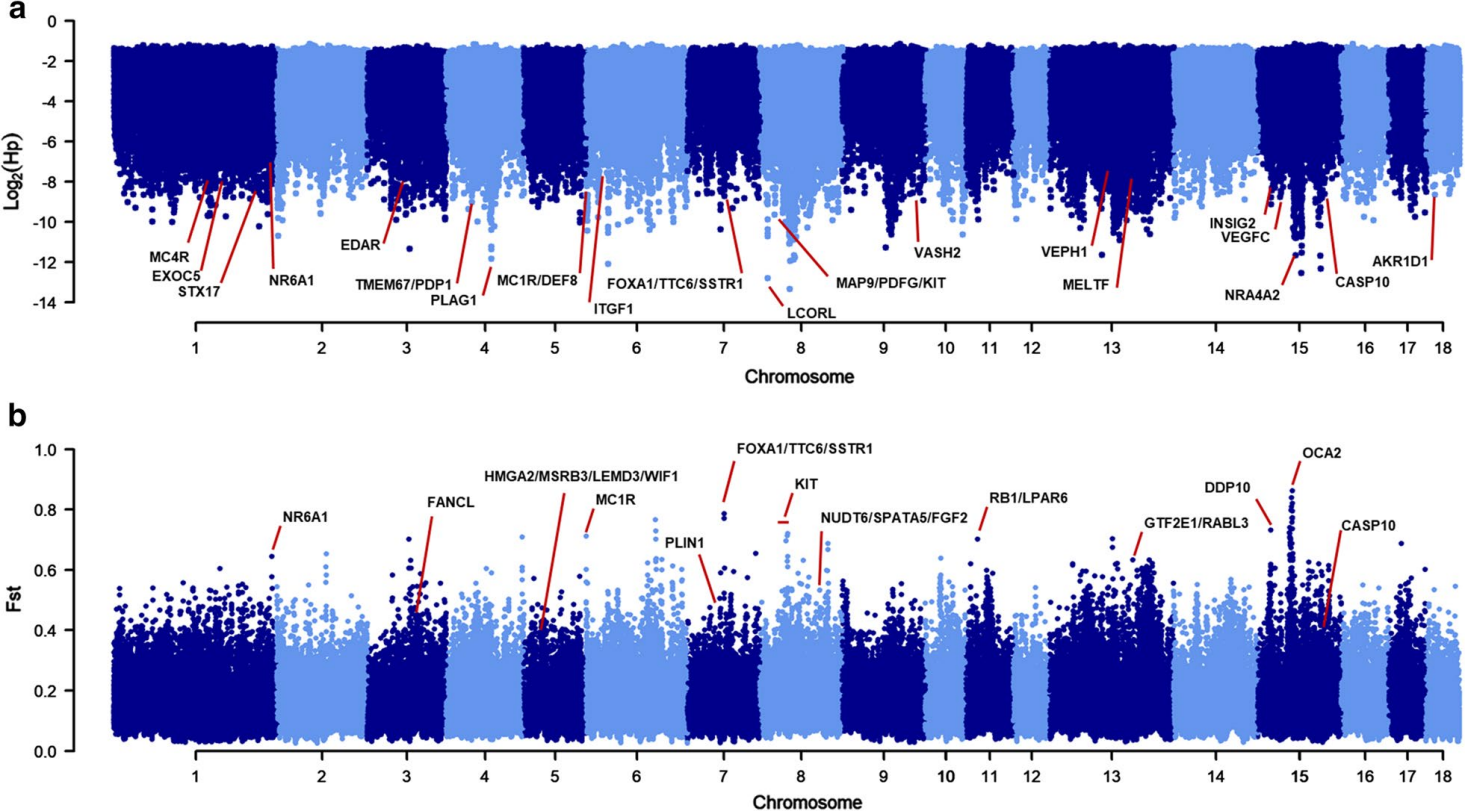
Over-imposed Manhattan plots of the analyses of signatures of selection. **a** H_P_ analysis and **b** averaged pairwise F_ST_ analysis

The H_P_ value of single breeds ranged from 0.094 (Turopolje) to 0.21 (Italian Large White). The average H_P_ value was 0.169 (s.d. = 0.031). Details are in Additional file 1: Table S11. For each population, 12 genome windows (99.95th percentile) were detected as outliers (see Additional file 1: Table S12 and Additional file 2: Figure S5). Figure 3a summarizes the signatures of selection that were identified in all the breeds and in wild boars using this approach. Signatures of selection were detected on 16 chromosomes and five unassembled scaffolds. In total, 68 of 276 (25%) genome windows were shared between two or more (up to seven) populations. Nine genome regions were shared by at least four breeds and were located on *Sus scrofa* (SCC) chromosome SSC1, 4 and 8. The SSC1 region (from 170.3 to 170.4 Mb) did not harbour any annotated gene. The SSC4 window (from 42.9 to 43.0 Mb), which contained the *TMEM67* and *PDP1* genes, was already reported to be involved in the domestication process of European pig breeds [20]. The large region on SSC4 (from 75.5 to 75.9 Mb) was detected in seven pig breeds and harbours the *PLAG1* gene, which was shown to be under positive selection during pig domestication [7]. The SSC8 region (from 12.9 to 13.0 Mb), which was identified in four breeds, contains the *LCORL* gene, which has been already reported to be included in an important signature of selection by Rubin et al. [7] and by Schiavo et al. [25]. Two other genome windows on SSC8 (from 42.6 to 42.7 Mb and from 45.5 to 45.6 Mb), close to the *KIT* gene, harboured two genes, which are reported to be under selection (i.e. *MAP9* and *PDGFC*; [67]).

Other interesting H_P_ signals, which potentially affect traits that could define breed-specific features or production characteristics, included the *MC1R* and *EDAR* genes (affecting hair-related traits [68–71]), *ITFG1* (associated with average daily gain in cattle [72]), *NR4A2* (involved in female reproduction [73]), *MC4R* (affecting fat deposition, growth performances and feed intake [74]), and *NR6A1* (affecting the number of vertebrae [75]).

A relevant signal was detected in Lithuanian White old type, Italian Large White, Italian Landrace and Italian Duroc pigs on SSC15 (from 87.1 to 87.2 Mb), in the region that harbours the *CASP10* gene, confirming a major signature of selection detected by Rubin et al. [7].

H_P_ analyses also highlighted genome windows, which contain several other genes (e.g. *FOXA1*, *INSIG2* and *VEPH1*, and *EXOC5*) that were previously detected in analyses of signatures of selection that compared European domestic pigs against wild boars and Asian pigs [20, 76] (Table 1).

**Table 1:** Genome windows identified by the H_P_ analysis on different porcine chromosomes (SSC) and shared by four or more pig breeds

Genome windows (SSC: start–end bp)	Number of windows	Pig populations	Number of SNPs	Annotated genes (± 200 kb)
1:170,300,001:170,400,001	1	Apulo-Calabrese, Casertana, Krškopolje, Majorcan Black	829	–
4:42,900,001:43,000,001	1	Black Slavonian, Nero Siciliano, Krškopolje, Gascon	683	*TMEM67*^a^, *PDP1*^b^, *FAM92A*^c^, *RBM12B*^c^
4:75,500,001:75,900,001	4	Cinta Senese, Apulo Calabrese, Krškopolje, Lithuanian White old type, Sarda, Italian Large White, Italian Landrace	2356	*CHCHD7*^c^*, SDR16C5*^d^, *MOS*^c^*, PENK*^c^, *TMEM68*^d^, *LOC100626876*^c^, *TGS1*^d^, *LYN*^c^, LOC106510084^c^, *PLAG1*^a^, *XKR4*^d^
8:12,900,001:13,000001	1	Bísara, Schwäbisch-Hällisches Schwein, Italian Large White, Italian Landrace, Majorcan Black	1067	*NCAPG*^b^, *CAF16*^d^, *FAM184B*^d^, *LCORL*^*a*^
8:42,600,001:42,700,001	1	Bísara, Lithuanian White old type, Italian Large White, Italian Landrace	683	*LOC102162630*^d^, *LOC100526059*^d^, *MAP9*^a^, *TLL1*^d^, *LOC100620475*^c^
8:45,500,001:45,600,001	1	Bísara, Lithuanian White old type, Italian Large White, Italian Landrace	1482	*PDGFC*^a^

^a^Genes affecting or associated to several traits and within the reported windows

^b^Genes affecting or associated to several traits

^c^Other genes within the reported windows

^d^Other listed genes are ± 200 kb upstream or downstream the reported windows

### F_ST_ analysis of single breeds

The F_ST_ value of single breeds ranged from 0.088 (Sarda) to 0.202 (Turopolje). Details are in Additional file 1: Table S11. The average F_ST_ value was 0.135 (s.d. = 0.030).

In total, 276 genome windows were considered as outliers [99.95th percentile; (see Additional file 1: Table S13 and Additional file 2: Figure S6). Figure 3b summarizes the genome regions that were identified in all the breeds and in wild boars using this approach. About 7% of these windows (18 windows) were located on five autosomes (SSC5, 6, 8, 9, and 15) and were shared by two or more (up to four) pig breeds (Table 2). Five windows were shared by three or more populations, highlighting three genomic regions: (i) SSC5: 29.3–29.6 Mb, which is characterized by the presence of candidate genes (*LEMD3, WIF1, HMGA2* and *MSRB3*) for ear size and ear position in pigs [77–80], and (ii) SSC5: 30.0–30.1 Mb and (iii) SSC8: 46.6–46.7 Mb, for which no annotated genes were found.

**Table 2:** Genomic windows identified in the single breed F_ST_ analysis on different porcine chromosomes (SSC) and shared by two or more pig breeds and wild boars

Genome windows (SSC: start–end bp)	Pig populations	Annotated genes (± 200 kb)
5:29,300,001–29,400,001	Bísara, Moravka, Sarda	*GNS*^d^, *RASSF3*^d^, *TBC1D30*^d^, *WIF1*^a^, *LOC106510322*^b^, *LEMD3*^a^
5:29,400,001–29,500,001	Alentejana, Bísara, Moravka, Sarda	*TBC1D30*^d^, *LOC10651032*^d^, *WIF1*^c^, *LEMD3*^a^, *MSRB3*^a^
5:29,500,001–29,600,001	Bísara, Moravka, Sarda	*LOC106510322*^d^, *WIF1*^c^, *LEMD3*^c^, *MSRB3*^a^
5:29,700,001–29,800,001	Alentejana, Bísara	*WIF1*^a^, *LEMD3*^a^, *MSRB3*^c^
5:30,000,001–30,100,001	Swallow-Bellied Mangalitsa, Moravka, Sarda	*HMGA2*^a^, *MSRB3*^a^
5:30,100,001–30,200,001	Moravka, Sarda	*HMGA2*^c^
5:30,200,001–30,300,001	Moravka, Sarda	*LLPH*^d^, *TMBIM4*^d^, *HMGA2*^c^
5:30,500,001–30,600,001	Swallow-Bellied Mangalits, Moravka	*HMGA2*^a^, *LLPH*^d^, *HELB*^d^, *GRIP1*^d^, *IRAK3*^b^, *TMBIM4*^b^
6:53,300,001–53,400,001	Lithuanian White old type, Italian Large White	*SELENOW*^d^, *BSPH1*^d^, *EHD2*^d^, *BICRA*^b^, *CRX*^d^, *MEIS3*^d^, *NOP53*^d^, *C5AR2*^d^, *SULT2A1*^d^*, C5AR1*^d^, *ZNF541*^b^, *NAPA*^d^, *SLC8A2*^d^, *DHX34*^d^, *ELSPBP1*^d^, *KPTN*^d^
8:43,800,001–43,900,001	Turopolje, Black Slavonian	*MSMO1*^d^, *LOC110262006*^d^, *CPE*^d^, *LOC110261946*^d^, *TMEM192*^d^, *LOC110261945*^d^, *KLHL2*^b^, *LOC102163658*^d^, *LOC102163398*^d^
8:46,600,001–46,700,001	Swallow-Bellied Mangalitsa, Turopolje, Schwäbisch-Hällisches Schwein	–
8:66,300,001–66,400,001	Lithuanian White old type, Lithuanian Indigenous Wattle	*LOC100515222*^d^, *YTHDC1*^d^, *LOC100515741*^d^, *LOC100516628*^b^, *LOC100624891*^d^, *LOC110262115*^d^, *LOC110262116*^d^, *LOC100623504*^d^, *LOC100515394*^d^, *UGT2B31*^b^
8:66,900,001––67,000,001	Bísara, Sarda	*LOC110262013*^d^, *CABS1*^d^*, LOC110262014*^d^, *ODAM*^d^, *LOC100624541*^d^, *PRR27*^d^, *CSN1S2*^b^, *LOC110262119*^d^, *CSN2*^b^, *CSN1S1*^b^, *SULT1E1*^d^, *CSN3*^d^, *STATH*^b^
9:99,500,001–99,600,001	Nero Siciliano, Majorcan Black	*LOC106504983*^b^, *SEMA3C*^d^, *LOC110255497*^b^, *CD36*^d^, *LOC100511343*^d^
9:99,900,001–100,000,001	Nero Siciliano, Majorcan Black	*GNAT3*^b^, *CD36*^d^, *GNAI1*^d^
15:57,800,001–57,900,001^e^	Mora Romagnola, Italian Duroc	–
15:97,300,001–97,400,001	Schwäbisch-Hällisches Schwein, Wild Boar	–
15:105,300,001–105,400,001	Casertana, Majorcan Black	*TMEM237*^d^, *CDK15*^d^, *ALS2*^b^, *C2CD6*^d^, *MPP4*^b^

^a^Genes affecting or associated to several traits

^b^Genes affecting or associated to several traits and within the reported windows

^c^Other listed genes that are within the reported windows

^d^Other listed genes are ± 200 kb upstream or downstream the reported windows

^e^Region close to the *OCA2* gene

We also detected other interesting F_ST_ signals that are linked to pigmentation processes in genome regions that include *MC1R* (in Black Slavonian; confirming the result of the H_P_ analysis), *KIT* (in Krškopolje, Bísara and Italian Large White), *OCA2* (in Mora Romagnola and Italian Duroc) and *RB1* (in Cinta Senese), which encodes a transcription factor cooperating with MITF in melanocytes [81, 82]. Several other genome windows that harbour obesity-related genes were identified in Krškopolje (including *FANCL*), Gascon (including *DPP10*), Swallow-Bellied Mangalitsa (including *PLIN1*), Schwäbisch-Hällisches Schwein (including *NUDT6*, *SPATA5*, and *FGF2*) and Mora Romagnola (harbouring *GTF2E1* and *RABL3*).

The window including the *CASP10* gene on SSC15 (already identified in the H_P_ analyses) was found for the Majorcan Black and Casertana breeds. This gene is located within a major signature of selection that was previously described by Rubin et al. [7] who compared domesticated breeds vs. wild boars. In addition to the *CASP10* gene-containing window, other peaks differentiated wild boars from the domesticated breeds. For example, signals were detected in the SSC1 region that encompasses *NR6A1*, which was already reported in previous studies [7, 28]. Mutations in *NR6A1* affect the number of vertebrae, which is considered a domestication-derived trait and differentiates wild pig from domestic genetic pools [28, 43, 75]. The *FOXA1* and *TCC6* genes, located on SSC7: 62.4–62.5 Mb, were detected from the wild boar data, as previously described by Rubin et al. [7].

For each pig population, we evaluated the overlap between outlier regions in the F_ST_ analyses and those outliers in the H_P_ analyses. Three signals of signatures of selection detected with the two approaches were in genomic regions of less than 500 kb and encompassed two chromosomes: one was detected in Bísara on SSC8 (from 42.7 to 42.9 Mb) near the *KIT* gene and another one was detected in Gascon on SSC15 (from 21.8 to 22.3 Mb) and included the *DPP10* gene.

### Gene enrichment analyses of breed-derived regions of signatures of selection

To obtain a first functional overview of breed-specific windows of signatures of selection, over-representation analyses were run over the human GWAS catalogue. Twelve gene-phenotype associations, related to nine pig breeds, were retained as statistically valid (Table 3).

**Table 3:** Breed-specific over-represented human-derived phenotypes as defined in the human GWAS catalogue

Breed	Human phenotype	Adjusted *p*-value	Overlapping genes
Black Slavonian	Red vs brown/black hair colour	3.37 × 10^−20^	*MC1R, TCF25, CHMP1A, FANCA, SPIRE2, CDK10, DPEP1, DEF8, CBFA2T3*
Black Slavonian	Low tan response	1.06 × 10^−11^	*DBNDD1, MC1R, CHMP1A, FANCA, SPIRE2, DEF8*
Black Slavonian	Brown vs black hair colour	2.81 × 10^−06^	*MC1R, TCF25, CDK10, DEF8*
Black Slavonian	Blond vs brown/black hair colour	1.49 × 10^−04^	*DBNDD1, MC1R, TCF25, CDK10, SPATA2L*
Cinta Senese	Blood protein levels	2.63 × 10^−02^	*EDAR, GZMM, PRSS57, PRTN3, AZU1, ELANE*
Gascon	Red vs brown/black hair colour	1.83 × 10^−09^	*TCF25, FANCA, SPIRE2, CBFA2T3*
Krškopolje	Height	8.46 × 10^−05^	*LYN, MOS, MC4R, SDR16C5, PLAG1, PENK, CHCHD7, RPS20*
Krškopolje	Heel bone mineral density	4.35 × 10^−02^	*MC4R, PLAG1, ACYP2, SPTBN1*
Italian Landrace	Height	6.91 × 10^−05^	*LYN, MOS, PLAG1, CHCHD7, NCAPG, RPS20, LCORL*
Italian Large White	Height	3.50 × 10^−04^	*LYN, MOS, SDR16C5, GNPTAB, PLAG1, PENK, CHCHD7, RPS20*
Moravka	Blood protein levels	1.89 × 10^−02^	*PRTN3, NRTN, AZU1, CD36, ELANE*
Sarda	Height	2.43 × 10^−03^	*LYN, MOS, PLAG1, CHCHD7, RPS20, SERPINI1*

Coat colour emerged as one of the most important distinctive traits that characterize the Black Slavonian and Gascon breeds. Another interesting phenotype highlighted in this analysis is related to size/height (Table 3), which was over-represented in Krškopolje, Italian Landrace, Italian Large White and Sarda. The 12 genes (Table 3) that contribute to this phenotype spanned genome windows encompassing SSC1, 4, 8 and 13. Genome windows on SSC2, 3 and 9 harboured genes associated to blood protein levels (Table 3). This phenotype was over-represented in the Cinta Senese and Moravka breeds. Another phenotype identified in the enrichment analysis was related to “heel bone mineral density” that emerged from the gene set defined for the Krškopolje breed.

### Comparative analyses of signatures of selection between groups of breeds

In order to complement and dissect the results obtained in the breed-specific analyses, F_ST_ was also used to compare different groups of breeds. Since morphological traits are known to represent important features that differentiate breeds, we considered groups of breeds according to coat colour and size of adult animals. These phenotypic descriptors were regarded as representative and fixed or almost fixed in the breeds analysed [15]. Thus, a few breeds that were not characterized by uniformity of the considered external features were excluded. Detailed information on the classification of the breeds investigated is in Additional file 1: Table S1. In addition, comparative analyses were run for groups that included all domestic breeds or only commercial breeds against wild boars, which were also included in pairwise comparisons with a few breeds to confirm/evaluate further the identified signatures of selection. Detailed information on the groups of breeds/populations and comparisons performed is summarized in Additional file 1: Table S8.

### F_ST_ analysis of breed groups based on different coat colours

Breeds were classified according to the main coat colour patterns and features and then compared to identify major genomic regions that affect these external traits. All the results from the comparative analyses are in Additional file 1: Table S14 and Figure S7.

**Fig. 4:**
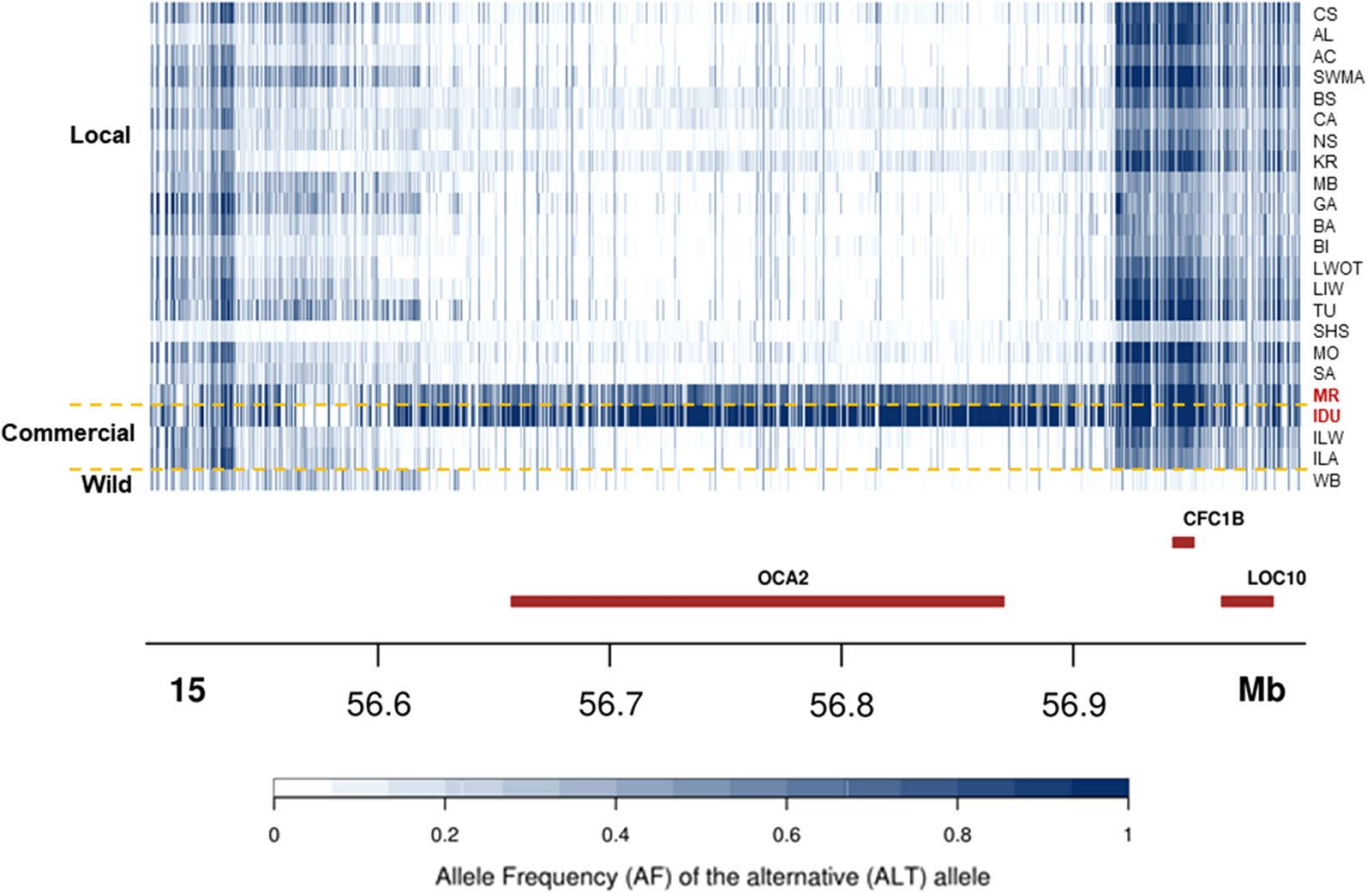
Allele frequency values of SNPs in the *OCA2* region

The breeds classified as belted (Cinta Senese, Krškopolje, Schwäbisch-Hällisches Schwein), even if their belt patterns could not be considered homogeneous, were compared with (i) all other breeds, (ii) all solid coloured breeds, (iii) all solid white coloured breeds (Lithuanian White old type, Italian Large White and Italian Landrace), (iv) all spotted breeds (Basque, Bísara and Lithuanian Indigenous Wattle), and (v) all solid black coloured breeds (Apulo-Calabrese, Swallow-Bellied Mangalitsa, Black Slavonian, Nero Siciliano, Majorcan Black, Gascon and Moravka). In all these comparisons, major F_ST_ peaks were observed in the SSC8 regions that contain *KIT*, which is involved in the determination of this coat colour pattern [7, 83, 84], or close to it, and include the large and complex copy number variation region previously shown to affect this phenotype [7]. In the comparisons of the belted breeds with all the other breeds or with the spotted breeds, major F_ST_ signals were also identified a few Mb away from the *KIT* gene, in a window from 48.5 to 48.6 Mb, without any annotated gene. This latter comparison showed that the most important F_ST_ signal on SSC8 was located downstream from the previous regions (from 66.7 to 66.8 Mb). This window encompasses several annotated genes with unknown functions or that could not be considered as directly involved in coat colour phenotypes, according to current knowledge. This region was also identified in the comparison between spotted and black breeds, which suggests that this window might contain genomic features that affect spotted phenotypes. The results of the comparisons between the two reddish breeds (Italian Duroc and Mora Romagnola) against all breeds of other coat colours and colour patterns confirmed that SSC15 contains a major signature of selection, as evidenced by several emerging peaks from 53.8 to 58.3 Mb and including the *OCA2* gene (Fig. 4).

### F_ST_ analysis of breed groups based on adult body size

**Fig. 5:**
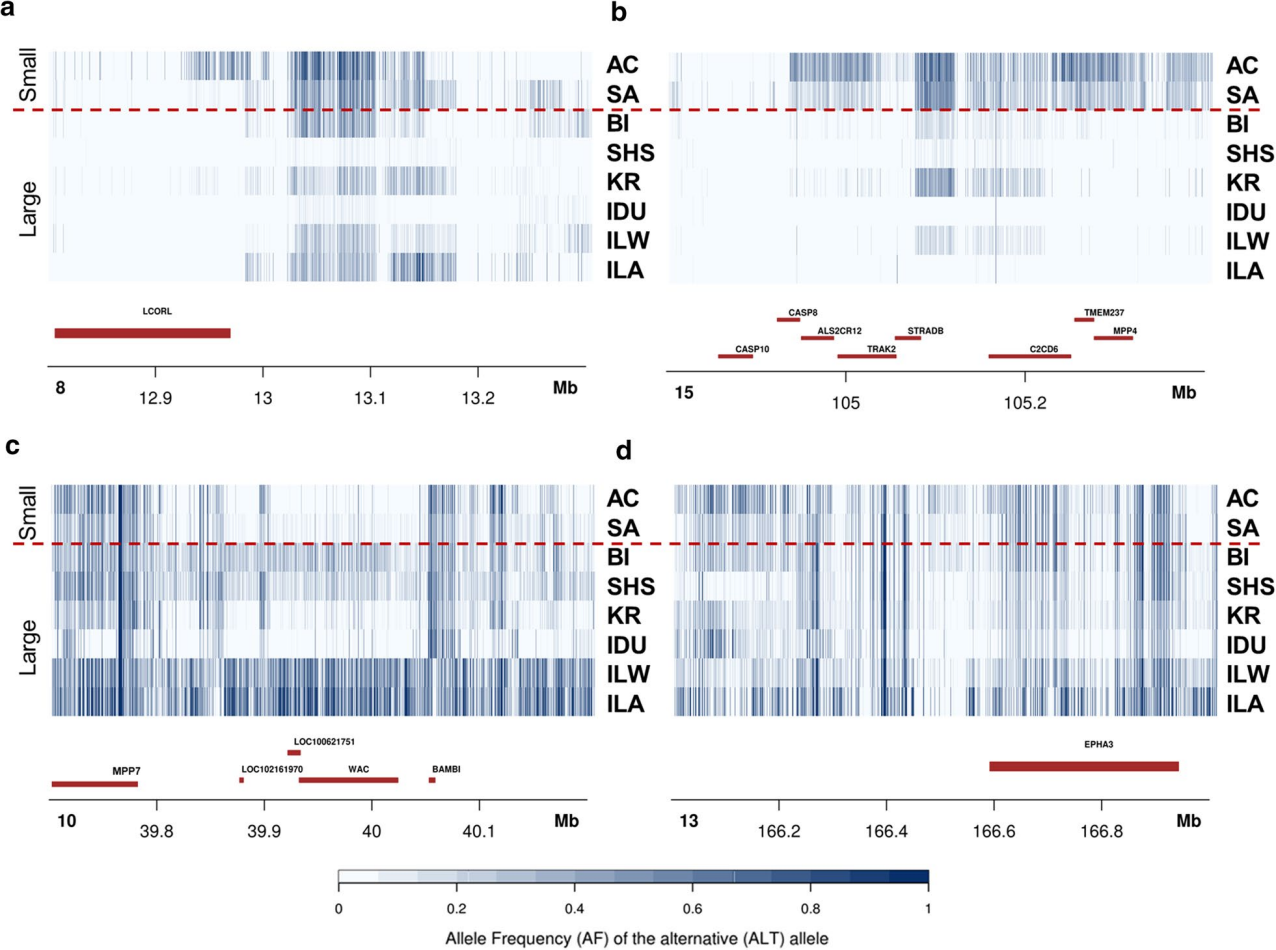
Allele frequency values of SNPs in putative selective sweep regions detected in the F_ST_ analysis of small vs large-sized pig breeds. Major signals were detected on **a** SSC8 that carries the *NCAPG*-*LCORL* gene, **b** SSC15, that carries the *CASP10* gene, **c** SSC10, close to the *MPP7* gene and **d** SSC13, close to the *EPHA3* gene

Signatures of selection that affect body size were evaluated by grouping the pig breeds into three distinct classes: (small size, medium size and large size (see Additional file 1: Tables S1, S8)] and are reported in Additional file 1: Table S14 and Additional file 2: Figure S7. Signatures of selection between the two extreme groups of breeds (small vs. large-sized pigs) were detected on SSC8, 10, 13 and 15. The most interesting F_ST_ signals were on SSC8 (Fig. 5a) and SSC15 (Fig. 5b), in genomic regions that are close to or harbour the *NCAPG*-*LCORL* and *CASP10* genes, respectively. These loci are known to be involved in the determination of body conformation, birth weight and height in humans and several other domesticated and wild animal species [85–89], including wild boars and domestic pigs [7, 25]. The region on SSC10 (Fig. 5c) maps near the *MPP7* gene, which has been linked to number of teats in pigs [90]. On SSC13, three genome windows were identified and the most relevant one (Fig. 5d) was close to the *EPHA3* gene, which is associated with ham weight loss at first salting in Italian Large White pigs [91].

The signal on SSC15 was also detected when middle- and large-sized pig breeds were compared (see Additional file 2: Figure S8a) in addition to other genome windows on SSC1, 2 and 8. Among the windows on SSC1, two (see Additional file 1: Figure S8b, c) harbour genes that are related to body size. The first window harbours the *ARID1B* gene, which is associated with both syndromic and non-syndromic short stature [92]. The second window contains the *MAP3K5* gene (and the nearby *PEX7* gene), which has been suggested as a functional candidate gene for body size in sheep [93]. Another signal was observed on SSC2 (see Additional file 1: Figure S8d), which contains the *PIK3C2A* gene. Mutations in the human *PIK3C2A* gene cause syndromic short stature and skeletal abnormalities [94]. The small vs. medium size comparison revealed a window on scaffold NW_018084901.1, which contains the *SHOX* gene (ENSSSCG00000031933) that encodes the short stature homeobox protein.

### Comparative F_ST_ analyses between commercial and local pig breeds

Commercial pig breeds (Italian Large White, Italian Duroc and Italian Landrace) that have been under intensive selection programmes since the beginning of the 1990’ [95–97] were compared with local pig breeds. Signals were detected on seven chromosomes: SSC1, 5, 9, 10, 11, 13 and 15 (see Additional file 1: Table S14 and Additional file 2: Figure S7). The two windows on SSC1 were close to the *MC4R* gene (~ 160.77 Mb), which is known to affect growth performances and carcass traits in pigs [73, 74].

The window on SSC5 (29.3–29.4 Mb) contains two genes (*WIF1* and *LEMD3*) that are associated with ear size [78, 80]. The region on SSC10 (39.9–40.0 Mb) maps near *MPP7*, which is associated with the number of teats in pigs [90]. The window on SSC13 (167.4–167.5 Mb) is close to *EPHA3* (166.6–166.9 Mb), which is associated with ham weight loss at first salting in Italian Large White pigs [91]. The SSC15 region (105.2–105.3 Mb), which contains several functional genes (*TMEM237*, *C2CD6* and *MPP4*), is close to the previously mentioned region on this chromosome that includes *CASP10*. This gene was reported to be in a signature of selection that was identified by comparing wild boars and domestic pigs [7].

### Comparative F_ST_ analysis between domestic (local) breeds and wild boars

**Fig. 6:**
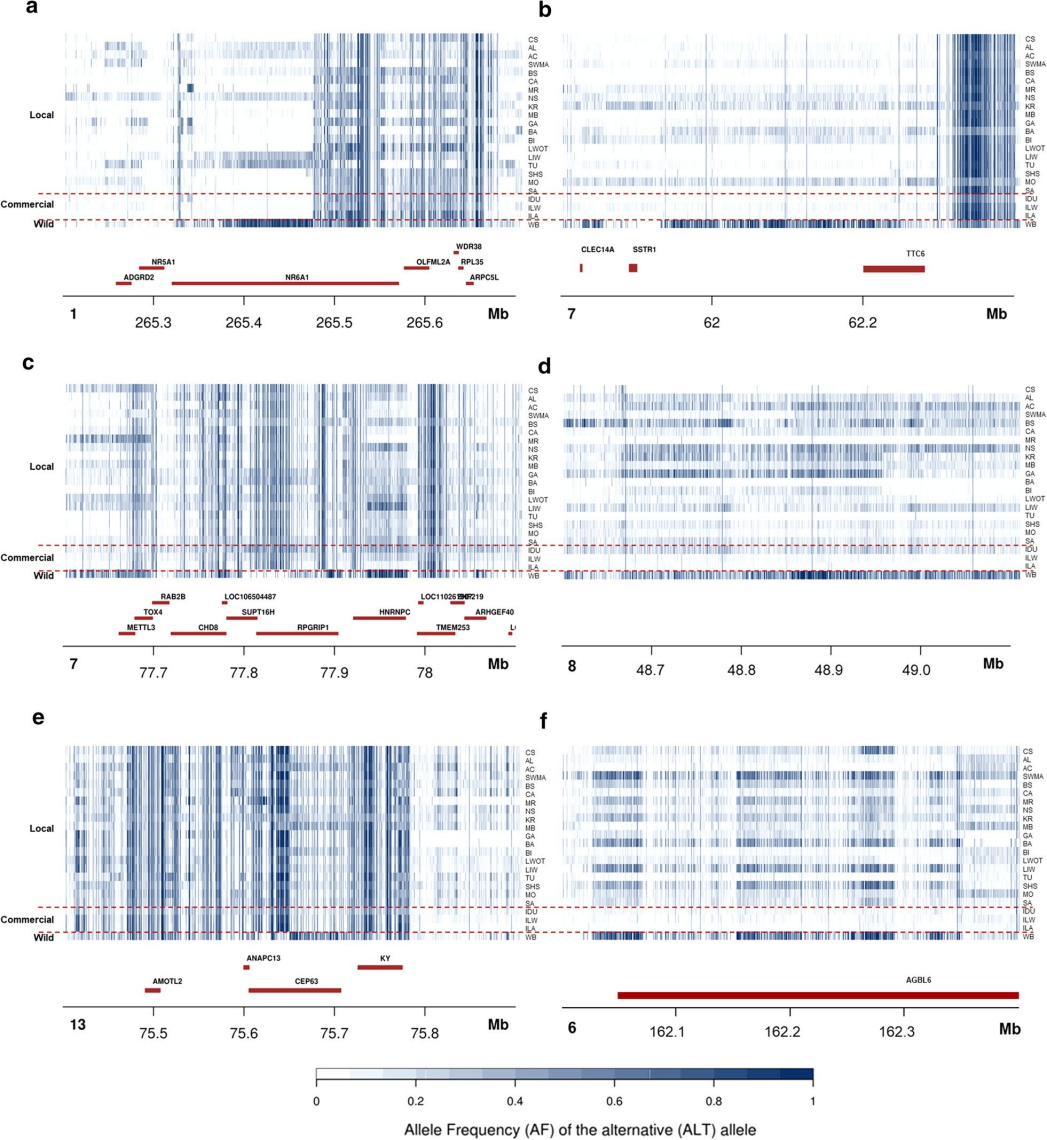
Allele frequency values of SNPs in putative selective sweep regions detected in the F_ST_ analysis of domestic breeds (local) and wild boars. Major signals were detected on **a** SSC1 that carries the *NR6A1* gene, **b** SSC7 that carries the *TCC6, FOXA1* and *SSTR1* genes, **c** SSC7 that carries the *SUPT16H* gene, **d** SSC8, the same region emerging also in the comparisons between spotted breeds, **e** SSC13 that carries the *CEP63* gene and **f** SSC6 that encompasses the *AGBL4* gene

Wild boar whole-genome resequencing data were compared first with data from all domestic breeds. Signatures of selection were identified on SSC1, 7, 8, 13 and 15 (see Additional file 1: Table S14 and Additional file 2: Figure S7). The SSC1 regions were close to two major genes, already mentioned above (*MC4R* and *NR6A1*; Fig. 6a). Two signatures of selection were located on SSC7 (Fig. 6b, c): one close to *TCC6* and *FOXA1*, which were also identified in the single population analysis, and to *SSTR1*, which is involved in the metabolite levels of the 5-HIAA/MHPG ratio [98]. The second SSC7 region contains the *SUPT16H* gene, which encodes a component of the chromatin transcription (FACT) complex and has been suggested to be involved in transcriptional suppression during virus infections and thus in the promotion of virus latency [99], The SSC8 window (Fig. 6d) is the same region that was detected in the comparison between the spotted breeds. The window on SSC13 (Fig. 6e) harbours the *CEP63* gene, which is associated with human height [100]. Finally, the three SSC15 windows identified in this comparison contain no annotated genes.

Considering that autochthonous breeds may have experienced cross-breeding with wild boars (which occurs mainly in extensive production systems [28]), we also compared wild boar sequence data with data from the commercial breeds that are assumed not to have been recently affected by wild boar introgression. Our results were similar to those described for the comparison against all domesticated breeds (i.e. we identified the same SSC1, 7 and 15 regions). In addition, in this analysis, a few other regions emerged: a large region on SSC6 (Fig. 6f) that encompasses the *AGBL4* gene, which is involved in obesity and fat deposition traits [101], and a few other regions on SSC15, including a region close to the previously reported *CASP10* gene.

### Putative deleterious variants in selection sweep regions

The impact of non-synonymous SNPs (nsSNPs) on protein function was evaluated with SIFT, which predicts whether an amino-acid substitution is functionally neutral or deleterious [102]. In total, 18,532 of 149,180 nsSNPs (~ 12.4%) were classified as deleterious to protein function (SIFT score < 0.05). Since a SNP can affect multiple genes and their isoforms, 21,252 deleterious scores were obtained, among which 6599 were equal to zero and 11,396 had a value lower than 0.01. However, interpretation of these SIFT predicted effects should be done with caution, since most of them might be functionally neutral [54].

Alongside nsSNPs, stop gain (N = 968) and stop lost (N = 148) variants were considered potentially deleterious, according to their disrupting effect on the protein function. Allele frequencies (AF) of these putative deleterious variants (N = 19,648; 18,532 nsSNPs + 968 SG + 148 SL) were evaluated relative to the wild boar population, i.e. we considered that the putative ancestral form was the allele, which within the wild boar population had a frequency higher than 0.5 and we quantified their number. We identified 19,395 putative deleterious/disrupting variants (18,290 nsSNPs, 959 SG and 146 SL) out of the 19,648 previously detected variants also present in the wild boar population. Of these 19,395 variants, 1782 (1640 nsSNPs, ~ 9%; 109 SG, ~ 11%; 33 SL, ~ 23%) had the alternative allele as ancestral form (i.e. not in the reference genome).

We also analysed the fraction of variants that showed a marked difference in allele frequency between pig breeds and wild boars (AF > 0.8 in one group, AF < 0.2 in the other, and vice versa). Of the 19,395 inspected variants, we retrieved 86 variants (see Additional file 1: Table S15): 81 nsSNPs, 4 SG and 1 SL, and about 92% of these variants had the alternative allele as the ancestral form. Then, we examined the overlap between these variants and the regions under selection and identified four variants in the following genes: one SNP in *L3HYPDH* (regions identified in the H_P_ analysis of Lithuanian indigenous wattle; with the alternative allele as the ancestral form), one SNP in *OLFML2A* (region of *NR6A1*; with the alternative allele as the ancestral form) and two SNPs in *RPGRIP1* (F_ST_ analysis of wild boar; both SNPs presented the reference allele as the ancestral form).

## Discussion

At the population level, livestock genetic resources have been shaped by a complex interplay between human directly driven or derived genetic events (including artificial selection, introgression, admixture, genetic drift, and bottleneck) and adaptation to a variety of environmental and production conditions [3]. The resulting signatures of selection can be detected at the genome level by analysing and comparing the sequence variation among breeds or populations. In this study, we analysed the distribution of genetic variants in the genome of a European wild boar population and 22 European pig breeds, most of them being autochthonous and unexplored populations. By using whole-genome resequencing of DNA pools, we identified signatures of selection that covered 502 unique genome windows (that were merged into 359 genome regions) and 49.9 Mb (~ 2%) of the *Sus scrofa* genome.

Our results were obtained by using a single breed approach with two statistics (within-breed pooled heterozygosity and fixation index) and a group-based F_ST_ approach, which compared groups of potentially partial homogeneous genomes, as assumed by general phenotypic descriptors or level of domestication/breeding of the grouped breeds. Signals overlapped partially between approaches, methods, breeds and contrasted groups of breeds, providing an interesting picture of genomic patterns distributed in European pig breeds.

Summarizing and combining these results, putatively ancestral related signatures of selection (i.e. wild boar conditions) were detected across all the genome of several autochthonous breeds (Fig. 6). A possible explanation could be a continuous gene flow that might have contributed, at least in part, to the adaptation of these pig genetic resources to a variety of environments and extensive or semi-extensive production systems. The flow might be, in most cases, accidental, derived by the rearing systems that cannot prevent admixture with wild boars. In other cases (i.e. Mora Romagnola, Swallow Bellied Mangalitsa, Basque and Turopolje breeds), this picture reflects the deliberate use of wild boars to constitute or reconstruct these breeds or the fact that these primitive breeds only recently started to differentiate from a wild genetic background. These situations could also explain, in part, their greater rusticity and their usual lower production efficiency compared to commercial breeds.

In spite of this general and potentially recent introgression of wild boar genetic sequences into the domestic pig genome, the comparison with wild boars identified several genome regions that are associated to the domestication state. Rubin et al. [7] had already reported a few of these regions. For example, the region including the *NR6A1* gene on SSC1 showed genetic patterns that differed to some extent between wild boars and domestic breeds, similarly to what was observed for the *CASP10* gene region on SSC15. In this comparison, we also detected a signature of selection in the region around the *MC4R* gene, which, indirectly, confirms the important effect of this gene on productive and economically relevant traits [73] that were selected for during domestication and selection processes. The signals in this region were detected by comparing wild boars with both autochthonous breeds and cosmopolitan breeds, which suggests, at this gene, a gradient of allele frequencies that went from wild state to domesticated and then to selected state. At the putative causative mutation of this gene (c.892G>A; rs81219178), the frequency of allele *A*, which is associated with high growth performances [73, 103], was higher in the commercial breeds than its average frequency in all autochthonous breeds, as was already shown by Muñoz et al. [27]. Wild boars carried only allele *G*. This signature of selection was also confirmed by the increasing trend of the frequency of allele *A* during the decades of intensive selection programmes in commercial breeds [95]. Signatures of selections were also identified in the same gene regions by applying single-breed analyses, which confirmed again the existence of quite differentiated patterns of ancestral/primitive states across breeds. Similar conclusions were also drawn for several other gene regions (including genes not previously reported or genes already reported by others, e.g. *LCORL*, *NR6A1*, *MAP9* and *PLAG1* [7, 75], that emerged only in a few breeds or that emerged by applying one or another statistical approach.

Recently, we analysed SNP chip data that were produced for all the autochthonous breeds included in this study and used a single-SNP single-breed F_ST_ approach to detect signatures of selection in the pig genome [30]. The comparison of SNP chip data with the whole-genome resequencing data obtained in this study clearly showed that the two approaches can capture different types of information (complementary and only in part overlapping). This could be due to statistical and methodological differences between the two studies (we included also three commercial pig breeds) and to the different level of informativity of SNP chip vs sequencing data. Nineteen overlapping (or in proximity; < 500 kb) genome windows in 11 pig breeds were identified (see Additional file 1: Table S16). The fact that these regions were detected by two different methodological approaches and techniques strengthens the involvement of genes that potentially affect traits with breed-specific features or production characteristics. For example, one interesting signature of selection that both approaches captured was in the Alentejana breed on SSC5, it encompasses the *HMGA2/MSRB3/LEMD3/WIF1* [77–80] gene region that is associated with ear conformation. Signatures of selection that were detected by both SNP chip and resequencing data were also observed in Krškopolje (SSC5) and Lithuanian White old type pig breeds (SSC13) and encompass genes that affect fatty acids content (*PLOD2*; [104]) and human height (*PLOD2* and *FOXO3*; [70, 105]).

By comparing whole-genome resequencing data from different homogeneous groups of breeds, it was possible, in most cases, to confirm results that had already been obtained with the single-breed approach. When morphological traits (i.e. coat colours and body size) were used to group breeds, emerging F_ST_ windows provided information on well-known genes that affect these morphological characteristics (i.e. *KIT*, *NCAPG*-*LCORL* and *CASP10* [7, 83, 84]) in addition to the emergence of new evidence. For example, on SSC8, in addition to the *KIT* region, we detected other genomic regions that are strongly associated with pigmentation patterns, and on SSC15, there is strong evidence that the *OCA2* gene region is associated with the reddish coat colour of Duroc [106] and Mora Romagnola breeds. Moreover, the overlap of some of these genome regions with those detected in the single-breed F_ST_ analysis (in relation to the breeds characterizing the investigated groups) strengthens the possible involvement of genome regions that potentially affect phenotypic traits.

Whole-genome resequencing data also provided information on putatively deleterious/disrupting mutations in the genome of the investigated breeds and the wild boar population. A limited fraction of these mutations (0.4%) was detected as highly frequent (AF > 0.8) in the wild boar population, and only four mutations were included in signatures of selection, which suggests that most of the variants with putative functional effects may play a regulatory role, as reported in other studies (i.e. [7]).

## Conclusions

This study mined whole-genome resequencing data that were produced for autochthonous and commercial domestic pig breeds and wild boar populations to identify signatures of selection in the *Sus scrofa* genome that might reflect, at least in part, the genetic diversity present in this livestock species at the European level. With more than 22 million genome variants, we used different statistics and methodologies that allowed us to detect signatures of selections in more than 500 genome regions. These regions harboured genes that can explain part of the phenotypic diversity of the investigated pig populations and their adaptation to different breeding and production systems. Wild boar related signatures of selection are present in many autochthonous breeds in Europe, which suggests that the management of these genetic resources should evaluate the contribution of the ancestral state in defining breed rusticity. Overall, these results will be useful to better decipher the biological mechanisms that underlie the genetic diversity of different pig populations and to design appropriate conservation programmes for maintaining these genetic resources.

## Supplementary information

**Additional file 1: Table S1.** Details on the animals analysed and breeds investigated, including geographical distribution and phenotypic description. **Table S2.** Summary of whole-genome sequencing statistics. **Table S3.** Statistics on SNPs detected in this study. **Table S4.** Statistics on annotated SNPs. Annotation was performed with the Variant Effect Predictor (VEP) tool. **Table S5.** Statistics on the window selection analysis. **Table S6.** Groups of breeds/populations compared in the current study. **Table S7.** Statistics of the genome-wide window-based heterozygosity (H_P_) values and fixation index (F_ST_) values. **Table S8.** Statistics of the genome-wide F_ST_ values between groups of pig breeds/populations based on 100-kb windows. **Table S9.** Pearson’s correlation coefficient (*r*) based on the frequency of the alternative allele. **Table S10.** Single SNP F_ST_ distances between pairs of pig populations. **Table S11.** Within-breed average pooled heterozygosity (H_P_) and fixation index (F_ST_) values. **Table S12.** H_P_ analysis. The genome windows at the extreme lower end of the distributions (99.95th percentile) are presented. **Table S13.** Single-breed F_ST_ analysis. The genome windows at the extreme lower end of the distributions (99.95th percentile) are presented. **Table S14.** Comparative F_ST_ analysis of breed groups. The genome windows at the extreme lower end of the distributions (99.95th percentile) are presented. **Table S15.** Putative deleterious variants that showed a marked allele frequency difference between pig breeds and wild boars (> v80% in one group, < 2 0% in the other, and *vice versa*). **Table S16.** Regions of signatures of selection identified by whole-genome resequencing data produced in this study and SNP chip data produced by Muñoz et al. [30].

**Additional file 2: Figure S1.** Evaluation of the D-statistics for the Kolmogorov–Smirnov test. **Figure S2.** Selection of the window size. (a) The number of windows with less than 10 SNPs over windows of variable size (in the range from 50 to 300-kb) is presented. Red dots represent windows larger than 100 kb, for which the number of windows with less than 10 SNPs started to asymptotically decrease. (b to d) Distribution of the number of SNPs contained in the 50-, 100- and 150-kb windows, respectively. **Figure S3.** F_ST_ based Neighbour-Joining tree. Next to the branches, the bootstrap test values expressed as percentage over 10,000 replicates are indicated in red. **Figure S4.** Mantel test between F_ST_ distance and the geographical distances (based on longitudinal and latitudinal coordinates) among autochthonous pig populations. **Figure S5.** Manhattan plots of the genome-wide H_P_ analyses. Each dot represents a 100-kb genome window. **Figure S6.** Manhattan plots of the genome-wide F_ST_ analyses. Each dot represents a 100-kb genome window. **Figure S7.** Manhattan plots of the genome-wide F_ST_ analysis of breed groups Each dot represents a 100-kb genome window. **Figure S8.** Allele frequencies of SNPs in putative regions of signatures of selection detected in the F_ST_ analysis of middle vs large-sized pig breeds. Major signals were detected on: (a) SSC15 that carries the *CASP10* gene, (b) SSC1 that carries the *ARID1B* gene, (c) SSC1 that carries the *MAP3K5* gene (and the nearby *PEX7*) and (d) SSC2 that carries the *PIK3C2A* gene.

## Data Availability

Sequence data generated and analysed in the current study are available in the EMBL-EBI European Nucleotide Archive (ENA) repository (http://www.ebi.ac.uk/ena), under the study accession PRJEB36830. SNPs were submitted to the EMBL-EBI European Variation Archive (EVA) under the study accession PRJEB36974, analyses ERZ1302242. The datasets generated and/or analysed during the current study are available in the Zenodo repository, 10.5281/zenodo.3670560, and from the corresponding author on reasonable request.
