# Supplementary material for: Whole-genome sequencing of European autochthonous and commercial pig breeds allows the detection of signatures of selection for adaptation of genetic resources to different breeding and production systems
Source: Genet Sel Evol. 2020 Jun 26;52:33. doi: 10.1186/s12711-020-00553-7 (PMC7318759; doi:10.1186/s12711-020-00553-7)
Supplement: Supplementary file 1 — Additional file 1: Table S1. Details on the animals analysed and breeds investigated, including geographical distribution and phenotypic description. Table S2. Summary of whole-genome sequencing statistics. Table S3. Statistics on SNPs detected in this study. Table S4. Statistics on annotated SNPs. Annotation was performed with the Variant Effect Predictor (VEP) tool. Table S5. Statistics on the window selection analysis. Table S6. Groups of breeds/populations compared in the current study. Table S7. Statistics of the genome-wide window-based heterozygosity (HP) values and fixation index (FST) values. Table S8. Statistics of the genome-wide FST values between groups of pig breeds/populations based on 100-kb windows. Table S9. Pearson’s correlation coefficient (r) based on the frequency of the alternative allele. Table S10. Single SNP FST distances between pairs of pig populations. Table S11. Within-breed average pooled heterozygosity (HP) and fixation index (FST) values. Table S12. HP analysis. The genome windows at the extreme lower end of the distributions (99.95th percentile) are presented. Table S13. Single-breed FST analysis. The genome windows at the extreme lower end of the distributions (99.95th percentile) are presented. Table S14. Comparative FST analysis of breed groups. The genome windows at the extreme lower end of the distributions (99.95th percentile) are presented. Table S15. Putative deleterious variants that showed a marked allele frequency difference between pig breeds and wild boars (> v80% in one group, < 2 0% in the other, and vice versa). Table S16. Regions of signatures of selection identified by whole-genome resequencing data produced in this study and SNP chip data produced by Muñoz et al. [30]. [file 12711_2020_553_MOESM1_ESM.docx]

**Additional information**

**Whole-genome sequencing of European autochthonous and commercial pig breeds allows the detection of signatures of selection for adaptation of genetic resources to different breeding and production systems**

Samuele Bovo, Anisa Ribani, Maria Muñoz, Estefania Alves, Jose P. Araujo, Riccardo Bozzi, Marjeta Čandek-Potokar, Rui Charneca, Federica Di Palma, Graham Etherington, Ana I. Fernandez, Fabián García, Juan García-Casco, Danijel Karolyi, Maurizio Gallo, Vladimir Margeta, José Manuel Martins, Marie J. Mercat, Giulia Moscatelli, Yolanda Núñez, Raquel Quintanilla, Čedomir Radović, Violeta Razmaite, Juliette Riquet, Radomir Savić, Giuseppina Schiavo, Graziano Usai, Valerio J. Utzeri, Christoph Zimmer, Cristina Ovilo, Luca Fontanesi

**Table of content**

**Additional File 1: Table S1.** Details on the analysed animals and investigated breeds, including geographical distribution and phenotypic description.

**Additional File 1: Table S2.** Summary of whole-genome sequencing statistics.

**Additional File 1: Table S3.** Statistics on single nucleotide polymorphisms (SNPs) detected in this study.

**Additional File 1: Table S4.** Statistics on annotated single nucleotide polymorphisms (SNPs). Annotation has been performed with the Variant Effect Predictor (VEP) tool.

**Additional File 1: Table S5.** Statistics on the window selection analysis.

**Additional File 1: Table S6.** Groups of breeds/populations compared in the present study.

**Additional File 1: Table S7.** Statistics of the genome-wide window-based Heterozygosity values (H_P_) and Fixation index (F_ST_) values.

**Additional File 1: Table S8.** Statistics of the genome-wide FST values between groups of pig breeds/populations based on 100-kb windows.

**Additional File 1: Table S9.** Pearson’s correlation coefficient (PCC) based on the frequency value of the alternative allele.

**Additional File 1: Table S10.** Single SNP F_ST_ distances between pairs of pig populations.

**Additional File 1: Table S11.** Within breed average pooled heterozygosity (H_P_) and fixation index (F_ST_) values.

**Additional File 1: Table S12.** H_P_ analysis. The genome windows at the extreme lower end of the distributions (99.95^th^ percentile) are presented.

**Additional File 1: Table S13.** Single breed F_ST_ analysis. The genome windows at the extreme lower end of the distributions (99.95^th^ percentile) are presented.

**Additional File 1: Table S14.** Comparative F_ST_ analysis of breed groups. The genome windows at the extreme lower end of the distributions (99.95th percentile) are presented.

**Additional File 1: Table S15.** Putative deleterious variants that showed a marked allele frequency difference between pig breeds and wild boars (>80% in one group, <20% in the other, and *vice versa*).

**Additional File 1: Table S16.** Regions of signatures of selection identified by whole-genome resequencing data produced in this study and SNP chip data produced by Muñoz et al. [30].

**Table S1.** Details on the analysed animals and investigated breeds, including geographical distribution and phenotypic description.

| Breed | Acronym of the breed name | Native Name | Alternative name | Country / Geographic region | Standard coat colour | Coat Colour Classification | Size | Population | Additional Info |
| --- | --- | --- | --- | --- | --- | --- | --- | --- | --- |
| Alentejana | AL | Alentejano,  Alentejana | Alentejano | PT / Southwest of the Iberian Peninsula | Black coat colour and scarce black, blonde or reddish thin hair | black coat colour and scarce black, blonde or reddish thin hair/Solid | Medium | 6464 breeding sows and 510 boars distributed by 137 herds (End of 2017). | <https://www.intechopen.com/books/european-local-pig-breeds-diversity-and-performance-a-study-of-project-treasure/alentejano-pig> |
| Apulo-Calabrese | AC | Apulo Calabrese | Apulo Calabrese,  Calabrese,  Nero Abruzzese,  Nero Calabrese,  Nero dei Lepini,  Nero dei Monti Dauni Meridionali,  Nero dei Monti Lepini,  Nero di Calabria,  Nero di Capitanata,  Nero Lucano,  Nero Maremmano,  Nero Pugliese,  Nero Reatino,  Pugliese | IT / Central-South of Italy (Lazio, Basilicata and Calabria regions) | Solid black | Black/Solid | Medium to small | 489 breeding sows and 93 boars distributed in 45 registered farms (August 2015). | <https://www.intechopen.com/books/european-local-pig-breeds-diversity-and-performance-a-study-of-project-treasure/apulo-calabrese-pig> |
| Basque | BA | Basque | Basque  Pie Noir du Pays Basque  Bigourdan  Béarnais  Basco-Béarnais  Navarrin | FR / Basque Country, a region located in the South-West of France and across Spain border. | Piebald, black and white: black head and rump. | Spotted/- | Medium | 580 breeding sows distributed in 28 registered farms (Jenuary 2017). | <https://www.intechopen.com/books/european-local-pig-breeds-diversity-and-performance-a-study-of-project-treasure/basque-pig> |
| Bísara | BI | Bísaro,  Bísara | Bísaro | PT / North of Portugal | Grey or black and white or spotted | Spotted/- | Large | 5460 breeding sows and 520 boars distributed in189 registered farms (August 2017) | <https://www.intechopen.com/books/european-local-pig-breeds-diversity-and-performance-a-study-of-project-treasure/b-saro-pig> |
| Black Slavonian | BS | Black Slavonian | Fajferica | HR / East Croatia | Solid black | Black/Solid | Medium | 1930 breeding sows and 242 boarsdistributed in 209 registered farms (December 2017). | <https://www.intechopen.com/books/european-local-pig-breeds-diversity-and-performance-a-study-of-project-treasure/black-slavonian-crna-slavonska-pig> |
| Casertana | CA | Casertana | Casertana,  Maiale di Teano,  Teanese,  Pelatella | IT / Central-South of Italy (Campania and Molise regions) | Solid black or dark grey / Hairless | Gray/Solid | Medium | 545 breeding sows and 20 boars distributed in 20 registered farms (August 2015). | <https://www.intechopen.com/books/european-local-pig-breeds-diversity-and-performance-a-study-of-project-treasure/nero-casertano-pig> |
| Cinta Senese | CS | Cinta Senese | Cinta Senese,  Cinta,  Cinto, Cinto Toscano,  Cinturello Umbro,  Cinturino Umbro,  Siena Belted | IT / Central Italy (Tuscany region) | Black with white belt | Belted/- | Medium | 5000 animals distributed in 140 farms | <https://www.intechopen.com/books/european-local-pig-breeds-diversity-and-performance-a-study-of-project-treasure/cinta-senese-pig> |
| Gascon | GA | Gascon | Gascon | FR / Foot of the Pyrénées mountains in the southwest of France. | Black skin and are black wire-haired | Black/Solid | Medium | 1423 breeding sows and 177 breeding males distributed in 64 registered farms (year 2017). | <https://www.intechopen.com/books/european-local-pig-breeds-diversity-and-performance-a-study-of-project-treasure/gascon-pig> |
| Krškopolje | KR | Krškopoljski prašič | Krškopolje pig | SI / Slovenia | Black with white belt of varying size and shape | Belted/- | middle to large sized | 311 breeding sows and 60 boars distributed in 130 registered farms (August 2015). | <https://www.intechopen.com/books/european-local-pig-breeds-diversity-and-performance-a-study-of-project-treasure/kr-kopoljski-pra-i-kr-kopolje-pig-> |
| Lithuanian Indigenous Wattle | LIW | Lietuvos vietines | Lithuanian indigenous wattle | LT / Central part of Lithuania, Baisogala, Radviliškis district | Large black spots on the body Colour variations include black and white, ginger, black and tricoloured | Spotted/multicoloured | Medium | 43 breeding sows and 11 boars distributed in 2 registered farms (December 2017) | <https://www.intechopen.com/books/european-local-pig-breeds-diversity-and-performance-a-study-of-project-treasure/lietuvos-vietin-lithuanian-indigenous-wattle-pig> |
| Lithuanian White Old Type | LWOT | Senojo tipo Lietuvos baltosios | Old type Lithuanian White | LT / Baisogala, Radviliškis district, in the central part of Lithuania | White | White/Solid | Medium | 93 breeding sows and 19 boars distributed in 3 registered farms (December 2017). | <https://www.intechopen.com/books/european-local-pig-breeds-diversity-and-performance-a-study-of-project-treasure/lietuvos-baltosios-senojo-tipo-lithuanian-white-pig> |
| Majorcan Black | MB | Negre Mallorquí | Black Majorcan | ES / Mallorca Island | Black or grey skin colour | Black/Solid | Medium | 969 breeding sows and 54 boars distributed in 59 registered farms (August, 2016). | <https://www.intechopen.com/books/european-local-pig-breeds-diversity-and-performance-a-study-of-project-treasure/negre-mallorqu-majorcan-black-pig> |
| Mora Romagnola | MR | Mora Romagnola | Mora Romagnola | IT / North of Italy (Romagna region) | Dark red/black with paler abdomen | Red/Solid | Medium | 270 breeding sows and 67 boars distributed in the 31 registered farms (August 2015). | <https://www.intechopen.com/books/european-local-pig-breeds-diversity-and-performance-a-study-of-project-treasure/mora-romagnola-pig> |
| Moravka | MO | Moravka | Moravka | RS / Morava Valley of central Serbia | Solid black | Black/Solid | Medium | 265 breeding sows and 15 boars distributed in 25 registered farms (February 2018). | <https://www.intechopen.com/books/european-local-pig-breeds-diversity-and-performance-a-study-of-project-treasure/moravka-pig> |
| Nero Siciliano | NS | Nero Siciliano | Nero dei Nebrodi,  Nero delle Madonie,  Nero dell’Etna | IT / Sicily island | Solid black (a few animals could have white spots) | Black/Solid | Medium | 1103 breeding sows and 124 boars distributed in 87 registered farms (August 2015). | <https://www.intechopen.com/books/european-local-pig-breeds-diversity-and-performance-a-study-of-project-treasure/nero-siciliano-pig> |
| Sarda | SA | Sarda | Sarda | IT / Sardinia island | No fixed coat colour | black, grey, tawny or spotted coat colour / - | Small | 61 breeding sows and 20 boars distributed in 13 registered farms. | <https://www.intechopen.com/books/european-local-pig-breeds-diversity-and-performance-a-study-of-project-treasure/sarda-pig> |
| Schwäbisch-Hällisches Schwein | SHS | Schwäbisch-Hällisches Schwein | Schwaebisch-Haellisches Schwein  Swabian Hall pig,  Swabian Hall swine | DE / Area of Schwäbisch Hall | White in the centre, with a black head and rear and narrow grey bands at the transition from white to black skin. | Belted/- | Medium to large | 350 breeding sows and 35 boars distributed in 15 registered farms (August 2015). | <https://www.intechopen.com/books/european-local-pig-breeds-diversity-and-performance-a-study-of-project-treasure/schw-bisch-h-llisches-pig> |
| Swallow-Bellied Mangalitsa | SBMA | Mangulica | Mangalitsa | RS / Serbia | Grey/black (The three Mangalica breed types are blonde, swallow-belly and red Mangalitsa. ) | Black/Solid | Medium | 925 sows, 605 gilts and 42 boars distributed in 67 registered farm (End of 2017) | <https://www.intechopen.com/books/european-local-pig-breeds-diversity-and-performance-a-study-of-project-treasure/mangalitsa-swallow-belly-mangalitsa-pig> |
| Turopolje | TU | Turopoljska svinja | Turopolje pig | HR / West Croatia | Grey/pale red | distinctive sporadic black spots on a white or gray coat / - | Medium | 116 breeding sows and 14 boars distributed in 12 registered farms (August 2016). | <https://www.intechopen.com/books/european-local-pig-breeds-diversity-and-performance-a-study-of-project-treasure/turopolje-pig-turopoljska-svinja-> |

**Table S2.** Summary of whole-genome sequencing statistics.

| **Population** | **Country** | **No. of read pairs** | **No. of animals** | **Fraction of duplicated reads** | **Breadth of coverage (%)*** | **Depth of coverage (X)*** |
| --- | --- | --- | --- | --- | --- | --- |
| Alentejana | PT | 419,690,476 | 35 | 0.143 | 98.42 | 41.98 |
| Apulo-Calabrese | IT | 418,529,727 | 35 | 0.137 | 98.49 | 42.12 |
| Basque | FR | 407,698,128 | 30 | 0.130 | 98.35 | 39.55 |
| Bísara | PT | 415,284,437 | 35 | 0.126 | 98.51 | 42.44 |
| Black Slavonian | HR | 405,316,112 | 35 | 0.139 | 98.51 | 40.61 |
| Casertana | IT | 435,598,516 | 35 | 0.142 | 98.50 | 43.61 |
| Cinta Senese | IT | 422,120,850 | 35 | 0.139 | 98.47 | 42.42 |
| Gascon | FR | 408,764,207 | 30 | 0.120 | 98.47 | 41.10 |
| Krškopolje | SI | 404,204,144 | 35 | 0.134 | 98.52 | 40.80 |
| Lithuanian Indigenous Wattle | LT | 409,935,460 | 35 | 0.123 | 98.48 | 41.99 |
| Lithuanian White Old Type | LT | 405,822,217 | 35 | 0.123 | 98.45 | 41.62 |
| Majorcan Black | ES | 414,314,159 | 35 | 0.133 | 98.48 | 41.92 |
| Mora Romagnola | IT | 411,095,541 | 35 | 0.138 | 98.45 | 41.21 |
| Moravka | RS | 413,100,992 | 35 | 0.124 | 98.49 | 42.27 |
| Nero Siciliano | IT | 405,812,223 | 35 | 0.137 | 98.44 | 38.92 |
| Sarda | IT | 442,035,147 | 35 | 0.150 | 98.51 | 44.32 |
| Schwäbisch-Hällisches Schwein | DE | 428,982,876 | 35 | 0.135 | 98.48 | 42.69 |
| Swallow-Bellied Mangalitsa | RS | 416,663,891 | 35 | 0.130 | 98.45 | 41.08 |
| Turopolje | HR | 416,663,891 | 35 | 0.124 | 98.36 | 42.61 |
| Italian Duroc | IT | 420,384,723 | 35 | 0.154 | 98.51 | 41.91 |
| Italian Landrace | IT | 442,780,637 | 35 | 0.150 | 98.48 | 44.35 |
| Italian Large White | IT | 450,673,024 | 35 | 0.148 | 98.38 | 45.24 |
| Wild Boar | IT | 164,203,815 | 35 | 0.034 | 98.20 | 11.74 |

*Statistics presented after the removal of duplicated reads.

**Table S3.** Statistics on single nucleotide polymorphisms (SNPs) detected in this study.

| **Deposited/novel** | **Varian type** | **No. of detected SNPs** |
| --- | --- | --- |
| **dbSNP** |  |  |
|  | **Common*** | 19,004,673 |
|  | **Rare^#^** | 3,375,246 |
|  | **Fixed^$^** | 5,295 |
| **Novel** |  |  |
|  | **Common** | 3,475,057 |
|  | **Rare** | 1,442,578 |
|  | **Fixed** | 4,871 |
|  | **Total** | 27,307,720^ |

*Allele Frequency (AF) mean ≥ 0.0143; ^#^AF_mean_ < 0.0143; ^$^Fixed: only the ALT allele is present;

^About 99.6% of these high-quality SNPs was also present in the wild boar DNA pool.

**Table S4.** Statistics on annotated single nucleotide polymorphisms (SNPs). Annotation has been performed with the Variant Effect Predictor (VEP) tool.

| **VEP consequence** | **No. of SNPs** |
| --- | --- |
| Intron variant | 27,723,666 |
| Intergenic variant | 15,604,676 |
| 3’-UTR variant | 469,853 |
| Non-coding transcript variant | 396,126 |
| Synonymous variant | 274,870 |
| Missense variant | 149,180 |
| 5’-UTR variant | 93,507 |
| Splice region variant | 52,788 |
| Non-coding transcript exon variant | 15,757 |
| Stop gained | 1,357 |
| Splice donor variant | 806 |
| Splice acceptor variant | 567 |
| Start lost | 376 |
| Stop lost | 201 |
| Stop retained variant | 144 |
| Coding sequence variant | 107 |
| Mature miRNA Variant | 48 |
| Total* | 44,784,029 |

*VEP took as input a total of 26,732,468 SNPs and gave as output a total of 44,784,029 SNP because co-location of variants.

**Table S5.** Statistics on the window selection analysis.

| **Window size (bp)** | **No. of windows** | **No. of windows with less than 10 SNPs** | **Average number of SNPs/win (Standard deviation)** |
| --- | --- | --- | --- |
| 50000 | 46,940 | 46,231 | 579 (291) |
| 100,000 | 23,666 | 23,267 | 1,130 (566) |
| 150,000 | 15,949 | 15,583 | 1,676 (844) |
| 200,000 | 12,085 | 11,749 | 2,212 (1,125) |

**Table S6.** Groups of breeds/populations compared in the present study.

| Comparison | Breed(s)/population(s) in the first group | Breed(s)/population(s) in the second group |
| --- | --- | --- |
| Belted vs all others | Cinta Senese, Krškopolje, Schwäbisch-Hällisches Schwein | Alentejana, Apulo-Calabrese, Basque, Bísara, Black Slavonian, Casertana, Gascon, Italian Duroc, Italian Landrace, Italian Large White, Lithuanian Indigenous Wattle, Lithuanian White Old Type, Majorcan Black, Mora Romagnola, Moravka, Nero Siciliano, Sarda, Swallow-Bellied Mangalitsa, Turopolje |
| Belted vs solid | Cinta Senese, Krškopolje, Schwäbisch-Hällisches Schwein | Alentejana, Apulo-Calabrese, Black Slavonian, Casertana, Gascon, Italian Duroc ,Mora Romagnola, Moravka, Nero Siciliano, Swallow-Bellied Mangalitsa, Majorcan Black |
| Belted vs spotted | Cinta Senese, Krškopolje, Schwäbisch-Hällisches Schwein | Basque, Bísara and Lithuanian Indigenous Wattle |
| Belted vs solid black | Cinta Senese, Krškopolje, Schwäbisch-Hällisches Schwein | Apulo-Calabrese, Swallow-Bellied Mangalitsa, Black Slavonian, Nero Siciliano, Majorcan Black, Gascon and Moravka |
| Belted vs solid white | Cinta Senese, Krškopolje, Schwäbisch-Hällisches Schwein | Lithuanian White Old Type, Italian Large White, Italian Landrace |
| Red vs all others | Mora Romagnola, Italian Duroc | Alentejana, Apulo-Calabrese, Basque, Bísara, Black Slavonian, Casertana, Cinta Senese, Gascon, Italian Landrace, Italian Large White, Krškopolje, Lithuanian Indigenous Wattle, Lithuanian White Old Type, Majorcan Black, Moravka, Nero Siciliano, Sarda ,Schwäbisch-Hällisches Schwein, Swallow-Bellied Mangalitsa, Turopolje |
| Red v. solid white | Mora Romagnola, Italian Duroc | Lithuanian White Old Type, Italian Large White,Italian Landrace |
| Small vs large sized | Apulo-Calabrese, Sarda | Bísara, Italian Duroc, Italian Landrace, Italian Large White, Krškopolje, Schwäbisch-Hällisches Schwein |
| Middle vs large sized | Alentejana, Basque, Black Slavonian, Casertana, Cinta Senese, Gascon, Lithuanian Indigenous Wattle, Lithuanian White Old Type, Majorcan Black, Mora Romagnola, Moravka, Nero Siciliano, Swallow-Bellied Mangalitsa, Turopolje | Bísara, Italian Duroc, Italian Landrace, Italian Large White, Krškopolje, Schwäbisch-Hällisches Schwein |
| Cosmopolitan-derived vs local | Italian Duroc, Italian Large White, Italian Landace | Alentejana, Apulo-Calabrese, Basque, Bísara, Black Slavonian, Casertana, Cinta Senese, Gascon, Krškopolje, Lithuanian Indigenous Wattle, Lithuanian White Old Type, Majorcan Black, Mora Romagnola, Moravka, Nero Siciliano, Sarda, Schwäbisch-Hällisches Schwein, Swallow-Bellied Mangalitsa, Turopolje |
| Wild Boar vs local | Wild Boar | Alentejana, Apulo-Calabrese, Basque, Bísara, Black Slavonian, Casertana, Cinta Senese, Gascon, Krškopolje, Lithuanian Indigenous Wattle, Lithuanian White Old Type, Majorcan Black, Mora Romagnola, Moravka, Nero Siciliano, Sarda, Schwäbisch-Hällisches Schwein, Swallow-Bellied Mangalitsa, Turopolje |

**Table S7.** Statistics of the genome-wide window-based Heterozygosity values (H_P_) and Fixation index (F_ST_) values.

|  | **Log10(H_P_)** | | | | | | **F_ST_** | | | | | |
| --- | --- | --- | --- | --- | --- | --- | --- | --- | --- | --- | --- | --- |
| **Population** | **Mean** | **sd^1^** | **median** | **Min^2^** | **Max^3^** | **Th^4^** | **Mean** | **sd^1^** | **median** | **Min^2^** | **Max^3^** | **Th^4^** |
| Alentejana | 3.138 | 1.025 | 2.914 | 1.199 | 8.693 | 7.684 | 0.129 | 0.039 | 0.123 | 0.017 | 0.621 | 0.349 |
| Apulo-Calabrese | 2.729 | 0.949 | 2.490 | 1.140 | 9.878 | 7.894 | 0.130 | 0.044 | 0.123 | 0.010 | 0.464 | 0.394 |
| Basque | 3.307 | 1.362 | 2.949 | 1.151 | 11.223 | 9.270 | 0.170 | 0.069 | 0.156 | 0.025 | 0.767 | 0.609 |
| Bísara | 2.518 | 0.787 | 2.308 | 1.140 | 10.369 | 7.164 | 0.105 | 0.032 | 0.101 | 0.016 | 0.534 | 0.324 |
| Black Slavonian | 2.556 | 0.831 | 2.343 | 1.150 | 7.996 | 6.982 | 0.118 | 0.038 | 0.112 | 0.016 | 0.712 | 0.415 |
| Casertana | 2.696 | 0.927 | 2.450 | 1.149 | 8.704 | 7.474 | 0.126 | 0.042 | 0.119 | 0.018 | 0.447 | 0.382 |
| Cinta Senese | 2.981 | 1.017 | 2.755 | 1.205 | 8.717 | 7.987 | 0.127 | 0.045 | 0.119 | 0.016 | 0.702 | 0.407 |
| Gascon | 2.831 | 1.127 | 2.491 | 1.148 | 9.258 | 8.325 | 0.143 | 0.054 | 0.133 | 0.016 | 0.732 | 0.517 |
| Krškopolje | 2.451 | 0.795 | 2.234 | 1.142 | 9.082 | 7.246 | 0.112 | 0.036 | 0.106 | 0.019 | 0.448 | 0.323 |
| Lithuanian Indigenous Wattle | 2.633 | 0.965 | 2.349 | 1.201 | 8.586 | 7.934 | 0.131 | 0.044 | 0.123 | 0.024 | 0.475 | 0.395 |
| Lithuanian White Old Type | 2.594 | 1.003 | 2.295 | 1.191 | 11.835 | 8.289 | 0.140 | 0.056 | 0.127 | 0.017 | 0.532 | 0.457 |
| Majorcan Black | 2.962 | 0.918 | 2.769 | 1.159 | 10.707 | 7.161 | 0.117 | 0.037 | 0.112 | 0.022 | 0.558 | 0.358 |
| Mora Romagnola | 3.673 | 1.636 | 3.238 | 1.134 | 11.656 | 10.327 | 0.188 | 0.079 | 0.170 | 0.017 | 0.687 | 0.584 |
| Moravka | 2.537 | 0.762 | 2.352 | 1.182 | 8.015 | 6.749 | 0.100 | 0.030 | 0.097 | 0.018 | 0.394 | 0.302 |
| Nero Siciliano | 2.580 | 0.719 | 2.420 | 1.189 | 7.516 | 6.058 | 0.093 | 0.025 | 0.091 | 0.017 | 0.375 | 0.244 |
| Sarda | 2.412 | 0.655 | 2.251 | 1.207 | 8.972 | 5.869 | 0.086 | 0.021 | 0.085 | 0.019 | 0.229 | 0.190 |
| Schwäbisch-Hällisches Schwein | 2.578 | 0.936 | 2.303 | 1.144 | 9.879 | 7.721 | 0.129 | 0.044 | 0.121 | 0.022 | 0.533 | 0.416 |
| Swallow-Bellied Mangalitsa | 3.105 | 1.059 | 2.863 | 1.135 | 9.340 | 8.251 | 0.139 | 0.045 | 0.132 | 0.016 | 0.549 | 0.429 |
| Turopolje | 4.168 | 1.735 | 3.829 | 1.199 | 13.327 | 10.794 | 0.199 | 0.078 | 0.184 | 0.038 | 0.720 | 0.649 |
| Italian Duroc | 3.075 | 1.409 | 2.625 | 1.156 | 12.539 | 10.594 | 0.169 | 0.075 | 0.152 | 0.016 | 0.862 | 0.729 |
| Italian Landrace | 2.423 | 0.820 | 2.181 | 1.172 | 10.571 | 7.103 | 0.110 | 0.039 | 0.102 | 0.014 | 0.420 | 0.334 |
| Italian Large White | 2.938 | 1.206 | 2.651 | 1.169 | 12.788 | 8.861 | 0.168 | 0.081 | 0.152 | 0.019 | 0.646 | 0.613 |
| Wild Boar | 3.434 | 1.187 | 3.159 | 1.230 | 9.975 | 8.625 | 0.171 | 0.063 | 0.159 | 0.044 | 0.786 | 0.576 |

^1^Standard deviation; ^2^Minimum; ^3^Maximum; ^4^Value representing the 99.95^th^ percentile of the distribution and used as threshold to detect selective sweep regions.

**Table S8.** Statistics of the genome-wide F_ST_ values between groups of pig breeds/populations based on 100-kb windows.

| **Comparison** | **Mean** | **sd^1^** | **median** | **Min^2^** | **Max^3^** | **Th^4^** |
| --- | --- | --- | --- | --- | --- | --- |
| Red vs. solid white | 0.125 | 0.075 | 0.110 | 0.003 | 0.790 | 0.657 |
| Red vs. all others | 0.073 | 0.047 | 0.062 | 0.000 | 0.667 | 0.462 |
| Belted vs. solid white | 0.069 | 0.045 | 0.058 | 0.000 | 0.547 | 0.359 |
| Belted vs. spotted | 0.047 | 0.026 | 0.041 | 0.000 | 0.336 | 0.211 |
| Belted vs. solid black | 0.033 | 0.019 | 0.029 | 0.000 | 0.261 | 0.146 |
| Belted vs. solid | 0.031 | 0.018 | 0.027 | 0.001 | 0.235 | 0.148 |
| Belted vs. all others | 0.027 | 0.015 | 0.024 | 0.001 | 0.207 | 0.124 |
| Middle vs. large sized | 0.029 | 0.018 | 0.024 | 0.000 | 0.242 | 0.142 |
| Small vs. large sized | 0.038 | 0.023 | 0.033 | 0.000 | 0.312 | 0.170 |
| Commercial vs. local | 0.043 | 0.028 | 0.036 | 0.000 | 0.267 | 0.212 |
| Wild Boar vs. local | 0.086 | 0.046 | 0.076 | 0.001 | 0.557 | 0.403 |

^1^Standard deviation; ^2^Minimum; ^3^Maximum; ^4^Value representing the 99.95^th^ percentile of the distribution and used as threshold to detect selective sweep regions.

**Table S9**. Pearson’s correlation coefficient (*r*) based on the frequency value of the alternative allele.

| **Breed** | ***r*** | **SNPs*** |
| --- | --- | --- |
| Alentejana | 0.96 | 40,614 |
| Apulo-Calabrese | 0.96 | 45,354 |
| Basque | 0.97 | 37,531 |
| Bísara | 0.93 | 46,948 |
| Black Slavonian | 0.92 | 46,730 |
| Casertana | 0.96 | 44,813 |
| Cinta Senese | 0.97 | 46,674 |
| Gascon | 0.96 | 42,319 |
| Krškopolje | 0.94 | 46,625 |
| Lithuanian Indigenous Wattle | 0.95 | 44,376 |
| Lithuanian White Old Type | 0.93 | 45,163 |
| Majorcan Black | 0.96 | 44,895 |
| Mangulica | 0.96 | 40,278 |
| Mora Romagnola | 0.97 | 35,238 |
| Moravka | 0.95 | 47,063 |
| Nero Siciliano | 0.93 | 47,283 |
| Sarda | 0.92 | 47,540 |
| Schwäbisch-Hällisches Schwein | 0.91 | 46,946 |
| Turopolje | 0.98 | 39,194 |

*No. of SNPs used to compute the correlation coefficient.

**Table S10.** Single SNP F_ST_ distances between pairs of pig populations.

|  | **CS** | **AL** | **AC** | **SBM** | **BS** | **CA** | **MR** | **NS** | **KR** | **IDU** | **MB** | **GA** | **BA** | **BI** | **LWOT** | **LIW** | **TU** | **SHS** | **MO** | **SA** | **ILW** | **ILA** | **WB** |
| --- | --- | --- | --- | --- | --- | --- | --- | --- | --- | --- | --- | --- | --- | --- | --- | --- | --- | --- | --- | --- | --- | --- | --- |
| **CS** | -0.030 | 0.111 | 0.127 | 0.126 | 0.116 | 0.123 | 0.182 | 0.085 | 0.114 | 0.168 | 0.103 | 0.145 | 0.170 | 0.103 | 0.146 | 0.133 | 0.190 | 0.131 | 0.098 | 0.084 | 0.116 | 0.174 | 0.152 |
| **AL** | 0.111 | -0.031 | 0.130 | 0.110 | 0.111 | 0.124 | 0.192 | 0.075 | 0.120 | 0.176 | 0.082 | 0.148 | 0.170 | 0.105 | 0.158 | 0.140 | 0.186 | 0.137 | 0.098 | 0.084 | 0.129 | 0.188 | 0.140 |
| **AC** | 0.127 | 0.130 | -0.030 | 0.142 | 0.117 | 0.125 | 0.194 | 0.091 | 0.112 | 0.165 | 0.119 | 0.151 | 0.178 | 0.102 | 0.138 | 0.133 | 0.205 | 0.127 | 0.101 | 0.084 | 0.109 | 0.168 | 0.171 |
| **SBM** | 0.126 | 0.110 | 0.142 | -0.031 | 0.116 | 0.135 | 0.208 | 0.091 | 0.128 | 0.189 | 0.104 | 0.160 | 0.184 | 0.117 | 0.167 | 0.151 | 0.202 | 0.146 | 0.097 | 0.095 | 0.136 | 0.195 | 0.157 |
| **BS** | 0.116 | 0.111 | 0.117 | 0.116 | -0.032 | 0.111 | 0.180 | 0.076 | 0.097 | 0.151 | 0.101 | 0.131 | 0.159 | 0.091 | 0.129 | 0.117 | 0.185 | 0.112 | 0.081 | 0.071 | 0.100 | 0.158 | 0.152 |
| **CA** | 0.123 | 0.124 | 0.125 | 0.135 | 0.111 | -0.030 | 0.185 | 0.085 | 0.106 | 0.163 | 0.112 | 0.144 | 0.173 | 0.099 | 0.134 | 0.127 | 0.200 | 0.125 | 0.095 | 0.080 | 0.103 | 0.162 | 0.165 |
| **MR** | 0.182 | 0.192 | 0.194 | 0.208 | 0.180 | 0.185 | -0.032 | 0.148 | 0.167 | 0.203 | 0.176 | 0.213 | 0.247 | 0.161 | 0.203 | 0.194 | 0.283 | 0.192 | 0.160 | 0.141 | 0.167 | 0.230 | 0.246 |
| **NS** | 0.085 | 0.075 | 0.091 | 0.091 | 0.076 | 0.085 | 0.148 | -0.033 | 0.077 | 0.133 | 0.067 | 0.106 | 0.132 | 0.067 | 0.104 | 0.095 | 0.152 | 0.092 | 0.061 | 0.045 | 0.076 | 0.136 | 0.114 |
| **KR** | 0.114 | 0.120 | 0.112 | 0.128 | 0.097 | 0.106 | 0.167 | 0.077 | -0.032 | 0.134 | 0.107 | 0.126 | 0.156 | 0.083 | 0.114 | 0.110 | 0.187 | 0.093 | 0.077 | 0.063 | 0.086 | 0.147 | 0.159 |
| **IDU** | 0.168 | 0.176 | 0.165 | 0.189 | 0.151 | 0.163 | 0.203 | 0.133 | 0.134 | -0.030 | 0.161 | 0.190 | 0.222 | 0.141 | 0.183 | 0.171 | 0.258 | 0.168 | 0.136 | 0.117 | 0.147 | 0.210 | 0.226 |
| **MB** | 0.103 | 0.082 | 0.119 | 0.104 | 0.101 | 0.112 | 0.176 | 0.067 | 0.107 | 0.161 | -0.032 | 0.135 | 0.156 | 0.094 | 0.141 | 0.126 | 0.172 | 0.122 | 0.087 | 0.073 | 0.114 | 0.171 | 0.129 |
| **GA** | 0.145 | 0.148 | 0.151 | 0.160 | 0.131 | 0.144 | 0.213 | 0.106 | 0.126 | 0.190 | 0.135 | -0.033 | 0.166 | 0.118 | 0.153 | 0.146 | 0.226 | 0.142 | 0.114 | 0.099 | 0.120 | 0.180 | 0.193 |
| **BA** | 0.170 | 0.170 | 0.178 | 0.184 | 0.159 | 0.173 | 0.247 | 0.132 | 0.156 | 0.222 | 0.156 | 0.166 | -0.036 | 0.146 | 0.189 | 0.181 | 0.258 | 0.172 | 0.141 | 0.127 | 0.154 | 0.212 | 0.222 |
| **BI** | 0.103 | 0.105 | 0.102 | 0.117 | 0.091 | 0.099 | 0.161 | 0.067 | 0.083 | 0.141 | 0.094 | 0.118 | 0.146 | -0.032 | 0.106 | 0.100 | 0.176 | 0.099 | 0.072 | 0.057 | 0.076 | 0.137 | 0.146 |
| **LWOT** | 0.146 | 0.158 | 0.138 | 0.167 | 0.129 | 0.134 | 0.203 | 0.104 | 0.114 | 0.183 | 0.141 | 0.153 | 0.189 | 0.106 | -0.033 | 0.115 | 0.226 | 0.129 | 0.107 | 0.087 | 0.078 | 0.143 | 0.199 |
| **LIW** | 0.133 | 0.140 | 0.133 | 0.151 | 0.117 | 0.127 | 0.194 | 0.095 | 0.110 | 0.171 | 0.126 | 0.146 | 0.181 | 0.100 | 0.115 | -0.033 | 0.214 | 0.124 | 0.100 | 0.082 | 0.089 | 0.152 | 0.182 |
| **TU** | 0.190 | 0.186 | 0.205 | 0.202 | 0.185 | 0.200 | 0.283 | 0.152 | 0.187 | 0.258 | 0.172 | 0.226 | 0.258 | 0.176 | 0.226 | 0.214 | -0.032 | 0.207 | 0.167 | 0.152 | 0.190 | 0.251 | 0.246 |
| **SHS** | 0.131 | 0.137 | 0.127 | 0.146 | 0.112 | 0.125 | 0.192 | 0.092 | 0.093 | 0.168 | 0.122 | 0.142 | 0.172 | 0.099 | 0.129 | 0.124 | 0.207 | -0.032 | 0.093 | 0.080 | 0.101 | 0.161 | 0.178 |
| **MO** | 0.098 | 0.098 | 0.101 | 0.097 | 0.081 | 0.095 | 0.160 | 0.061 | 0.077 | 0.136 | 0.087 | 0.114 | 0.141 | 0.072 | 0.107 | 0.100 | 0.167 | 0.093 | -0.033 | 0.054 | 0.079 | 0.140 | 0.136 |
| **SA** | 0.084 | 0.084 | 0.084 | 0.095 | 0.071 | 0.080 | 0.141 | 0.045 | 0.063 | 0.117 | 0.073 | 0.099 | 0.127 | 0.057 | 0.087 | 0.082 | 0.152 | 0.080 | 0.054 | -0.029 | 0.058 | 0.120 | 0.121 |
| **ILW** | 0.116 | 0.129 | 0.109 | 0.136 | 0.100 | 0.103 | 0.167 | 0.076 | 0.086 | 0.147 | 0.114 | 0.120 | 0.154 | 0.076 | 0.078 | 0.089 | 0.190 | 0.101 | 0.079 | 0.058 | -0.028 | 0.083 | 0.164 |
| **ILA** | 0.174 | 0.188 | 0.168 | 0.195 | 0.158 | 0.162 | 0.230 | 0.136 | 0.147 | 0.210 | 0.171 | 0.180 | 0.212 | 0.137 | 0.143 | 0.152 | 0.251 | 0.161 | 0.140 | 0.120 | 0.083 | -0.028 | 0.225 |
| **WB** | 0.152 | 0.140 | 0.171 | 0.157 | 0.152 | 0.165 | 0.246 | 0.114 | 0.159 | 0.226 | 0.129 | 0.193 | 0.222 | 0.146 | 0.199 | 0.182 | 0.246 | 0.178 | 0.136 | 0.121 | 0.164 | 0.225 | -0.125 |

CS: Cinta Senese; AL: Alentejana; AC: Apulo-Calabrese; SBM: Swallow-Bellied Mangalitsa; BS: Black Slavonian; CA: Casertana; MR: Mora Romagnola; NS: Nero Siciliano; KR: Krškopolje; IDU: Italian Duroc; MB: Majorcan Black; GA: Gascon; BA: Basque; BI: Bísara; LWOT: Lithuanian White Old Type; LIW: Lithuanian Indigenous Wattle; TU: Turopolje; SHS: Schwäbisch-Hällisches Schwein; MO: Moravka; SA: Sarda; ILW: Italian Large White; ILA: Italian Landrace; WB: Wild Boar.

**Table S11.** Within breed average pooled heterozygosity (H_P_) and fixation index (F_ST_) values.

| **Pig population** | **H_P_** | **F_ST_** |
| --- | --- | --- |
| Alentejana | 0.139 | 0.128 |
| Apulo-Calabrese | 0.178 | 0.130 |
| Basque | 0.139 | 0.169 |
| Bísara | 0.196 | 0.105 |
| Black Slavonian | 0.193 | 0.118 |
| Casertana | 0.180 | 0.125 |
| Cinta Senese | 0.154 | 0.127 |
| Gascon | 0.174 | 0.143 |
| Krškopolje | 0.205 | 0.112 |
| Lithuanian Indigenous Wattle | 0.190 | 0.130 |
| Lithuanian White Old Type | 0.151 | 0.117 |
| Majorcan Black | 0.143 | 0.139 |
| Mora Romagnola | 0.122 | 0.188 |
| Moravka | 0.192 | 0.100 |
| Nero Siciliano | 0.185 | 0.093 |
| Sarda | 0.204 | 0.086 |
| Schwäbisch-Hällisches Schwein | 0.195 | 0.129 |
| Swallow-Bellied Mangalitsa | 0.195 | 0.140 |
| Turopolje | 0.094 | 0.199 |
| Italian Duroc | 0.162 | 0.169 |
| Italian Landrace | 0.169 | 0.168 |
| Italian Large White | 0.210 | 0.110 |
| Wild Boar | 0.121 | 0.171 |

**Table S12.** H_P_ analysis. The genome windows at the extreme lower end of the distributions (99.95^th^ percentile) are presented.

| **Pig population** | **Genome Windows (SSC:start-end bp)** | **H_P_** | **-Log10(H_P_)** | **No. of SNPs** | **Annotated genes** | **Annotated genes (±200 Kb)** |
| --- | --- | --- | --- | --- | --- | --- |
| Alentejana | 1:53100001-53200001 | 0.0043 | -7.86 | 1525 | LOC100622980 | CEP162;MRAP2;DDX43;KCNQ5;KHDC3L;DPPA5;LOC100511846;LOC100622980;OOEP |
| Alentejana | 1:160900001-161000001 | 0.0044 | -7.84 | 798 | - | PMAIP1;LOC110261667;MC4R |
| Alentejana | 1:241700001-241800001 | 0.0035 | -8.14 | 548 | ERP44;STX17 | NR4A3;ERP44;INVS;STX17 |
| Alentejana | 1:241900001-242000001 | 0.0037 | -8.06 | 688 | INVS | ERP44;INVS;TEX10;STX17;MSANTD3 |
| Alentejana | 2:70200001-70300001 | 0.0045 | -7.80 | 635 | ACP5 | EPOR;ACP5;CNN1;PLPPR2;ELAVL3;PRKCSH;RGL3;LOC100624806;TSPAN16;ELOF1;CCDC151;RAB3D;TMEM205;CCDC159;SWSAP1;ZNF653;ECSIT |
| Alentejana | 3:119300001-119400001 | 0.0047 | -7.72 | 678 | - | LOC100516797;RDH14 |
| Alentejana | 4:51800001-51900001 | 0.0045 | -7.79 | 1002 | RALYL | RALYL;LOC100156775;LOC100522735 |
| Alentejana | 5:95600001-95700001 | 0.0039 | -8.01 | 1963 | MGAT4C | MGAT4C |
| Alentejana | 5:97400001-97500001 | 0.0024 | -8.69 | 1664 | - | - |
| Alentejana | 7:55400001-55500001 | 0.0040 | -7.96 | 329 | AP3S2;ARPIN | AP3S2;TICRR;WDR93;KIF7;ARPIN;ZNF710;PEX11A;ANPEP;IDH2;MESP1;MESP2;PLIN1 |
| Alentejana | 11:31300001-31400001 | 0.0042 | -7.89 | 1065 | - | - |
| Alentejana | 13:87200001-87300001 | 0.0039 | -8.01 | 494 | - | - |
| Apulo Calabrese | 1:100400001-100500001 | 0.0039 | -8.00 | 950 | ME2 | ELAC1;SMAD4;LOC102159046;MRO;MAPK4;ME2;MEX3C |
| Apulo Calabrese | 1:136500001-136600001 | 0.0034 | -8.22 | 610 | SCG5;ARHGAP11A | SCG5;GREM1;FMN1;GJD2;ARHGAP11A |
| Apulo Calabrese | 1:160900001-161000001 | 0.0038 | -8.05 | 798 | - | PMAIP1;LOC110261667;MC4R |
| Apulo Calabrese | 1:161000001-161100001 | 0.0029 | -8.42 | 1062 | - | PMAIP1;LOC110261667 |
| Apulo Calabrese | 1:170100001-170200001 | 0.0040 | -7.98 | 612 | - | FBXO33 |
| Apulo Calabrese | 1:170200001-170300001 | 0.0033 | -8.24 | 821 | - | - |
| Apulo Calabrese | 1:170300001-170400001 | 0.0031 | -8.32 | 829 | - | - |
| Apulo Calabrese | 4:75700001-75800001 | 0.0011 | -9.88 | 397 | MOS;LYN;RPS20 | CHCHD7;RPS20;SDR16C5;MOS;PENK;TMEM68;LOC100626876;TGS1;LYN;LOC106510084;PLAG1 |
| Apulo Calabrese | 5:13400001-13500001 | 0.0042 | -7.89 | 656 | RIC8B;CRY1 | MTERF2;RIC8B;CRY1;RFX4;TMEM263 |
| Apulo Calabrese | 5:13500001-13600001 | 0.0028 | -8.47 | 770 | RFX4;RIC8B | RIC8B;MTERF2;CRY1;LOC110260837;RFX4;TMEM263;LOC110260652 |
| Apulo Calabrese | 5:83500001-83600001 | 0.0040 | -7.97 | 1507 | GAS2L3 | ANO4;NR1H4;SLC17A8;GAS2L3 |
| Apulo Calabrese | 18:38300001-38400001 | 0.0026 | -8.60 | 512 | HERPUD2 | HERPUD2;SEPT7 |
| Basque | 4:37200001-37300001 | 0.0015 | -9.37 | 769 | VPS13B | RGS22;COX6C;VPS13B |
| Basque | 4:73300001-73400001 | 0.0011 | -9.86 | 984 | - | CA8 |
| Basque | 4:73400001-73500001 | 0.0013 | -9.60 | 1005 | - | - |
| Basque | 4:73600001-73700001 | 0.0012 | -9.71 | 954 | - | TOX |
| Basque | 4:73900001-74000001 | 0.0016 | -9.28 | 529 | TOX | TOX |
| Basque | 4:75500001-75600001 | 0.0004 | -11.22 | 294 | LOC100626876;LOC106510084;PENK;SDR16C5 | CHCHD7;RPS20;SDR16C5;MOS;PENK;LOC100626876;LYN;LOC106510084;PLAG1 |
| Basque | 4:80500001-80600001 | 0.0008 | -10.22 | 584 | PRRX1;GORAB | PRRX1;GORAB |
| Basque | 6:19800001-19900001 | 0.0011 | -9.76 | 1933 | CNGB1;ZNF319;MMP15;TEPP;USB1 | MMP15;TEPP;USB1;CNGB1;ZNF319;PRSS54;KATNB1;CCDC113;CSNK2A2;DRC7;CFAP20;KIFC3 |
| Basque | 6:53600001-53700001 | 0.0009 | -10.13 | 357 | CABP5;LOC102165847;LIG1;ELSPBP1;C6H19orf68 | EHD2;CRX;LOC102165847;C6H19orf68;SELENOW;NOP53;GRWD1;LIG1;ELSPBP1;SULT2A1;KCNJ14;BICRA;TMEM143;CYTH2;BSPH1;LMTK3;LOC100622750;CCDC114;SYNGR4;EMP3;GRIN2D;ZNF114;KDELR1;CABP5 |
| Basque | 6:70600001-70700001 | 0.0010 | -9.96 | 683 | PGD;KIF1B | UBE4B;PGD;CASZ1;KIF1B;DFFA;APITD1;CORT;PEX14 |
| Basque | 7:55400001-55500001 | 0.0008 | -10.38 | 329 | AP3S2;ARPIN | AP3S2;TICRR;WDR93;KIF7;ARPIN;ZNF710;PEX11A;ANPEP;IDH2;MESP1;MESP2;PLIN1 |
| Basque | 12:5000001-5100001 | 0.0011 | -9.85 | 1497 | LOC110255264;RHBDF2;LOC100518330;UBE2O | METTL23;SPHK1;CYGB;JMJD6;UBE2O;SRSF2;LOC110255264;QRICH2;RNF157;PRCD;MFSD11;RHBDF2;UBALD2;MXRA7;PRPSAP1;LOC100518330;ST6GALNAC2;ST6GALNAC1 |
| Bísara | 5:82400001-82500001 | 0.0053 | -7.57 | 1492 | GNPTAB;SYCP3 | DRAM1;WASHC3;GNPTAB;CHPT1;MYBPC1;SYCP3 |
| Bísara | 8:12900001-13000001 | 0.0008 | -10.37 | 1067 | LCORL | NCAPG;DCAF16;FAM184B;LCORL |
| Bísara | 8:42600001-42700001 | 0.0053 | -7.57 | 683 | MAP9;LOC100620475 | LOC102162630;LOC100526059;MAP9;TLL1;LOC100620475 |
| Bísara | 8:42700001-42800001 | 0.0058 | -7.43 | 1035 | MAP9 | LOC102162630;LOC100526059;MAP9;TLL1;LOC100620475 |
| Bísara | 8:45500001-45600001 | 0.0039 | -7.99 | 799 | PDGFC | PDGFC |
| Bísara | 8:46000001-46100001 | 0.0048 | -7.70 | 450 | GRIA2;GLRB | GRIA2;PDGFC;GLRB |
| Bísara | 13:133300001-133400001 | 0.0058 | -7.42 | 1018 | PIGX;PAK2 | FBXO45;WDR53;NCBP2;SENP5;NRROS;PIGX;PIGZ;CEP19;MELTF;PAK2 |
| Bísara | 15:96900001-97000001 | 0.0047 | -7.72 | 622 | - | TMEFF2 |
| Bísara | 18:14300001-14400001 | 0.0060 | -7.37 | 1004 | CALD1 | AGBL3;BPGM;WDR91;C18H7orf49;TMEM140;AKR1B1;CALD1 |
| Bísara | 18:32000001-32100001 | 0.0068 | -7.21 | 859 | PPP1R3A | FOXP2;PPP1R3A |
| Bísara | 18:32200001-32300001 | 0.0066 | -7.23 | 727 | - | PPP1R3A |
| Bísara | NW_018085246.1:300001-362670 | 0.0031 | -8.34 | 955 | - | - |
| Black Slavonian | 1:114700001-114800001 | 0.0075 | -7.06 | 462 | TCF12 | CGNL1;TCF12 |
| Black Slavonian | 2:31400001-31500001 | 0.0077 | -7.01 | 836 | - | - |
| Black Slavonian | 2:31500001-31600001 | 0.0067 | -7.23 | 729 | - | - |
| Black Slavonian | 4:42900001-43000001 | 0.0059 | -7.41 | 543 | TMEM67;RBM12B;FAM92A | TMEM67;PDP1;FAM92A;RBM12B |
| Black Slavonian | 4:43100001-43200001 | 0.0073 | -7.10 | 702 | - | TMEM67;RBM12B;FAM92A |
| Black Slavonian | 5:55200001-55300001 | 0.0039 | -8.00 | 548 | - | - |
| Black Slavonian | 5:55300001-55400001 | 0.0077 | -7.01 | 567 | - | - |
| Black Slavonian | 5:55400001-55500001 | 0.0053 | -7.55 | 523 | - | - |
| Black Slavonian | 5:55600001-55700001 | 0.0051 | -7.60 | 270 | - | - |
| Black Slavonian | 6:37200001-37300001 | 0.0066 | -7.24 | 1753 | ITFG1 | NETO2;ITFG1;PHKB |
| Black Slavonian | 13:97500001-97600001 | 0.0077 | -7.03 | 427 | - | VEPH1 |
| Black Slavonian | 15:72000001-72100001 | 0.0069 | -7.18 | 484 | CSRNP3 | CSRNP3;SCN3A;GALNT3;LOC100515171 |
| Casertana | 1:154500001-154600001 | 0.0046 | -7.77 | 508 | - | - |
| Casertana | 1:170300001-170400001 | 0.0055 | -7.50 | 829 | - | - |
| Casertana | 2:142200001-142300001 | 0.0043 | -7.88 | 818 | ANKHD1 | LOC100621895;LOC110259511;TMCO6;ZMAT2;LOC100513976;ANKHD1;LOC100621701;HARS;LOC100738826;SLC4A9;APBB3;SLC35A4;HBEGF;PFDN1;NDUFA2;LOC100521256;CYSTM1;LOC100621803;WDR55;IK;CD14;DND1;LOC102167522;SRA1;EIF4EBP3 |
| Casertana | 3:100200001-100300001 | 0.0056 | -7.49 | 1054 | SLC8A1 | SLC8A1 |
| Casertana | 5:83500001-83600001 | 0.0041 | -7.94 | 1507 | GAS2L3 | ANO4;NR1H4;SLC17A8;GAS2L3 |
| Casertana | 7:61700001-61800001 | 0.0042 | -7.91 | 768 | - | SSTR1;CLEC14A |
| Casertana | 7:61900001-62000001 | 0.0050 | -7.64 | 674 | SSTR1 | SSTR1;CLEC14A |
| Casertana | 7:62000001-62100001 | 0.0048 | -7.70 | 857 | - | SSTR1;TTC6;CLEC14A |
| Casertana | 7:62100001-62200001 | 0.0024 | -8.70 | 813 | - | SSTR1;TTC6 |
| Casertana | 8:59500001-59600001 | 0.0042 | -7.89 | 680 | - | - |
| Casertana | 13:97100001-97200001 | 0.0051 | -7.61 | 596 | VEPH1 | CCNL1;VEPH1;PTX3 |
| Casertana | 13:137700001-137800001 | 0.0053 | -7.57 | 1288 | DIRC2;HSPBAP1 | DTX3L;HSPBAP1;SEMA5B;PARP14;DIRC2;LOC100520273;PARP9 |
| Cinta Senese | 2:72600001-72700001 | 0.0039 | -8.01 | 453 | SLC25A41;GTF2F1;PSPN;TUBB4A;KHSRP;CRB3;SLC25A23;DENND1C | SLC25A41;ACER1;CD70;ACSBG2;LOC100519295;TNFSF14;CLPP;TUBB4A;KHSRP;MLLT1;CRB3;GPR108;TNFSF9;DENND1C;SH2D3A;SLC25A23;ALKBH7;TRIP10;GTF2F1;PSPN;C3 |
| Cinta Senese | 2:77500001-77600001 | 0.0026 | -8.60 | 558 | ELANE;PLPPR3;CFD;AZU1;MED16;PTBP1;PRTN3 | ELANE;TMEM259;ARHGAP45;CDC34;FSTL3;GRIN3B;MED16;MADCAM1;PTBP1;BSG;PRTN3;KISS1R;PRSS57;PLPPR3;R3HDM4;POLR2E;PALM;TPGS1;MISP;ARID3A;SBNO2;CNN2;GZMM;CFD;HCN2;AZU1;ABCA7;GPX4;POLRMT;RNF126;WDR18;FGF22 |
| Cinta Senese | 2:77600001-77700001 | 0.0029 | -8.45 | 317 | PRSS57;FSTL3;PALM;POLRMT;RNF126;FGF22;MISP | ELANE;C2CD4C;TMEM259;THEG;CDC34;FSTL3;GRIN3B;ODF3L2;MED16;MADCAM1;PTBP1;BSG;PRTN3;KISS1R;PRSS57;SHC2;PLPPR3;R3HDM4;PALM;TPGS1;MISP;ARID3A;GZMM;CFD;HCN2;AZU1;POLRMT;RNF126;WDR18;FGF22 |
| Cinta Senese | 2:77700001-77800001 | 0.0038 | -8.06 | 344 | GZMM;HCN2;CDC34;MADCAM1;POLRMT;TPGS1;BSG | ELANE;C2CD4C;THEG;LOC100517809;CDC34;FSTL3;ODF3L2;MED16;MADCAM1;TPGS1;BSG;PRTN3;PRSS57;PLPP2;SHC2;PLPPR3;MIER2;PALM;PTBP1;MISP;GZMM;CFD;HCN2;AZU1;POLRMT;RNF126;FGF22 |
| Cinta Senese | 2:77800001-77900001 | 0.0024 | -8.72 | 413 | THEG;C2CD4C;ODF3L2;SHC2 | C2CD4C;THEG;FLT4;LOC100517809;CDC34;FSTL3;ODF3L2;MADCAM1;TPGS1;BSG;PRSS57;PLPP2;SHC2;MIER2;PALM;MISP;LOC102162368;GZMM;HCN2;POLRMT;RNF126;FGF22 |
| Cinta Senese | 2:78400001-78500001 | 0.0034 | -8.21 | 332 | RASGEF1C | RASGEF1C;TBC1D9B;RNF130;GFPT2;MAPK9;CNOT6 |
| Cinta Senese | 2:78800001-78900001 | 0.0037 | -8.07 | 348 | LTC4S;CANX;MAML1 | TBC1D9B;SQSTM1;RUFY1;MAML1;RNF130;LTC4S;HNRNPH1;MGAT4B;MRNIP;CANX;CBY3 |
| Cinta Senese | 2:78900001-79000001 | 0.0031 | -8.33 | 359 | RUFY1;HNRNPH1;CANX;CBY3 | SQSTM1;MAML1;RUFY1;ADAMTS2;TBC1D9B;LTC4S;HNRNPH1;MGAT4B;MRNIP;CANX;CBY3 |
| Cinta Senese | 3:47300001-47400001 | 0.0037 | -8.10 | 1585 | SH3RF3;EDAR | LOC100511376;CCDC138;SH3RF3;EDAR |
| Cinta Senese | 4:75700001-75800001 | 0.0038 | -8.04 | 397 | MOS;LYN;RPS20 | CHCHD7;RPS20;SDR16C5;MOS;PENK;TMEM68;LOC100626876;TGS1;LYN;LOC106510084;PLAG1 |
| Cinta Senese | 8:45900001-46000001 | 0.0032 | -8.27 | 649 | GLRB | GRIA2;PDGFC;GLRB |
| Cinta Senese | 8:68500001-68600001 | 0.0031 | -8.34 | 649 | NPFFR2 | NPFFR2;GC;ADAMTS3 |
| Gascon | 4:42900001-43000001 | 0.0030 | -8.38 | 543 | TMEM67;RBM12B;FAM92A | TMEM67;PDP1;FAM92A;RBM12B |
| Gascon | 4:43000001-43100001 | 0.0019 | -9.01 | 409 | - | TMEM67;RBM12B;FAM92A |
| Gascon | 6:200001-300001 | 0.0025 | -8.66 | 1184 | TCF25;ZNF276;FANCA;SPIRE2 | MC1R;CENPBD1;DBNDD1;VPS9D1;SPG7;SPATA2L;CDK10;DEF8;CPNE7;LOC100738955;SPATA33;SPIRE2;GAS8;PRDM7;ZNF276;FANCA;RPL13;TCF25;CHMP1A;DPEP1;ANKRD11 |
| Gascon | 6:700001-800001 | 0.0016 | -9.26 | 1525 | ACSF3;CBFA2T3 | TRAPPC2L;CDH15;RNF166;PABPN1L;CDT1;PIEZO1;ACSF3;GALNS;CBFA2T3;CTU2;APRT;SNAI3;LOC110260879;ANKRD11 |
| Gascon | 13:86400001-86500001 | 0.0024 | -8.72 | 411 | PLOD2 | PLOD2;PLSCR4;LOC110256334 |
| Gascon | 13:87200001-87300001 | 0.0021 | -8.89 | 494 | - | - |
| Gascon | 13:87500001-87600001 | 0.0030 | -8.39 | 571 | - | ZIC4;ZIC1 |
| Gascon | 15:22200001-22300001 | 0.0018 | -9.11 | 1116 | - | DPP10 |
| Gascon | 15:23500001-23600001 | 0.0030 | -8.40 | 1385 | CCDC93 | INSIG2;LOC100154782;DDX18;CCDC93 |
| Gascon | 15:96900001-97000001 | 0.0022 | -8.86 | 622 | - | TMEFF2 |
| Gascon | NW_018085052.1:1-30003 | 0.0029 | -8.45 | 28 | - | - |
| Gascon | NW_018085344.1:700001-725547 | 0.0018 | -9.14 | 134 | - | - |
| Krškopolje | 1:160700001-160800001 | 0.0053 | -7.56 | 900 | LOC110261667;MC4R | LOC110261667;MC4R |
| Krškopolje | 1:170200001-170300001 | 0.0064 | -7.28 | 821 | - | - |
| Krškopolje | 1:170300001-170400001 | 0.0055 | -7.51 | 829 | - | - |
| Krškopolje | 1:265400001-265500001 | 0.0058 | -7.43 | 169 | NR6A1 | OLFML2A;ARPC5L;NR6A1;RPL35;ADGRD2;GOLGA1;NR5A1;PSMB7;WDR38 |
| Krškopolje | 4:42900001-43000001 | 0.0055 | -7.51 | 543 | TMEM67;RBM12B;FAM92A | TMEM67;PDP1;FAM92A;RBM12B |
| Krškopolje | 4:43100001-43200001 | 0.0055 | -7.51 | 702 | - | TMEM67;RBM12B;FAM92A |
| Krškopolje | 4:75500001-75600001 | 0.0018 | -9.08 | 294 | LOC100626876;LOC106510084;PENK;SDR16C5 | CHCHD7;RPS20;SDR16C5;MOS;PENK;LOC100626876;LYN;LOC106510084;PLAG1 |
| Krškopolje | 4:75600001-75700001 | 0.0039 | -8.02 | 215 | PLAG1;CHCHD7 | CHCHD7;RPS20;SDR16C5;MOS;PENK;LOC100626876;LYN;LOC106510084;PLAG1 |
| Krškopolje | 4:75700001-75800001 | 0.0027 | -8.55 | 397 | MOS;LYN;RPS20 | CHCHD7;RPS20;SDR16C5;MOS;PENK;TMEM68;LOC100626876;TGS1;LYN;LOC106510084;PLAG1 |
| Krškopolje | 4:75800001-75900001 | 0.0064 | -7.29 | 690 | LYN | XKR4;CHCHD7;RPS20;MOS;TMEM68;TGS1;LYN;PLAG1 |
| Krškopolje | 11:31300001-31400001 | 0.0048 | -7.72 | 1065 | - | - |
| Krškopolje | 16:48500001-48600001 | 0.0029 | -8.43 | 850 | MRPS27;MAP1B | MRPS27;MAP1B;PTCD2;ZNF366 |
| Lithuanian Indigenous Wattle | 1:186400001-186500001 | 0.0038 | -8.05 | 468 | AP5M1;EXOC5 | C1H14orf105;AP5M1;NAA30;SLC35F4;EXOC5 |
| Lithuanian Indigenous Wattle | 1:188500001-188600001 | 0.0030 | -8.39 | 836 | GPR135;DAAM1;JKAMP;L3HYPDH | JKAMP;L3HYPDH;DAAM1;RTN1;CCDC175;GPR135 |
| Lithuanian Indigenous Wattle | 3:80700001-80800001 | 0.0035 | -8.18 | 1004 | KIAA1841;PEX13;PUS10 | C3H2orf74;AHSA2;REL;KIAA1841;PUS10;PAPOLG;USP34;PEX13 |
| Lithuanian Indigenous Wattle | 4:43100001-43200001 | 0.0030 | -8.37 | 702 | - | TMEM67;RBM12B;FAM92A |
| Lithuanian Indigenous Wattle | 7:62100001-62200001 | 0.0038 | -8.03 | 813 | - | SSTR1;TTC6 |
| Lithuanian Indigenous Wattle | 7:62200001-62300001 | 0.0033 | -8.23 | 618 | TTC6 | FOXA1;TTC6 |
| Lithuanian Indigenous Wattle | 9:130300001-130400001 | 0.0027 | -8.53 | 1726 | RPS6KC1 | NSL1;FLVCR1;VASH2;RPS6KC1;TATDN3;ANGEL2 |
| Lithuanian Indigenous Wattle | 13:133300001-133400001 | 0.0040 | -7.95 | 1018 | PIGX;PAK2 | FBXO45;WDR53;NCBP2;SENP5;NRROS;PIGX;PIGZ;CEP19;MELTF;PAK2 |
| Lithuanian Indigenous Wattle | 13:133400001-133500001 | 0.0026 | -8.59 | 868 | PIGX;CEP19;NRROS | FBXO45;SMCO1;UBXN7;WDR53;SENP5;RNF168;NRROS;PIGX;CEP19;PAK2 |
| Lithuanian Indigenous Wattle | 15:37900001-38000001 | 0.0041 | -7.94 | 981 | LOC106506206;LOC106506205;LOC404703;LOC106504227;LOC100525433;LOC110257188 | XKR5;LOC106506206;LOC106506205;DEFB1;LOC110256909;LOC404703;SPAG11B;LOC106504227;LOC100525433;AGPAT5;LOC110257188;LOC110257049;SPAG11 |
| Lithuanian Indigenous Wattle | 15:39000001-39100001 | 0.0028 | -8.46 | 846 | VEGFC | VEGFC;SPCS3 |
| Lithuanian Indigenous Wattle | 18:11400001-11500001 | 0.0033 | -8.24 | 1317 | AKR1D1 | CREB3L2;AKR1D1 |
| Lithuanian White Old Type | 1:214800001-214900001 | 0.0025 | -8.62 | 942 | - | KDM4C |
| Lithuanian White Old Type | 4:75500001-75600001 | 0.0021 | -8.90 | 294 | LOC100626876;LOC106510084;PENK;SDR16C5 | CHCHD7;RPS20;SDR16C5;MOS;PENK;LOC100626876;LYN;LOC106510084;PLAG1 |
| Lithuanian White Old Type | 4:75600001-75700001 | 0.0003 | -11.84 | 215 | PLAG1;CHCHD7 | CHCHD7;RPS20;SDR16C5;MOS;PENK;LOC100626876;LYN;LOC106510084;PLAG1 |
| Lithuanian White Old Type | 4:75700001-75800001 | 0.0021 | -8.87 | 397 | MOS;LYN;RPS20 | CHCHD7;RPS20;SDR16C5;MOS;PENK;TMEM68;LOC100626876;TGS1;LYN;LOC106510084;PLAG1 |
| Lithuanian White Old Type | 4:75800001-75900001 | 0.0007 | -10.39 | 690 | LYN | XKR4;CHCHD7;RPS20;MOS;TMEM68;TGS1;LYN;PLAG1 |
| Lithuanian White Old Type | 6:110100001-110200001 | 0.0027 | -8.54 | 388 | ZNF521 | ZNF521 |
| Lithuanian White Old Type | 8:42600001-42700001 | 0.0028 | -8.48 | 683 | MAP9;LOC100620475 | LOC102162630;LOC100526059;MAP9;TLL1;LOC100620475 |
| Lithuanian White Old Type | 8:45500001-45600001 | 0.0031 | -8.35 | 799 | PDGFC | PDGFC |
| Lithuanian White Old Type | 8:46300001-46400001 | 0.0022 | -8.80 | 968 | - | GRIA2 |
| Lithuanian White Old Type | 9:118300001-118400001 | 0.0027 | -8.53 | 791 | PAPPA2 | PAPPA2;RFWD2 |
| Lithuanian White Old Type | 15:38400001-38500001 | 0.0027 | -8.55 | 702 | GPM6A | WDR17;GPM6A;SPATA4 |
| Lithuanian White Old Type | 15:104600001-104700001 | 0.0025 | -8.66 | 368 | ORC2;NIF3L1 | AOX2;NDUFB3;ORC2;CASP10;CFLAR;PPIL3;NIF3L1;CLK1;BZW1 |
| Majorcan Black | 1:85400001-85500001 | 0.0069 | -7.17 | 567 | - | - |
| Majorcan Black | 1:131300001-131400001 | 0.0056 | -7.48 | 568 | EIF2AK4;GPR176 | BMF;BUB1B;EIF2AK4;SRP14;FSIP1;GPR176 |
| Majorcan Black | 1:136400001-136500001 | 0.0047 | -7.72 | 862 | ARHGAP11A | GREM1;AQR;ACTC1;SCG5;FMN1;ARHGAP11A;GJD2 |
| Majorcan Black | 1:170200001-170300001 | 0.0017 | -9.17 | 821 | - | - |
| Majorcan Black | 1:170300001-170400001 | 0.0036 | -8.11 | 829 | - | - |
| Majorcan Black | 1:210800001-210900001 | 0.0054 | -7.53 | 824 | - | - |
| Majorcan Black | 3:200001-300001 | 0.0069 | -7.18 | 1024 | PDGFA;FAM20C;LOC110259820 | PDGFA;SUN1;LOC106507337;FAM20C;PRKAR1B;DNAAF5;LOC110259820 |
| Majorcan Black | 4:51800001-51900001 | 0.0051 | -7.61 | 1002 | RALYL | RALYL;LOC100156775;LOC100522735 |
| Majorcan Black | 5:82400001-82500001 | 0.0053 | -7.57 | 1492 | GNPTAB;SYCP3 | DRAM1;WASHC3;GNPTAB;CHPT1;MYBPC1;SYCP3 |
| Majorcan Black | 8:12800001-12900001 | 0.0023 | -8.75 | 787 | NCAPG;LCORL | MED28;LAP3;DCAF16;NCAPG;LCORL;FAM184B |
| Majorcan Black | 8:12900001-13000001 | 0.0006 | -10.71 | 1067 | LCORL | NCAPG;DCAF16;FAM184B;LCORL |
| Majorcan Black | 13:40500001-40600001 | 0.0045 | -7.80 | 1144 | C13H3orf67 | FAM107A;C13H3orf67;FAM3D |
| Mora Romagnola | 10:62800001-62900001 | 0.0006 | -10.62 | 1026 | - | - |
| Mora Romagnola | 13:163100001-163200001 | 0.0008 | -10.36 | 445 | EPHA6 | EPHA6 |
| Mora Romagnola | 13:168400001-168500001 | 0.0006 | -10.60 | 712 | - | POU1F1;CHMP2B |
| Mora Romagnola | 15:61100001-61200001 | 0.0006 | -10.74 | 366 | RPRM | RPRM |
| Mora Romagnola | 15:61200001-61300001 | 0.0007 | -10.47 | 422 | - | RPRM;GALNT13 |
| Mora Romagnola | 15:63100001-63200001 | 0.0006 | -10.80 | 205 | - | - |
| Mora Romagnola | 15:63200001-63300001 | 0.0006 | -10.79 | 403 | - | NR4A2 |
| Mora Romagnola | 15:63400001-63500001 | 0.0003 | -11.66 | 359 | NR4A2 | NR4A2;GPD2 |
| Mora Romagnola | 15:63800001-63900001 | 0.0007 | -10.57 | 431 | - | GPD2 |
| Mora Romagnola | 15:64200001-64300001 | 0.0006 | -10.80 | 570 | GALNT5;ERMN | GALNT5;CYTIP;ERMN;ACVR1C |
| Mora Romagnola | 15:73600001-73700001 | 0.0006 | -10.67 | 541 | XIRP2 | B3GALT1;XIRP2 |
| Mora Romagnola | 15:73700001-73800001 | 0.0007 | -10.51 | 333 | B3GALT1;XIRP2 | B3GALT1;XIRP2 |
| Moravka | 1:112600001-112700001 | 0.0086 | -6.87 | 578 | FAM81A;MYO1E | FAM81A;MYO1E;GCNT3 |
| Moravka | 2:73000001-73100001 | 0.0039 | -8.02 | 625 | RANBP3;RFX2 | LONP1;RPL36;C2H19orf70;HSD11B1L;MLLT1;RFX2;CAPS;CATSPERD;LOC100513844;NDUFA11;ACSBG2;DUS3L;PRR22;RANBP3;NRTN |
| Moravka | 2:73100001-73200001 | 0.0056 | -7.49 | 531 | RANBP3;CAPS;LOC100513844;NDUFA11;DUS3L;NRTN | SAFB;LONP1;RPL36;C2H19orf70;HSD11B1L;RFX2;CAPS;CATSPERD;LOC100513844;NDUFA11;ACSBG2;DUS3L;SAFB2;PRR22;RANBP3;NRTN |
| Moravka | 2:73200001-73300001 | 0.0061 | -7.35 | 1024 | LONP1;RPL36;C2H19orf70;HSD11B1L;CATSPERD;DUS3L;PRR22 | SAFB;ZNRF4;RANBP3;RPL36;C2H19orf70;HSD11B1L;RFX2;CAPS;CATSPERD;LONP1;LOC100513844;NDUFA11;DUS3L;SAFB2;PRR22;NRTN |
| Moravka | 2:73300001-73400001 | 0.0052 | -7.59 | 349 | SAFB;SAFB2 | SAFB;ZNRF4;RANBP3;RPL36;C2H19orf70;HSD11B1L;CAPS;CATSPERD;LONP1;LOC100513844;NDUFA11;DUS3L;SAFB2;PRR22;NRTN |
| Moravka | 2:77500001-77600001 | 0.0093 | -6.75 | 558 | ELANE;PLPPR3;CFD;AZU1;MED16;PTBP1;PRTN3 | ELANE;TMEM259;ARHGAP45;CDC34;FSTL3;GRIN3B;MED16;MADCAM1;PTBP1;BSG;PRTN3;KISS1R;PRSS57;PLPPR3;R3HDM4;POLR2E;PALM;TPGS1;MISP;ARID3A;SBNO2;CNN2;GZMM;CFD;HCN2;AZU1;ABCA7;GPX4;POLRMT;RNF126;WDR18;FGF22 |
| Moravka | 3:80600001-80700001 | 0.0060 | -7.39 | 860 | KIAA1841;C3H2orf74 | C3H2orf74;AHSA2;REL;KIAA1841;PUS10;USP34;PEX13 |
| Moravka | 3:80700001-80800001 | 0.0047 | -7.74 | 1004 | KIAA1841;PEX13;PUS10 | C3H2orf74;AHSA2;REL;KIAA1841;PUS10;PAPOLG;USP34;PEX13 |
| Moravka | 9:99600001-99700001 | 0.0077 | -7.02 | 705 | CD36;LOC100511343 | SEMA3C;LOC110255497;LOC106504983;CD36;LOC100511343;GNAT3 |
| Moravka | 9:99700001-99800001 | 0.0088 | -6.83 | 349 | CD36 | LOC106504983;GNAT3;LOC110255497;CD36;LOC100511343 |
| Moravka | 10:35400001-35500001 | 0.0067 | -7.23 | 1061 | - | LOC110255599 |
| Moravka | 16:70100001-70200001 | 0.0088 | -6.83 | 1437 | - | - |
| Nero Siciliano | 1:170100001-170200001 | 0.0148 | -6.08 | 612 | - | FBXO33 |
| Nero Siciliano | 1:241800001-241900001 | 0.0148 | -6.08 | 517 | ERP44;INVS | NR4A3;ERP44;INVS;TEX10;STX17 |
| Nero Siciliano | 4:42900001-43000001 | 0.0055 | -7.52 | 543 | TMEM67;RBM12B;FAM92A | TMEM67;PDP1;FAM92A;RBM12B |
| Nero Siciliano | 4:43000001-43100001 | 0.0123 | -6.34 | 409 | - | TMEM67;RBM12B;FAM92A |
| Nero Siciliano | 4:66400001-66500001 | 0.0139 | -6.17 | 788 | C4H8orf34 | C4H8orf34 |
| Nero Siciliano | 9:118000001-118100001 | 0.0142 | -6.14 | 967 | RFWD2 | RFWD2 |
| Nero Siciliano | 13:163200001-163300001 | 0.0146 | -6.10 | 662 | EPHA6 | EPHA6 |
| Nero Siciliano | 13:163500001-163600001 | 0.0085 | -6.88 | 944 | - | EPHA6 |
| Nero Siciliano | 13:163600001-163700001 | 0.0096 | -6.70 | 1027 | - | EPHA6 |
| Nero Siciliano | 13:172000001-172100001 | 0.0149 | -6.07 | 664 | - | - |
| Nero Siciliano | 16:70100001-70200001 | 0.0100 | -6.64 | 1437 | - | - |
| Nero Siciliano | 18:32300001-32400001 | 0.0124 | -6.33 | 900 | - | - |
| Sarda | 2:70200001-70300001 | 0.0081 | -6.95 | 635 | ACP5 | EPOR;ACP5;CNN1;PLPPR2;ELAVL3;PRKCSH;RGL3;LOC100624806;TSPAN16;ELOF1;CCDC151;RAB3D;TMEM205;CCDC159;SWSAP1;ZNF653;ECSIT |
| Sarda | 2:73000001-73100001 | 0.0083 | -6.91 | 625 | RANBP3;RFX2 | LONP1;RPL36;C2H19orf70;HSD11B1L;MLLT1;RFX2;CAPS;CATSPERD;LOC100513844;NDUFA11;ACSBG2;DUS3L;PRR22;RANBP3;NRTN |
| Sarda | 4:34700001-34800001 | 0.0165 | -5.93 | 523 | UBR5 | ODF1;RRM2B;UBR5;LOC110260477;NCALD |
| Sarda | 4:66900001-67000001 | 0.0109 | -6.52 | 1040 | PREX2 | CPA6;PREX2 |
| Sarda | 4:75600001-75700001 | 0.0136 | -6.20 | 215 | PLAG1;CHCHD7 | CHCHD7;RPS20;SDR16C5;MOS;PENK;LOC100626876;LYN;LOC106510084;PLAG1 |
| Sarda | 4:75700001-75800001 | 0.0020 | -8.97 | 397 | MOS;LYN;RPS20 | CHCHD7;RPS20;SDR16C5;MOS;PENK;TMEM68;LOC100626876;TGS1;LYN;LOC106510084;PLAG1 |
| Sarda | 9:113600001-113700001 | 0.0169 | -5.89 | 1359 | LOC100513809 | LOC100525417;PPFIA4;LOC100511828;MYBPH;LOC100511653;LOC100525599;TMEM183A;LOC100513421;ADORA1;LOC100511466;LOC100513809;CHI3L1;MYOG;LOC100513612 |
| Sarda | 10:21800001-21900001 | 0.0133 | -6.23 | 1301 | - | LOC110255652;PTPRC |
| Sarda | 13:163500001-163600001 | 0.0167 | -5.91 | 944 | - | EPHA6 |
| Sarda | 16:48500001-48600001 | 0.0070 | -7.16 | 850 | MRPS27;MAP1B | MRPS27;MAP1B;PTCD2;ZNF366 |
| Sarda | 16:48600001-48700001 | 0.0137 | -6.19 | 836 | MRPS27;PTCD2 | MRPS27;MAP1B;PTCD2;ZNF366 |
| Sarda | 16:52900001-53000001 | 0.0141 | -6.15 | 741 | RANBP17 | FGF18;NPM1;RANBP17;TLX3 |
| Schwäbisch-Hällisches Schwein | 6:145100001-145200001 | 0.0028 | -8.47 | 1060 | SERBP1 | GADD45A;IL12RB2;C6H1orf141;GNG12;IL23R;LOC100519373;SERBP1 |
| Schwäbisch-Hällisches Schwein | 7:55400001-55500001 | 0.0018 | -9.14 | 329 | AP3S2;ARPIN | AP3S2;TICRR;WDR93;KIF7;ARPIN;ZNF710;PEX11A;ANPEP;IDH2;MESP1;MESP2;PLIN1 |
| Schwäbisch-Hällisches Schwein | 7:62100001-62200001 | 0.0029 | -8.41 | 813 | - | SSTR1;TTC6 |
| Schwäbisch-Hällisches Schwein | 8:12900001-13000001 | 0.0011 | -9.88 | 1067 | LCORL | NCAPG;DCAF16;FAM184B;LCORL |
| Schwäbisch-Hällisches Schwein | 9:113400001-113500001 | 0.0046 | -7.75 | 659 | LOC100511653;LOC100525417;LOC100525599;LOC100511466 | LOC100525417;LOC100511828;NOBOX;LOC100511653;LOC100525599;ARHGEF5;LOC100513421;LOC100511466;LOC100513809;TPK1;LOC100513612 |
| Schwäbisch-Hällisches Schwein | 9:118300001-118400001 | 0.0047 | -7.73 | 791 | PAPPA2 | PAPPA2;RFWD2 |
| Schwäbisch-Hällisches Schwein | 13:142200001-142300001 | 0.0036 | -8.14 | 841 | - | LOC106505794 |
| Schwäbisch-Hällisches Schwein | 13:142300001-142400001 | 0.0035 | -8.15 | 602 | - | - |
| Schwäbisch-Hällisches Schwein | 13:142400001-142500001 | 0.0043 | -7.86 | 832 | - | - |
| Schwäbisch-Hällisches Schwein | 15:72900001-73000001 | 0.0019 | -9.03 | 657 | SCN7A;SCN9A | SCN7A;SCN9A |
| Schwäbisch-Hällisches Schwein | 15:78200001-78300001 | 0.0040 | -7.95 | 434 | DLX2 | HAT1;METAP1D;SLC25A12;DLX2;DLX1 |
| Schwäbisch-Hällisches Schwein | 15:107700001-107800001 | 0.0023 | -8.79 | 978 | - | PARD3B |
| Swallow-Bellied Mangalitsa | 5:52800001-52900001 | 0.0027 | -8.52 | 751 | - | PDE3A |
| Swallow-Bellied Mangalitsa | 5:53100001-53200001 | 0.0032 | -8.28 | 852 | - | - |
| Swallow-Bellied Mangalitsa | 7:55900001-56000001 | 0.0028 | -8.48 | 1056 | PRC1 | UBE2Q2;FBXO22;LOC100625251;NRG4;VPS33B;CIB1;NGRN;GDPGP1;SEMA4B;PRC1 |
| Swallow-Bellied Mangalitsa | 9:99500001-99600001 | 0.0031 | -8.35 | 214 | LOC106504983;LOC110255497 | LOC106504983;SEMA3C;LOC110255497;CD36;LOC100511343 |
| Swallow-Bellied Mangalitsa | 13:86600001-86700001 | 0.0015 | -9.34 | 466 | LOC110256334 | PLOD2;PLSCR4;PLSCR1;LOC110256334 |
| Swallow-Bellied Mangalitsa | 13:86800001-86900001 | 0.0025 | -8.64 | 807 | PLSCR1 | PLSCR1;PLSCR5;LOC110256334 |
| Swallow-Bellied Mangalitsa | 13:87000001-87100001 | 0.0023 | -8.75 | 459 | - | PLSCR5;PLSCR1 |
| Swallow-Bellied Mangalitsa | 13:87200001-87300001 | 0.0025 | -8.63 | 494 | - | - |
| Swallow-Bellied Mangalitsa | 13:87500001-87600001 | 0.0029 | -8.44 | 571 | - | ZIC4;ZIC1 |
| Swallow-Bellied Mangalitsa | 13:87800001-87900001 | 0.0032 | -8.27 | 420 | - | ZIC4;ZIC1 |
| Swallow-Bellied Mangalitsa | 13:88200001-88300001 | 0.0032 | -8.27 | 487 | - | - |
| Swallow-Bellied Mangalitsa | 13:88800001-88900001 | 0.0029 | -8.42 | 343 | - | CPA3;AGTR1;CPB1 |
| Turopolje | 3:71200001-71300001 | 0.0004 | -11.32 | 712 | ZNF638;DYSF | ZNF638;PAIP2B;NAGK;DYSF |
| Turopolje | 8:49500001-49600001 | 0.0005 | -11.07 | 776 | - | - |
| Turopolje | 8:49800001-49900001 | 0.0001 | -13.33 | 606 | - | - |
| Turopolje | 8:50100001-50200001 | 0.0005 | -11.07 | 612 | - | FSTL5 |
| Turopolje | 8:50900001-51000001 | 0.0003 | -11.94 | 956 | FSTL5 | FSTL5 |
| Turopolje | 8:51300001-51400001 | 0.0005 | -10.91 | 566 | - | LOC106504658 |
| Turopolje | 8:56600001-56700001 | 0.0003 | -11.86 | 203 | - | - |
| Turopolje | 8:56800001-56900001 | 0.0005 | -10.88 | 216 | - | - |
| Turopolje | 8:56900001-57000001 | 0.0003 | -11.65 | 532 | - | - |
| Turopolje | 8:59000001-59100001 | 0.0003 | -11.76 | 614 | LOC110261960 | LOC110261960 |
| Turopolje | 13:117200001-117300001 | 0.0005 | -10.90 | 325 | PIK3CA;LOC110256340;KCNMB3 | ZNF639;PIK3CA;LOC110256340;LOC100621400;ZMAT3;KCNMB3;GNB4;MFN1 |
| Turopolje | NW_018085080.1:1-71279 | 0.0004 | -11.18 | 146 | protein furry homolog | protein furry homolog |
| Italian Duroc | 6:36800001-36900001 | 0.0002 | -12.09 | 1625 | - | ABCC12;PHKB |
| Italian Duroc | 9:72500001-72600001 | 0.0004 | -11.27 | 546 | FAM133B | PEX1;FAM133B;RBM48;LOC100519264;ANKIB1;GATAD1 |
| Italian Duroc | 9:81800001-81900001 | 0.0006 | -10.62 | 1036 | TMEM106B | VWDE;TMEM106B |
| Italian Duroc | 13:87100001-87200001 | 0.0003 | -11.62 | 353 | - | PLSCR5 |
| Italian Duroc | 15:72900001-73000001 | 0.0002 | -12.54 | 657 | SCN7A;SCN9A | SCN7A;SCN9A |
| Italian Duroc | 15:73100001-73200001 | 0.0006 | -10.73 | 368 | - | SCN7A;SCN9A |
| Italian Duroc | 15:73300001-73400001 | 0.0004 | -11.18 | 758 | - | - |
| Italian Duroc | 15:73700001-73800001 | 0.0006 | -10.60 | 333 | B3GALT1;XIRP2 | B3GALT1;XIRP2 |
| Italian Duroc | 15:73900001-74000001 | 0.0003 | -11.52 | 627 | B3GALT1 | B3GALT1;XIRP2 |
| Italian Duroc | 15:104900001-105000001 | 0.0003 | -11.68 | 352 | TRAK2;ALS2CR12;CASP8 | C2CD6;TRAK2;NDUFB3;CASP8;CASP10;CFLAR;ALS2CR12;STRADB |
| Italian Duroc | 15:105300001-105400001 | 0.0002 | -12.33 | 279 | ALS2;MPP4 | TMEM237;CDK15;ALS2;C2CD6;MPP4 |
| Italian Duroc | NW_018085331.1:1-49329 | 0.0006 | -10.73 | 113 | - | - |
| Italian Landrace | 1:157700001-157800001 | 0.0012 | -9.71 | 751 | - | LOC100153783;SERPINB10;SERPINB2;LOC100156248;SERPINB7 |
| Italian Landrace | 1:158200001-158300001 | 0.0020 | -8.96 | 551 | KDSR;VPS4B | KDSR;SERPINB11;SERPINB13;SERPINB12;VPS4B;BCL2;LOC110261636;LOC110261637;SERPINB5;LOC110261633 |
| Italian Landrace | 4:75600001-75700001 | 0.0014 | -9.52 | 215 | PLAG1;CHCHD7 | CHCHD7;RPS20;SDR16C5;MOS;PENK;LOC100626876;LYN;LOC106510084;PLAG1 |
| Italian Landrace | 4:75700001-75800001 | 0.0004 | -11.35 | 397 | MOS;LYN;RPS20 | CHCHD7;RPS20;SDR16C5;MOS;PENK;TMEM68;LOC100626876;TGS1;LYN;LOC106510084;PLAG1 |
| Italian Landrace | 4:75800001-75900001 | 0.0009 | -10.04 | 690 | LYN | XKR4;CHCHD7;RPS20;MOS;TMEM68;TGS1;LYN;PLAG1 |
| Italian Landrace | 8:12800001-12900001 | 0.0020 | -8.94 | 787 | NCAPG;LCORL | MED28;LAP3;DCAF16;NCAPG;LCORL;FAM184B |
| Italian Landrace | 8:12900001-13000001 | 0.0001 | -12.79 | 1067 | LCORL | NCAPG;DCAF16;FAM184B;LCORL |
| Italian Landrace | 8:42600001-42700001 | 0.0015 | -9.34 | 683 | MAP9;LOC100620475 | LOC102162630;LOC100526059;MAP9;TLL1;LOC100620475 |
| Italian Landrace | 8:45500001-45600001 | 0.0018 | -9.08 | 799 | PDGFC | PDGFC |
| Italian Landrace | 9:99600001-99700001 | 0.0021 | -8.87 | 705 | CD36;LOC100511343 | SEMA3C;LOC110255497;LOC106504983;CD36;LOC100511343;GNAT3 |
| Italian Landrace | 15:61500001-61600001 | 0.0020 | -8.95 | 562 | GALNT13 | GALNT13 |
| Italian Landrace | 15:104600001-104700001 | 0.0021 | -8.87 | 368 | ORC2;NIF3L1 | AOX2;NDUFB3;ORC2;CASP10;CFLAR;PPIL3;NIF3L1;CLK1;BZW1 |
| Italian Large White | 4:75500001-75600001 | 0.0018 | -9.09 | 294 | LOC100626876;LOC106510084;PENK;SDR16C5 | CHCHD7;RPS20;SDR16C5;MOS;PENK;LOC100626876;LYN;LOC106510084; |
| Italian Large White | 4:75600001-75700001 | 0.0040 | -7.95 | 215 | PLAG1;CHCHD7 | CHCHD7;RPS20;SDR16C5;MOS;PENK;LOC100626876;LYN;LOC106510084;PLAG1 |
| Italian Large White | 4:75700001-75800001 | 0.0015 | -9.40 | 397 | MOS;LYN;RPS20 | CHCHD7;RPS20;SDR16C5;MOS;PENK;TMEM68;LOC100626876;TGS1;LYN;LOC106510084;PLAG1 |
| Italian Large White | 4:75800001-75900001 | 0.0021 | -8.90 | 690 | LYN | XKR4;CHCHD7;RPS20;MOS;TMEM68;TGS1;LYN;PLAG1 |
| Italian Large White | 5:82300001-82400001 | 0.0042 | -7.89 | 1328 | GNPTAB;DRAM1 | DRAM1;WASHC3;GNPTAB;NUP37;CHPT1;MYBPC1;SYCP3 |
| Italian Large White | 5:82400001-82500001 | 0.0051 | -7.63 | 1492 | GNPTAB;SYCP3 | DRAM1;WASHC3;GNPTAB;CHPT1;MYBPC1;SYCP3 |
| Italian Large White | 8:12900001-13000001 | 0.0007 | -10.57 | 1067 | LCORL | NCAPG;DCAF16;FAM184B;LCORL |
| Italian Large White | 8:42600001-42700001 | 0.0055 | -7.51 | 683 | MAP9;LOC100620475 | LOC102162630;LOC100526059;MAP9;TLL1;LOC100620475 |
| Italian Large White | 8:45500001-45600001 | 0.0028 | -8.50 | 799 | PDGFC | PDGFC |
| Italian Large White | 15:61500001-61600001 | 0.0070 | -7.16 | 562 | GALNT13 | GALNT13 |
| Italian Large White | 15:104600001-104700001 | 0.0050 | -7.64 | 368 | ORC2;NIF3L1 | AOX2;NDUFB3;ORC2;CASP10;CFLAR;PPIL3;NIF3L1;CLK1;BZW1 |
| Italian Large White | 15:104800001-104900001 | 0.0037 | -8.09 | 499 | CASP10;CFLAR | ORC2;NDUFB3;CASP8;CASP10;CFLAR;ALS2CR12;NIF3L1;TRAK2;STRADB |
| Wild Boar | 1:49100001-49200001 | 0.0025 | -8.64 | 899 | ADGRB3 | ADGRB3 |
| Wild Boar | 1:64100001-64200001 | 0.0010 | -9.97 | 1144 | KLHL32 | NDUFAF4;KLHL32;GPR63;MMS22L |
| Wild Boar | 1:64200001-64300001 | 0.0015 | -9.37 | 365 | KLHL32 | NDUFAF4;KLHL32;MMS22L |
| Wild Boar | 3:27700001-27800001 | 0.0021 | -8.87 | 843 | - | - |
| Wild Boar | 6:166200001-166300001 | 0.0025 | -8.65 | 1916 | ZSWIM5 | TOE1;LOC110255224;HECTD3;EIF2B3;MUTYH;ZSWIM5;PTCH2;UROD;HPDL;LOC110261240;TESK2 |
| Wild Boar | 7:82100001-82200001 | 0.0020 | -8.94 | 1240 | - | - |
| Wild Boar | 8:113200001-113300001 | 0.0017 | -9.17 | 656 | COL25A1 | RPL34;COL25A1;OSTC;ETNPPL |
| Wild Boar | 10:31000001-31100001 | 0.0022 | -8.86 | 851 | C10H9orf64;KIF27 | KIF27;UBQLN1;HNRNPK;GKAP1;C10H9orf64;RMI1 |
| Wild Boar | 10:39900001-40000001 | 0.0021 | -8.87 | 1046 | WAC;LOC100621751 | WAC;BAMBI;LOC102161970;MPP7;LOC100621751 |
| Wild Boar | 11:18600001-18700001 | 0.0015 | -9.43 | 820 | FNDC3A | CAB39L;FNDC3A;MLNR;CDADC1;LOC106505288 |
| Wild Boar | 15:62900001-63000001 | 0.0015 | -9.38 | 508 | - | - |
| Wild Boar | 16:28300001-28400001 | 0.0022 | -8.85 | 714 | - | NNT;PAIP1 |

**Table S13.** Single breed F_ST_ analysis. The genome windows at the extreme lower end of the distributions (99.95^th^ percentile) are presented.

| **Pig population** | **Genome Windows**  **(SSC:start-end bp)*** | **F_ST_** | **Annotated genes** | **Annotated genes (±200 Kb)** |
| --- | --- | --- | --- | --- |
| Alentejana | 1:71100001-71200001 | 0.375 | HACE1;LIN28B | BVES;POPDC3;HACE1;LIN28B;LOC106508909 |
| Alentejana | 1:90500001-90600001 | 0.402 | FILIP1 | TMEM30A;FILIP1;COX7A2;COL12A1 |
| Alentejana | 1:96500001-96600001* | 0.403 | PIAS2;KATNAL2;LOC110260788 | HDHD2;ST8SIA5;IER3IP1;PIAS2;KATNAL2;LOC110260788;SKOR2 |
| Alentejana | 1:96600001-96700001* | 0.400 | KATNAL2;HDHD2 | HDHD2;IER3IP1;PIAS2;KATNAL2;LOC110260788;SKOR2 |
| Alentejana | 1:160200001-160300001 | 0.389 | - | CDH20 |
| Alentejana | 5:29400001-29500001* | 0.366 | WIF1 | TBC1D30;LOC106510322;WIF1;LEMD3;MSRB3 |
| Alentejana | 5:29700001-29800001 | 0.393 | MSRB3 | WIF1;LEMD3;MSRB3 |
| Alentejana | 5:29900001-30000001 | 0.351 | - | HMGA2;MSRB3 |
| Alentejana | 9:500001-600001 | 0.378 | TMEM9B;ASCL3;AKIP1;LOC110262312 | SCUBE2;ASCL3;RPL27A;LOC110262312;ST5;STK33;DENND5A;TMEM9B;AKIP1;TRIM66 |
| Alentejana | 14:128300001-128400001* | 0.376 | FAM204A | FAM204A;RAB11FIP2 |
| Alentejana | 15:102500001-102600001 | 0.517 | - | PLCL1 |
| Alentejana | 15:102600001-102700001 | 0.621 | - | PLCL1 |
| Apulo-Calabrese | 1:157500001-157600001 | 0.398 | - | - |
| Apulo-Calabrese | 2:92800001-92900001 | 0.432 | - | - |
| Apulo-Calabrese | 2:92900001-93000001 | 0.454 | - | - |
| Apulo-Calabrese | 2:93000001-93100001 | 0.411 | - | - |
| Apulo-Calabrese | 2:93200001-93300001 | 0.433 | - | - |
| Apulo-Calabrese | 2:93300001-93400001 | 0.464 | - | - |
| Apulo-Calabrese | 2:93400001-93500001 | 0.409 | - | - |
| Apulo-Calabrese | 2:93800001-93900001 | 0.449 | - | - |
| Apulo-Calabrese | 2:93900001-94000001 | 0.457 | - | - |
| Apulo-Calabrese | 13:132300001-132400001 | 0.437 | ACAP2 | APOD;XXYLT1;ACAP2;PPP1R2 |
| Apulo-Calabrese | 18:19000001-19100001 | 0.409 | NRF1 | AHCYL2;UBE2H;STRIP2;SMKR1;NRF1 |
| Apulo-Calabrese | 18:19200001-19300001 | 0.406 | SMKR1;STRIP2;AHCYL2 | STRIP2;AHCYL2;SMO;SMKR1;TSPAN33;NRF1 |
| Basque | 2:82600001-82700001 | 0.610 | BTF3;FOXD1 | ARHGEF28;BTF3;ANKRA2;UTP15;FOXD1 |
| Basque | 2:83100001-83200001 | 0.653 | ARHGEF28 | ARHGEF28 |
| Basque | 4:127900001-128000001* | 0.709 | - | PKN2 |
| Basque | 6:117000001-117100001 | 0.767 | CCDC178 | CCDC178 |
| Basque | 6:117200001-117300001 | 0.627 | - | CCDC178;ASXL3 |
| Basque | 6:117300001-117400001 | 0.702 | ASXL3 | CCDC178;ASXL3 |
| Basque | 6:117400001-117500001 | 0.635 | ASXL3 | NOL4;ASXL3 |
| Basque | 6:117500001-117600001 | 0.729 | ASXL3 | NOL4;ASXL3 |
| Basque | 6:118300001-118400001 | 0.638 | DTNA | DTNA |
| Basque | 6:127600001-127700001 | 0.634 | - | KCNG2;PQLC1 |
| Basque | 8:114300001-114400001* | 0.668 | PAPSS1 | PAPSS1;SGMS2 |
| Basque | 8:114600001-114700001 | 0.687 | - | DKK2 |
| Bísara | 5:29300001-29400001 | 0.336 | LOC106510322 | GNS;RASSF3;TBC1D30;WIF1;LOC106510322;LEMD3 |
| Bísara | 5:29400001-29500001 | 0.408 | WIF1 | TBC1D30;LOC106510322;WIF1;LEMD3;MSRB3 |
| Bísara | 5:29500001-29600001 | 0.381 | WIF1;LEMD3 | LOC106510322;WIF1;LEMD3;MSRB3 |
| Bísara | 5:29600001-29700001 | 0.354 | LEMD3;MSRB3 | WIF1;LEMD3;MSRB3 |
| Bísara | 5:29700001-29800001 | 0.407 | MSRB3 | WIF1;LEMD3;MSRB3 |
| Bísara | 8:41700001-41800001 | 0.534 | - | KDR |
| Bísara | 8:41800001-41900001 | 0.368 | KDR | LOC100525350;KDR |
| Bísara | 8:42800001-42900001 | 0.335 | TLL1 | TLL1;MAP9;LOC100620475 |
| Bísara | 8:59700001-59800001 | 0.350 | - | ADGRL3 |
| Bísara | 8:62300001-62400001 | 0.405 | - | TECRL |
| Bísara | 8:66900001-67000001 | 0.417 | STATH;CSN1S2;CSN2;CSN1S1 | LOC110262013;CABS1;LOC110262014;ODAM;LOC100624541;PRR27;CSN1S2;LOC110262119;CSN2;CSN1S1;SULT1E1;CSN3;STATH |
| Bísara | 8:70300001-70400001 | 0.345 | EPGN;EREG | AREG;MTHFD2L;CXCL2;EPGN;EREG |
| Black Slavonian | 6:100001-200001 | 0.559 | MC1R;LOC100738955;DBNDD1;DEF8;CENPBD1;GAS8;TCF25 | MC1R;CENPBD1;DBNDD1;VPS9D1;SPATA2L;CDK10;DEF8;CPNE7;LOC100738955;SPATA33;SPIRE2;GAS8;PRDM7;ZNF276;FANCA;TCF25;CHMP1A;DPEP1 |
| Black Slavonian | 6:200001-300001 | 0.712 | TCF25;ZNF276;FANCA;SPIRE2 | MC1R;CENPBD1;DBNDD1;VPS9D1;SPG7;SPATA2L;CDK10;DEF8;CPNE7;LOC100738955;SPATA33;SPIRE2;GAS8;PRDM7;ZNF276;FANCA;RPL13;TCF25;CHMP1A;DPEP1;ANKRD11 |
| Black Slavonian | 6:300001-400001 | 0.435 | FANCA;CDK10;CPNE7;SPATA33;ZNF276;SPATA2L;CHMP1A;DPEP1;VPS9D1 | MC1R;LOC100738955;DBNDD1;VPS9D1;SPG7;SPATA2L;CDK10;DEF8;CPNE7;CENPBD1;SPATA33;SPIRE2;GAS8;ZNF276;FANCA;RPL13;TCF25;CHMP1A;DPEP1;ANKRD11 |
| Black Slavonian | 6:400001-500001 | 0.490 | ANKRD11;SPG7;CPNE7;RPL13 | CDH15;VPS9D1;SPG7;CDK10;ACSF3;CPNE7;SPATA33;SPIRE2;FANCA;ZNF276;SPATA2L;RPL13;TCF25;CHMP1A;DPEP1;ANKRD11 |
| Black Slavonian | 6:500001-600001 | 0.601 | ANKRD11 | CDH15;FANCA;SPG7;CDK10;ACSF3;CPNE7;SPATA33;CBFA2T3;ZNF276;SPATA2L;RPL13;CHMP1A;ANKRD11;DPEP1;VPS9D1 |
| Black Slavonian | 6:600001-700001 | 0.467 | ACSF3;CDH15;ANKRD11 | TRAPPC2L;CDT1;CDH15;SPG7;GALNS;ACSF3;CPNE7;CBFA2T3;APRT;RPL13;PABPN1L;LOC110260879;ANKRD11 |
| Black Slavonian | 6:700001-800001 | 0.493 | ACSF3;CBFA2T3 | TRAPPC2L;CDH15;RNF166;PABPN1L;CDT1;PIEZO1;ACSF3;GALNS;CBFA2T3;CTU2;APRT;SNAI3;LOC110260879;ANKRD11 |
| Black Slavonian | 6:1300001-1400001* | 0.424 | - | CA5A;BANP;ZFPM1;LOC110260888;ZNF469;LOC110261212 |
| Black Slavonian | 6:1400001-1500001* | 0.420 | CA5A;BANP | CA5A;BANP;LOC110260888;JPH3;ZNF469;KLHDC4 |
| Black Slavonian | 8:44200001-44300001 | 0.427 | - | LOC110262006;LOC100513607;LOC106507778;LOC110261946;LOC100621782;GUCY1A3;LOC110261945;LOC100513415;LOC102163658;LOC102163398 |
| Black Slavonian | 8:44600001-44700001 | 0.417 | ASIC5;GUCY1B3 | ASIC5;GUCY1A3;TDO2;CTSO;GUCY1B3 |
| Black Slavonian | NW_018085176.1:1-72613 | 0.428 | - | - |
| Casertana | 3:70600001-70700001 | 0.422 | - | LOC100521659;EXOC6B |
| Casertana | 7:18700001-18800001 | 0.386 | - | - |
| Casertana | 9:35400001-35500001 | 0.383 | GRIA4 | GRIA4 |
| Casertana | 10:52600001-52700001* | 0.435 | COMMD3 | SPAG6;COMMD3;PIP4K2A;BMI1;DNAJC1 |
| Casertana | 13:77600001-77700001 | 0.392 | STAG1 | SLC35G2;STAG1;NCK1 |
| Casertana | 13:77700001-77800001 | 0.393 | STAG1 | SLC35G2;STAG1;IL20RB;NCK1 |
| Casertana | 13:133300001-133400001 | 0.430 | PIGX;PAK2 | FBXO45;WDR53;NCBP2;SENP5;NRROS;PIGX;PIGZ;CEP19;MELTF;PAK2 |
| Casertana | 13:137000001-137100001 | 0.421 | ADCY5 | ADCY5;SEC22A;HACD2;MYLK |
| Casertana | 13:147400001-147500001 | 0.428 | CD200;LOC100517427 | BTLA;ATG3;CCDC80;LOC100517427;TMPRSS7;SLC35A5;CD200;SLC9C1;C13H3orf52;GCSAM |
| Casertana | 15:105200001-105300001 | 0.385 | TMEM237;C2CD6;MPP4 | C2CD6;TRAK2;ALS2;MPP4;TMEM237;CDK15;STRADB |
| Casertana | 15:105300001-105400001 | 0.415 | ALS2;MPP4 | TMEM237;CDK15;ALS2;C2CD6;MPP4 |
| Casertana | 18:48300001-48400001 | 0.447 | STK31;FAM221A | UPP1;HUS1;FAM221A;LANCL2;STK31;SUN3;C18H7orf57 |
| Cinta Senese | 6:104700001-104800001 | 0.473 | - | LOC110261176 |
| Cinta Senese | 8:43300001-43400001 | 0.427 | - | CPE |
| Cinta Senese | 8:43400001-43500001 | 0.448 | - | CPE |
| Cinta Senese | 8:66700001-66800001 | 0.410 | LOC110262013;LOC100624541 | LOC100515222;LOC100624891;LOC110262013;LOC110262014;LOC100624541;LOC100624700;CSN1S2;CSN2;CSN1S1;SULT1E1;LOC110262116;LOC100624788;STATH |
| Cinta Senese | 11:19100001-19200001 | 0.457 | RCBTB2;RB1 | LPAR6;CYSLTR2;RCBTB2;RB1;ITM2B |
| Cinta Senese | 11:19200001-19300001 | 0.702 | LPAR6;RB1 | LPAR6;CYSLTR2;RCBTB2;RB1;ITM2B |
| Cinta Senese | 11:19300001-19400001 | 0.415 | ITM2B;RB1 | RB1;ITM2B;RCBTB2;MED4;NUDT15;LPAR6 |
| Cinta Senese | 11:53600001-53700001 | 0.445 | - | - |
| Cinta Senese | 11:53700001-53800001 | 0.429 | - | - |
| Cinta Senese | 13:148500001-148600001 | 0.417 | NECTIN3 | LOC102157705;NECTIN3 |
| Cinta Senese | 15:60900001-61000001 | 0.414 | - | RPRM |
| Cinta Senese | 17:20900001-21000001 | 0.466 | - | BTBD3 |
| Gascon | 4:49700001-49800001 | 0.520 | CNBD1 | CNBD1 |
| Gascon | 15:18700001-18800001* | 0.554 | NCKAP5 | NCKAP5 |
| Gascon | 15:19600001-19700001* | 0.518 | GPR39 | GPR39;NCKAP5;LYPD1 |
| Gascon | 15:20300001-20400001 | 0.549 | - | ACTR3 |
| Gascon | 15:20500001-20600001 | 0.534 | - | - |
| Gascon | 15:20700001-20800001 | 0.546 | - | LOC100738548 |
| Gascon | 15:20800001-20900001 | 0.584 | LOC100738548 | LOC100738548 |
| Gascon | 15:20900001-21000001 | 0.555 | - | LOC100738548 |
| Gascon | 15:21400001-21500001 | 0.596 | DPP10 | DPP10 |
| Gascon | 15:21500001-21600001 | 0.611 | DPP10 | DPP10 |
| Gascon | 15:21800001-21900001 | 0.525 | DPP10 | DPP10 |
| Gascon | 15:21900001-22000001 | 0.732 | DPP10 | DPP10 |
| Italian Duroc | 15:53900001-54000001 | 0.740 | WRN | WRN;PURG |
| Italian Duroc | 15:55700001-55800001 | 0.798 | - | TNKS;DUSP4 |
| Italian Duroc | 15:56700001-56800001 | 0.810 | OCA2 | LOC100515176;HERC2;GPR148;CFC1B;OCA2 |
| Italian Duroc | 15:56800001-56900001 | 0.840 | OCA2 | AMER3;GPR148;HERC2;CFC1B;OCA2;LOC100515176 |
| Italian Duroc | 15:57100001-57200001 | 0.757 | LOC102162293 | FAM168B;GPR148;ARHGEF4;AMER3;LOC102162293;CFC1B;LOC100515176 |
| Italian Duroc | 15:57700001-57800001 | 0.772 | - | - |
| Italian Duroc | 15:57800001-57900001 | 0.862 | - | - |
| Italian Duroc | 15:57900001-58000001 | 0.770 | - | - |
| Italian Duroc | 15:58000001-58100001 | 0.821 | - | - |
| Italian Duroc | 15:58100001-58200001 | 0.773 | - | - |
| Italian Duroc | 15:58200001-58300001 | 0.794 | - | - |
| Italian Duroc | 15:58300001-58400001 | 0.737 | - | - |
| Italian Landrace | 11:7000001-7100001 | 0.621 | KATNAL1 | HMGB1;KATNAL1 |
| Italian Landrace | 13:154700001-154800001 | 0.606 | - | - |
| Italian Landrace | 13:157200001-157300001 | 0.605 | NFKBIZ;NXPE3 | ZBTB11;RPL24;NFKBIZ;PCNP;NXPE3;TRMT10C;CEP97 |
| Italian Landrace | 13:162900001-163000001 | 0.612 | EPHA6 | EPHA6 |
| Italian Landrace | 13:163200001-163300001 | 0.610 | EPHA6 | EPHA6 |
| Italian Landrace | 13:163400001-163500001 | 0.600 | EPHA6 | EPHA6 |
| Italian Landrace | 13:163500001-163600001 | 0.602 | - | EPHA6 |
| Italian Landrace | 13:163600001-163700001 | 0.608 | - | EPHA6 |
| Italian Landrace | 13:164300001-164400001 | 0.605 | - | - |
| Italian Landrace | 13:167400001-167500001 | 0.632 | LOC100738961 | LOC100738961 |
| Italian Landrace | 13:172000001-172100001 | 0.611 | - | - |
| Italian Landrace | 13:172100001-172200001 | 0.621 | - | - |
| Italian Large White | 1:143600001-143700001 | 0.328 | - | OTUD7A;KLF13;TRPM1;MTMR10 |
| Italian Large White | 6:42200001-42300001 | 0.323 | LOC110261352;ZNF507 | PDCD5;DPY19L3;LOC110261352;ZNF507 |
| Italian Large White | 6:53300001-53400001 | 0.323 | BICRA;ZNF541 | SELENOW;BSPH1;EHD2;BICRA;CRX;MEIS3;NOP53;C5AR2;SULT2A1;C5AR1;ZNF541;NAPA;SLC8A2;DHX34;ELSPBP1;KPTN |
| Italian Large White | 8:40400001-40500001 | 0.321 | LNX1 | FIP1L1;LNX1 |
| Italian Large White | 10:31000001-31100001 | 0.355 | C10H9orf64;KIF27 | KIF27;UBQLN1;HNRNPK;GKAP1;C10H9orf64;RMI1 |
| Italian Large White | 10:31200001-31300001 | 0.333 | UBQLN1;GKAP1 | KIF27;UBQLN1;IDNK;GKAP1;C10H9orf64;FRMD3 |
| Italian Large White | 10:39900001-40000001 | 0.405 | WAC;LOC100621751 | WAC;BAMBI;LOC102161970;MPP7;LOC100621751 |
| Italian Large White | 10:40000001-40100001 | 0.361 | BAMBI;WAC | BAMBI;LOC102161970;WAC;LOC100621751 |
| Italian Large White | 11:7700001-7800001 | 0.341 | B3GLCT;LOC102159067;HSPH1 | HSPH1;TEX26;B3GLCT;LOC102159067;MEDAG |
| Italian Large White | 11:8100001-8200001 | 0.355 | - | RXFP2;B3GLCT |
| Italian Large White | 13:168400001-168500001 | 0.338 | - | POU1F1;CHMP2B |
| Italian Large White | 15:25800001-25900001 | 0.324 | - | GYPC |
| Krškopolje | 1:74800001-74900001* | 0.339 | - | FOXO3;ARMC2;SESN1 |
| Krškopolje | 3:82600001-82700001 | 0.327 | - | - |
| Krškopolje | 3:83000001-83100001 | 0.413 | - | FANCL |
| Krškopolje | 3:83100001-83200001 | 0.435 | FANCL | FANCL |
| Krškopolje | 3:83200001-83300001 | 0.448 | FANCL | FANCL;VRK2 |
| Krškopolje | 3:86000001-86100001 | 0.374 | CLHC1 | LOC100514282;RTN4;CCDC88A;RPS27A;EML6;MTIF2;CLHC1 |
| Krškopolje | 3:86500001-86600001 | 0.404 | EML6;SPTBN1;LOC110260125 | LOC106508926;EML6;SPTBN1;LOC110260125 |
| Krškopolje | 3:86900001-87000001 | 0.335 | C3H2orf73;LOC106508926;ACYP2 | C3H2orf73;LOC106508926;ACYP2;SPTBN1 |
| Krškopolje | 8:36700001-36800001 | 0.332 | LOC100517408 | GABRA4;LOC100517408;GABRB1 |
| Krškopolje | 8:38700001-38800001* | 0.360 | OCIAD2;OCIAD1;FRYL | OCIAD2;OCIAD1;FRYL;DCUN1D4;CWH43 |
| Krškopolje | 8:47900001-48000001 | 0.326 | - | FNIP2;PPID;RAPGEF2;LOC110262100 |
| Krškopolje | 8:48000001-48100001 | 0.323 | LOC110262100 | FNIP2;RAPGEF2;LOC110262100 |
| Lithuanian Indigenous Wattle | 1:157100001-157200001 | 0.444 | - | - |
| Lithuanian Indigenous Wattle | 1:157200001-157300001 | 0.450 | - | - |
| Lithuanian Indigenous Wattle | 1:157300001-157400001 | 0.433 | - | - |
| Lithuanian Indigenous Wattle | 2:10900001-11000001 | 0.445 | MS4A10;LOC110259710;CCDC86;MS4A15 | PTGDR2;PRPF19;TMEM109;CCDC86;CD6;LOC110259710;MS4A10;SLC15A3;MS4A12;MS4A13;TMEM132A;MS4A15 |
| Lithuanian Indigenous Wattle | 6:104600001-104700001 | 0.405 | LOC110261176 | LOC110261176 |
| Lithuanian Indigenous Wattle | 8:66300001-66400001 | 0.475 | LOC100516628;UGT2B31 | LOC100515222;YTHDC1;LOC100515741;LOC100516628;LOC100624891;LOC110262115;LOC110262116;LOC100623504;LOC100515394;UGT2B31 |
| Lithuanian Indigenous Wattle | 8:67000001-67100001 | 0.433 | PRR27;CSN3;CSN1S2;ODAM | CABS1;LOC110262014;ODAM;CSN3;PRR27;CSN1S2;LOC110262119;CSN2;CSN1S1;SULT1E1;STATH |
| Lithuanian Indigenous Wattle | 12:3000001-3100001 | 0.446 | - | RBFOX3;TIMP2;C1QTNF1;LGALS3BP;CANT1;LOC110255949;CEP295NL;ENGASE |
| Lithuanian Indigenous Wattle | 12:3100001-3200001 | 0.396 | C1QTNF1;RBFOX3;CANT1;ENGASE | RBFOX3;TIMP2;C1QTNF1;LGALS3BP;CYTH1;CANT1;LOC110255949;CEP295NL;USP36;ENGASE |
| Lithuanian Indigenous Wattle | 12:3300001-3400001 | 0.420 | CYTH1;TIMP2;USP36 | DNAH17;RBFOX3;TIMP2;C1QTNF1;LGALS3BP;CYTH1;CANT1;PGS1;LOC110255949;CEP295NL;USP36;ENGASE |
| Lithuanian Indigenous Wattle | 12:3400001-3500001 | 0.406 | CYTH1;DNAH17 | DNAH17;TIMP2;SOCS3;LGALS3BP;CYTH1;CANT1;PGS1;LOC110255949;CEP295NL;USP36 |
| Lithuanian Indigenous Wattle | 13:72100001-72200001 | 0.428 | EEFSEC;RUVBL1 | RPN1;DNAJB8;RUVBL1;KBTBD12;EEFSEC;GATA2;SEC61A1 |
| Lithuanian White Old Type | 4:53400001-53500001 | 0.477 | - | - |
| Lithuanian White Old Type | 6:53100001-53200001 | 0.465 | C5AR2;MEIS3;C5AR1;DHX34 | SLC8A2;BICRA;DHX34;MEIS3;ZC3H4;C5AR2;SAE1;C5AR1;ZNF541;NAPA;BBC3;CCDC9;INAFM1;KPTN |
| Lithuanian White Old Type | 6:53200001-53300001 | 0.532 | SLC8A2;ZNF541;NAPA;KPTN | SELENOW;SLC8A2;BICRA;EHD2;DHX34;MEIS3;NOP53;C5AR2;SAE1;C5AR1;ZNF541;NAPA;BBC3;CCDC9;INAFM1;KPTN |
| Lithuanian White Old Type | 6:53300001-53400001 | 0.529 | BICRA;ZNF541 | SELENOW;BSPH1;EHD2;BICRA;CRX;MEIS3;NOP53;C5AR2;SULT2A1;C5AR1;ZNF541;NAPA;SLC8A2;DHX34;ELSPBP1;KPTN |
| Lithuanian White Old Type | 6:53400001-53500001 | 0.520 | SELENOW;BICRA;EHD2;NOP53 | BSPH1;EHD2;SELENOW;CRX;BICRA;NOP53;C6H19orf68;SULT2A1;ZNF541;LIG1;NAPA;LOC102165847;SLC8A2;CABP5;ELSPBP1;KPTN |
| Lithuanian White Old Type | 6:53700001-53800001 | 0.503 | LOC100622750;CCDC114;SYNGR4;TMEM143;EMP3;ZNF114;KDELR1 | SPHK2;CRX;LOC102165847;FAM83E;C6H19orf68;GRWD1;LIG1;ELSPBP1;SULT2A1;GRIN2D;KCNJ14;CA11;TMEM143;CYTH2;RPL18;SPACA4;BSPH1;LMTK3;LOC100622750;CCDC114;SYNGR4;EMP3;SULT2B1;DBP;ZNF114;KDELR1;CABP5 |
| Lithuanian White Old Type | 6:53800001-53900001 | 0.482 | CYTH2;LMTK3;KCNJ14;GRWD1;GRIN2D;KDELR1 | SPHK2;LOC110261360;RASIP1;LOC102165847;MAMSTR;NTN5;IZUMO1;FAM83E;C6H19orf68;GRWD1;LIG1;FUT1;ELSPBP1;FUT2;GRIN2D;KCNJ14;CA11;TMEM143;FUT2A;CYTH2;RPL18;SPACA4;LMTK3;LOC100622750;CABP5;SYNGR4;EMP3;SULT2B1;DBP;ZNF114;KDELR1;CCDC114;FGF21 |
| Lithuanian White Old Type | 6:54300001-54400001 | 0.494 | LIN7B;PPFIA3;LOC110261361;TRPM4;SNRNP70;HRC | LOC110261362;RPS11;LIN7B;HSD17B14;BAX;CD37;LOC110261361;DHDH;TEAD2;KCNA7;PLEKHA4;PPFIA3;RUVBL2;TULP2;RPL13A;RCN3;TRPM4;SNRNP70;FCGRT;NTF4;LHB;BCAT2;GYS1;ALDH16A1;CCDC155;PTH2;NUCB1;FTL;HRC;GFY;SLC17A7;PPP1R15A;PIH1D1;DKKL1 |
| Lithuanian White Old Type | 6:54500001-54600001 | 0.460 | RPS11;GFY;RCN3;SLC17A7;RPL13A;PIH1D1;CCDC155;ALDH16A1;PTH2;FCGRT | LOC110261362;RPS11;LIN7B;CD37;RRAS;PIH1D1;LOC110261361;TEAD2;NOSIP;PPFIA3;BCL2L12;RPL13A;ALDH16A1;ADM5;SNRNP70;CPT1C;RCN3;TSKS;PRMT1;TRPM4;GFY;AP2A1;SCAF1;CCDC155;PTH2;HRC;IRF3;FCGRT;PRRG2;SLC17A7;PRR12;DKKL1 |
| Lithuanian White Old Type | 8:66300001-66400001 | 0.487 | LOC100516628;UGT2B31 | LOC100515222;YTHDC1;LOC100515741;LOC100516628;LOC100624891;LOC110262115;LOC110262116;LOC100623504;LOC100515394;UGT2B31 |
| Lithuanian White Old Type | 13:86200001-86300001* | 0.468 | - | PLOD2 |
| Lithuanian White Old Type | 13:98700001-98800001 | 0.461 | - | MFSD1;LOC100514494;RARRES1 |
| Majorcan Black | 1:150800001-150900001 | 0.371 | NETO1 | NETO1;CBLN2 |
| Majorcan Black | 1:152400001-152500001 | 0.380 | - | SOCS6;RTTN |
| Majorcan Black | 1:157700001-157800001 | 0.441 | - | LOC100153783;SERPINB10;SERPINB2;LOC100156248;SERPINB7 |
| Majorcan Black | 4:29900001-30000001* | 0.359 | ANGPT1 | ANGPT1 |
| Majorcan Black | 9:99500001-99600001 | 0.391 | LOC106504983;LOC110255497 | LOC106504983;SEMA3C;LOC110255497;CD36;LOC100511343 |
| Majorcan Black | 9:99900001-100000001 | 0.469 | GNAT3 | GNAT3;CD36;GNAI1 |
| Majorcan Black | 15:104800001-104900001 | 0.389 | CASP10;CFLAR | ORC2;NDUFB3;CASP8;CASP10;CFLAR;ALS2CR12;NIF3L1;TRAK2;STRADB |
| Majorcan Black | 15:104900001-105000001 | 0.378 | TRAK2;ALS2CR12;CASP8 | C2CD6;TRAK2;NDUFB3;CASP8;CASP10;CFLAR;ALS2CR12;STRADB |
| Majorcan Black | 15:105200001-105300001 | 0.422 | TMEM237;C2CD6;MPP4 | C2CD6;TRAK2;ALS2;MPP4;TMEM237;CDK15;STRADB |
| Majorcan Black | 15:105300001-105400001 | 0.392 | ALS2;MPP4 | TMEM237;CDK15;ALS2;C2CD6;MPP4 |
| Majorcan Black | NW_018084833.1:500001-600001 | 0.398 | - | - |
| Majorcan Black | NW_018084833.1:600001-700001 | 0.558 | - | - |
| Mora Romagnola | 7:56200001-56300001 | 0.590 | LOC100155488;NRG4 | ETFA;UBE2Q2;TMEM266;FBXO22;LOC106507705;LOC100155488;NRG4 |
| Mora Romagnola | 7:114600001-114700001 | 0.654 | BTBD7;UBR7 | ITPK1;MOAP1;UNC79;LOC100157935;TMEM251;GON7;BTBD7;UBR7 |
| Mora Romagnola | 8:64800001-64900001 | 0.588 | - | - |
| Mora Romagnola | 13:60800001-60900001 | 0.596 | - | LOC100514009;SUMF1;ITPR1 |
| Mora Romagnola | 13:139600001-139700001 | 0.632 | GTF2E1;HGD;RABL3 | GTF2E1;RABL3;NDUFB4;FSTL1;HGD;STXBP5L |
| Mora Romagnola | 13:165800001-165900001 | 0.594 | STX19;ARL13B;NSUN3 | STX19;ARL13B;NSUN3;PROS1 |
| Mora Romagnola | 15:57800001-57900001 | 0.584 | - | - |
| Mora Romagnola | 15:85800001-85900001 | 0.598 | - | CWC22 |
| Mora Romagnola | 15:85900001-86000001 | 0.626 | - | - |
| Mora Romagnola | 15:119300001-119400001 | 0.600 | - | - |
| Mora Romagnola | 15:119400001-119500001 | 0.614 | - | - |
| Mora Romagnola | 17:21000001-21100001 | 0.687 | - | BTBD3 |
| Moravka | 1:91800001-91900001 | 0.308 | - | CD109 |
| Moravka | 1:223900001-224000001* | 0.303 | TRPM3 | KLF9;TRPM3 |
| Moravka | 5:29300001-29400001 | 0.313 | LOC106510322 | GNS;RASSF3;TBC1D30;WIF1;LOC106510322;LEMD3 |
| Moravka | 5:29400001-29500001 | 0.362 | WIF1 | TBC1D30;LOC106510322;WIF1;LEMD3;MSRB3 |
| Moravka | 5:29500001-29600001 | 0.342 | WIF1;LEMD3 | LOC106510322;WIF1;LEMD3;MSRB3 |
| Moravka | 5:30000001-30100001 | 0.316 | - | HMGA2;MSRB3 |
| Moravka | 5:30100001-30200001 | 0.314 | HMGA2 | HMGA2 |
| Moravka | 5:30200001-30300001 | 0.315 | HMGA2 | LLPH;TMBIM4;HMGA2 |
| Moravka | 5:30400001-30500001 | 0.306 | TMBIM4;LLPH | HMGA2;LLPH;HELB;GRIP1;IRAK3;TMBIM4 |
| Moravka | 5:30500001-30600001 | 0.394 | IRAK3;TMBIM4 | HMGA2;LLPH;HELB;GRIP1;IRAK3;TMBIM4 |
| Moravka | 5:30600001-30700001 | 0.334 | GRIP1;IRAK3;HELB | GRIP1;IRAK3;TMBIM4;LLPH;HELB |
| Moravka | 13:179200001-179300001 | 0.305 | LOC100152428 | HSPA13;LOC100152428;LIPI;ROBO2;RBM11;SAMSN1 |
| Nero Siciliano | 1:217800001-217900001 | 0.258 | GLIS3 | GLIS3 |
| Nero Siciliano | 1:245400001-245500001 | 0.247 | SMC2 | SMC2 |
| Nero Siciliano | 4:54900001-55000001 | 0.246 | IMPA1;SLC10A5;ZFAND1 | SNX16;PMP2;SLC10A5;CHMP4C;IMPA1;FABP4;ZFAND1 |
| Nero Siciliano | 8:31000001-31100001 | 0.305 | - | UBE2K;N4BP2;LOC110262080;SMIM14 |
| Nero Siciliano | 8:34800001-34900001 | 0.262 | KCTD8 | KCTD8 |
| Nero Siciliano | 8:49000001-49100001 | 0.269 | - | - |
| Nero Siciliano | 8:49100001-49200001 | 0.246 | - | - |
| Nero Siciliano | 9:71000001-71100001 | 0.248 | CDK14 | MTERF1;CDK14 |
| Nero Siciliano | 9:99400001-99500001 | 0.263 | SEMA3C | LOC106504983;SEMA3C;LOC110255497;CD36;LOC100511343 |
| Nero Siciliano | 9:99500001-99600001 | 0.375 | LOC106504983;LOC110255497 | LOC106504983;SEMA3C;LOC110255497;CD36;LOC100511343 |
| Nero Siciliano | 9:99900001-100000001 | 0.254 | GNAT3 | GNAT3;CD36;GNAI1 |
| Nero Siciliano | 13:170300001-170400001 | 0.300 | CADM2 | CADM2 |
| Sarda | 1:216400001-216500001 | 0.195 | RIC1 | CD274;PLGRKT;ERMP1;RIC1;KIAA2026;MLANA;PDCD1LG2 |
| Sarda | 5:29300001-29400001 | 0.197 | LOC106510322 | GNS;RASSF3;TBC1D30;WIF1;LOC106510322;LEMD3 |
| Sarda | 5:29400001-29500001 | 0.225 | WIF1 | TBC1D30;LOC106510322;WIF1;LEMD3;MSRB3 |
| Sarda | 5:29500001-29600001 | 0.208 | WIF1;LEMD3 | LOC106510322;WIF1;LEMD3;MSRB3 |
| Sarda | 5:30000001-30100001 | 0.191 | - | HMGA2;MSRB3 |
| Sarda | 5:30100001-30200001 | 0.194 | HMGA2 | HMGA2 |
| Sarda | 5:30200001-30300001 | 0.190 | HMGA2 | LLPH;TMBIM4;HMGA2 |
| Sarda | 6:104300001-104400001 | 0.208 | METTL4 | METTL4;LOC110261176 |
| Sarda | 8:66900001-67000001 | 0.191 | STATH;CSN1S2;CSN2;CSN1S1 | LOC110262013;CABS1;LOC110262014;ODAM;LOC100624541;PRR27;CSN1S2;LOC110262119;CSN2;CSN1S1;SULT1E1;CSN3;STATH |
| Sarda | 8:84500001-84600001* | 0.202 | USP38 | USP38;GAB1 |
| Sarda | 13:106500001-106600001 | 0.202 | PDCD10;SERPINI1 | PDCD10;WDR49;SERPINI2;SERPINI1 |
| Sarda | NW_018084901.1:200001-300001 | 0.229 | - | - |
| Schwäbisch-Hällisches Schwein | 2:54300001-54400001 | 0.420 | LOC110259743;LOC100623502 | LOC100514097;LOC100518479;LOC110259743;LOC110259747;LOC110259744;LOC100518655;LOC100517216;LOC110259594;LOC100518301;LOC100623502 |
| Schwäbisch-Hällisches Schwein | 4:28600001-28700001 | 0.474 | - | TRHR;TMEM74 |
| Schwäbisch-Hällisches Schwein | 8:43700001-43800001 | 0.451 | MSMO1;KLHL2 | MSMO1;TMEM192;KLHL2;CPE |
| Schwäbisch-Hällisches Schwein | 8:43800001-43900001 | 0.442 | KLHL2 | MSMO1;LOC110262006;CPE;LOC110261946;TMEM192;LOC110261945;KLHL2;LOC102163658;LOC102163398 |
| Schwäbisch-Hällisches Schwein | 8:46600001-46700001 | 0.431 | - | - |
| Schwäbisch-Hällisches Schwein | 8:100900001-101000001 | 0.446 | SPATA5 | SPRY1;SPATA5 |
| Schwäbisch-Hällisches Schwein | 8:101000001-101100001 | 0.533 | SPATA5 | SPRY1;NUDT6;SPATA5;FGF2 |
| Schwäbisch-Hällisches Schwein | 8:101200001-101300001 | 0.476 | NUDT6;SPATA5;FGF2 | LOC100622849;BBS12;NUDT6;SPATA5;FGF2 |
| Schwäbisch-Hällisches Schwein | 8:101300001-101400001 | 0.476 | BBS12;FGF2;NUDT6 | NUDT6;IL21;BBS12;LOC100622849;FGF2;SPATA5 |
| Schwäbisch-Hällisches Schwein | 8:101400001-101500001 | 0.466 | LOC100622849;BBS12 | NUDT6;IL21;BBS12;IL2;LOC100622849;ADAD1;FGF2;SPATA5 |
| Schwäbisch-Hällisches Schwein | 15:89000001-89100001 | 0.479 | - | - |
| Schwäbisch-Hällisches Schwein | 15:97300001-97400001 | 0.435 | - | - |
| Swallow-Bellied Mangalitsa | 1:266100001-266200001 | 0.430 | MAPKAP1 | GAPVD1;HSPA5;LOC106507123;RABEPK;MAPKAP1;PBX3 |
| Swallow-Bellied Mangalitsa | 2:53200001-53300001 | 0.461 | LOC100623613;LOC100623804;LOC100623714 | LOC100520723;LOC100623406;LOC100514828;LOC110259584;LOC100623897;LOC100623804;LOC100515195;LOC110259736;LOC110259737;LOC100623714;LOC100623613;LOC100624358 |
| Swallow-Bellied Mangalitsa | 2:53300001-53400001* | 0.549 | LOC100515195;LOC100623897 | LOC100623406;LOC100623024;LOC100514828;LOC100622639;LOC110259584;LOC100623897;LOC100623804;LOC100515195;LOC110259736;LOC110259737;LOC100623714;LOC100623613 |
| Swallow-Bellied Mangalitsa | 2:53400001-53500001* | 0.453 | LOC100514828 | LOC100623024;LOC100514828;LOC100622639;LOC100623897;LOC100623804;LOC100515195;LOC100623613;LOC100623714;LOC100622735;LOC106508617 |
| Swallow-Bellied Mangalitsa | 4:74400001-74500001 | 0.452 | UBXN2B | FAM110B;NSMAF;CYP7A1;UBXN2B;SDCBP |
| Swallow-Bellied Mangalitsa | 5:30000001-30100001 | 0.430 | - | HMGA2;MSRB3 |
| Swallow-Bellied Mangalitsa | 5:30500001-30600001 | 0.474 | IRAK3;TMBIM4 | HMGA2;LLPH;HELB;GRIP1;IRAK3;TMBIM4 |
| Swallow-Bellied Mangalitsa | 5:61100001-61200001 | 0.508 | SMIM10L1;TAS2R42;LOC100154902 | SL44-1;LOC106507526;TAS2R8;TAS2R9;STYK1;TAS2R7;LOC100522675;LOC110260739;SMIM10L1;TAS2R42;LOC100154902;TP23;LOC100522490;LOC110260741;YBX3 |
| Swallow-Bellied Mangalitsa | 7:55200001-55300001 | 0.455 | PEX11A;KIF7;WDR93;TICRR;PLIN1 | AP3S2;TICRR;RHCG;KIF7;ARPIN;PEX11A;WDR93;ANPEP;MESP1;MESP2;LOC106510141;PLIN1 |
| Swallow-Bellied Mangalitsa | 7:55300001-55400001 | 0.465 | WDR93;MESP1;MESP2;AP3S2;ANPEP | AP3S2;TICRR;WDR93;KIF7;ARPIN;ZNF710;PEX11A;ANPEP;MESP1;MESP2;PLIN1 |
| Swallow-Bellied Mangalitsa | 7:98000001-98100001 | 0.437 | DLST;YLPM1;PROX2 | PGF;LTBP2;RPS6KL1;DLST;FCF1;EIF2B2;ZC2HC1C;NEK9;YLPM1;AREL1;PROX2;ACYP1 |
| Swallow-Bellied Mangalitsa | 8:46600001-46700001 | 0.498 | - | - |
| Turopolje | 3:70200001-70300001 | 0.702 | EXOC6B | EXOC6B |
| Turopolje | 8:43800001-43900001 | 0.696 | KLHL2 | MSMO1;LOC110262006;CPE;LOC110261946;TMEM192;LOC110261945;KLHL2;LOC102163658;LOC102163398 |
| Turopolje | 8:46400001-46500001 | 0.713 | - | GRIA2 |
| Turopolje | 8:46600001-46700001 | 0.720 | - | - |
| Turopolje | 13:105600001-105700001 | 0.703 | - | - |
| Turopolje | 13:105700001-105800001 | 0.674 | - | - |
| Turopolje | 15:59100001-59200001 | 0.654 | HS6ST1;UGGT1 | HS6ST1;SAP130;UGGT1 |
| Turopolje | NW_018085004.1:600001-700001 | 0.707 | - | - |
| Turopolje | NW_018085004.1:700001-800001 | 0.661 | - | - |
| Turopolje | NW_018085200.1:1-100001 | 0.695 | - | - |
| Turopolje | NW_018085200.1:100001-200001 | 0.697 | - | - |
| Turopolje | NW_018085293.1:1-100001 | 0.653 | - | - |
| Wild Boar | 1:265400001-265500001 | 0.644 | NR6A1 | OLFML2A;ARPC5L;NR6A1;RPL35;ADGRD2;GOLGA1;NR5A1;PSMB7;WDR38 |
| Wild Boar | 1:265500001-265600001 | 0.577 | OLFML2A;NR6A1 | SCAI;OLFML2A;ARPC5L;NR6A1;RPL35;GOLGA1;NR5A1;WDR38 |
| Wild Boar | 6:161200001-161300001 | 0.601 | FAF1 | FAF1;CDKN2C |
| Wild Boar | 7:62000001-62100001 | 0.786 | - | SSTR1;TTC6;CLEC14A |
| Wild Boar | 7:62100001-62200001 | 0.770 | - | SSTR1;TTC6 |
| Wild Boar | 7:62400001-62500001 | 0.606 | FOXA1;TTC6 | FOXA1;MIPOL1;TTC6 |
| Wild Boar | 7:77800001-77900001 | 0.594 | SUPT16H;RPGRIP1 | ZNF219;LOC100626020;RNASE13;SUPT16H;CHD8;METTL3;TMEM253;LOC110261607;RAB2B;TOX4;SALL2;RPGRIP1;LOC106504487;HNRNPC;ARHGEF40 |
| Wild Boar | 13:75600001-75700001 | 0.580 | CEP63;ANAPC13 | CEP63;KY;AMOTL2;ANAPC13 |
| Wild Boar | 13:88600001-88700001 | 0.577 | - | - |
| Wild Boar | 15:23900001-24000001 | 0.603 | - | - |
| Wild Boar | 15:91900001-92000001 | 0.595 | - | ZSWIM2;FAM171B;ITGAV |
| Wild Boar | 15:97300001-97400001 | 0.577 | - | - |

***** Genomes windows overlapping or in proximity (< 500 kb) markers carrying signals of selective sweep, as reported by Muñoz et al. (2019).

**Table S14.** Comparative F_ST_ analysis of breed groups. The genome windows at the extreme lower end of the distributions (99.95^th^ percentile) are presented.

| **Comparison** | **Genome Windows (SSC:start-end bp)** | **FST** | **No. of SNPs** | **Annotated genes** | **Annotated genes (±200 Kb)** |
| --- | --- | --- | --- | --- | --- |
| Belted vs. all others | 1:96200001-96300001 | 0.126 | 1070 | LOXHD1;ST8SIA5 | LOXHD1;PIAS2;ST8SIA5;RNF165 |
| Belted vs. all others | 7:55200001-55300001 | 0.131 | 514 | PEX11A;KIF7;WDR93;TICRR;PLIN1 | AP3S2;TICRR;RHCG;KIF7;ARPIN;PEX11A;WDR93;ANPEP;MESP1;MESP2;LOC106510141;PLIN1 |
| Belted vs. all others | 8:41500001-41600001 | 0.130 | 86 | - | KIT |
| Belted vs all others | 8:41600001-41700001 | 0.156 | 42 | - | KDR;KIT |
| Belted vs all others | 8:41700001-41800001 | 0.148 | 179 | - | KDR |
| Belted vs all others | 8:48400001-48500001 | 0.138 | 650 | - | RAPGEF2 |
| Belted vs all others | 8:48500001-48600001 | 0.207 | 802 | - | - |
| Belted vs all others | 11:19200001-19300001 | 0.145 | 720 | LPAR6;RB1 | LPAR6;CYSLTR2;RCBTB2;RB1;ITM2B |
| Belted vs all others | 12:28200001-28300001 | 0.137 | 962 | LOC110256025;CA10 | LOC110256025;CA10 |
| Belted vs all others | 15:48400001-48500001 | 0.123 | 524 | ADRB3;EIF4EBP1;ASH2L | RAB11FIP1;STAR;ERLIN2;BAG4;ASH2L;ZNF703;DDHD2;LSM1;ADRB3;EIF4EBP1;GOT1L1;ADGRA2;BRF2;PLPP5;NSD3;PLPBP |
| Belted vs all others | 15:59500001-59600001 | 0.161 | 365 | WDR33 | GPR17;SFT2D3;LIMS2;POLR2D;AMMECR1L;WDR33;SAP130;MYO7B |
| Belted vs all others | 15:89000001-89100001 | 0.125 | 829 | - | - |
| Belted vs solid | 1:74200001-74300001 | 0.175 | 432 | NR2E1 | SNX3;OSTM1;AFG1L;NR2E1;SEC63 |
| Belted vs solid | 1:74300001-74400001 | 0.175 | 422 | SNX3;AFG1L | SNX3;OSTM1;AFG1L;NR2E1 |
| Belted vs solid | 1:74400001-74500001 | 0.168 | 543 | AFG1L | SNX3;FOXO3;AFG1L;NR2E1 |
| Belted vs solid | 1:96200001-96300001 | 0.151 | 1057 | LOXHD1;ST8SIA5 | LOXHD1;PIAS2;ST8SIA5;RNF165 |
| Belted vs solid | 8:41500001-41600001 | 0.172 | 86 | - | KIT |
| Belted vs solid | 8:41600001-41700001 | 0.235 | 42 | - | KDR;KIT |
| Belted vs solid | 8:41700001-41800001 | 0.170 | 179 | - | KDR |
| Belted vs solid | 9:107100001-107200001 | 0.150 | 798 | COG5 | BCAP29;COG5;HBP1;PRKAR2B;SLC26A4;GPR22;DUS4L |
| Belted vs solid | 11:19200001-19300001 | 0.162 | 710 | LPAR6;RB1 | LPAR6;CYSLTR2;RCBTB2;RB1;ITM2B |
| Belted vs solid | 12:28200001-28300001 | 0.156 | 951 | LOC110256025;CA10 | LOC110256025;CA10 |
| Belted vs solid | 15:59500001-59600001 | 0.176 | 361 | WDR33 | GPR17;SFT2D3;LIMS2;POLR2D;AMMECR1L;WDR33;SAP130;MYO7B |
| Belted vs solid | 18:37700001-37800001 | 0.148 | 920 | KIAA0895;ANLN | KIAA0895;AOAH;EEPD1;ANLN |
| Belted vs solid black | 1:266200001-266300001 | 0.169 | 715 | MAPKAP1 | MAPKAP1;GAPVD1;LOC106507123;PBX3 |
| Belted vs solid black | 7:55200001-55300001 | 0.161 | 503 | PEX11A;KIF7;WDR93;TICRR;PLIN1 | AP3S2;TICRR;RHCG;KIF7;ARPIN;PEX11A;WDR93;ANPEP;MESP1;MESP2;LOC106510141;PLIN1 |
| Belted vs solid black | 8:41500001-41600001 | 0.174 | 86 | - | KIT |
| Belted vs solid black | 8:41600001-41700001 | 0.261 | 42 | - | KDR;KIT |
| Belted vs solid black | 9:39500001-39600001 | 0.147 | 1050 | PPP2R1B;ALG9 | PPP2R1B;ALG9;HSPB2;C9H11orf52;SIK2;LAYN;C9H11orf88;CRYAB;FDXACB1;C9H11orf57;PIH1D2;DLAT;C9H11orf1;DIXDC1;SDHD;TIMM8B |
| Belted vs solid black | 11:19200001-19300001 | 0.164 | 705 | LPAR6;RB1 | LPAR6;CYSLTR2;RCBTB2;RB1;ITM2B |
| Belted vs solid black | 12:16100001-16200001 | 0.151 | 801 | MRC2;TLK2 | MARCH10;MRC2;TLK2;METTL2A;LOC110256160;LOC100516640;EFCAB3 |
| Belted vs solid black | 12:16300001-16400001 | 0.146 | 1221 | LOC100516640;METTL2A;EFCAB3 | LOC100624995;MRC2;TLK2;METTL2A;LOC110256160;LOC110255897;LOC100516640;LOC106504254;EFCAB3 |
| Belted vs solid black | 13:129800001-129900001 | 0.166 | 726 | FGF12;MB21D2 | FGF12;MB21D2 |
| Belted vs solid black | 15:59500001-59600001 | 0.172 | 355 | WDR33 | GPR17;SFT2D3;LIMS2;POLR2D;AMMECR1L;WDR33;SAP130;MYO7B |
| Belted vs solid black | 16:48400001-48500001 | 0.147 | 880 | MAP1B | MRPS27;MAP1B;PTCD2 |
| Belted vs solid black | 18:18200001-18300001 | 0.152 | 448 | TSGA13;COPG2 | KLF14;CPA1;CEP41;MEST;TSGA13;COPG2;CPA5 |
| Belted vs solid white | 4:100800001-100900001 | 0.441 | 791 | LOC100157002;LOC100624559 | LOC100157002;LOC100624559;NOTCH2 |
| Belted vs solid white | 6:53200001-53300001 | 0.390 | 255 | SLC8A2;ZNF541;NAPA;KPTN | SELENOW;SLC8A2;BICRA;EHD2;DHX34;MEIS3;NOP53;C5AR2;SAE1;C5AR1;ZNF541;NAPA;BBC3;CCDC9;INAFM1;KPTN |
| Belted vs solid white | 6:53300001-53400001 | 0.404 | 321 | BICRA;ZNF541 | SELENOW;BSPH1;EHD2;BICRA;CRX;MEIS3;NOP53;C5AR2;SULT2A1;C5AR1;ZNF541;NAPA;SLC8A2;DHX34;ELSPBP1;KPTN |
| Belted vs solid white | 7:72500001-72600001 | 0.360 | 417 | - | - |
| Belted vs solid white | 7:72900001-73000001 | 0.389 | 419 | NOVA1 | NOVA1 |
| Belted vs solid white | 7:73300001-73400001 | 0.368 | 581 | - | - |
| Belted vs solid white | 7:73700001-73800001 | 0.368 | 591 | - | - |
| Belted vs solid white | 8:48500001-48600001 | 0.547 | 572 | - | - |
| Belted vs solid white | 10:39900001-40000001 | 0.373 | 1003 | WAC;LOC100621751 | WAC;BAMBI;LOC102161970;MPP7;LOC100621751 |
| Belted vs solid white | 11:7700001-7800001 | 0.377 | 590 | B3GLCT;LOC102159067;HSPH1 | HSPH1;TEX26;B3GLCT;LOC102159067;MEDAG |
| Belted vs solid white | 15:106000001-106100001 | 0.361 | 387 | BMPR2 | NOP58;SUMO1;BMPR2;ICA1L;FAM117B;KIAA2012 |
| Belted vs spotted | 1:112400001-112500001 | 0.216 | 689 | GCNT3 | FAM81A;MYO1E;GTF2A2;BNIP2;GCNT3 |
| Belted vs spotted | 1:264500001-264600001 | 0.208 | 539 | DENND1A | DENND1A;CRB2 |
| Belted vs spotted | 5:30100001-30200001 | 0.224 | 352 | HMGA2 | HMGA2 |
| Belted vs spotted | 6:126900001-127000001 | 0.212 | 1349 | - | - |
| Belted vs spotted | 8:41600001-41700001 | 0.229 | 30 | - | KDR;KIT |
| Belted vs spotted | 8:41700001-41800001 | 0.212 | 167 | - | KDR |
| Belted vs spotted | 8:48500001-48600001 | 0.301 | 570 | - | - |
| Belted vs spotted | 8:66700001-66800001 | 0.336 | 621 | LOC110262013;LOC100624541 | LOC100515222;LOC100624891;LOC110262013;LOC110262014;LOC100624541;LOC100624700;CSN1S2;CSN2;CSN1S1;SULT1E1;LOC110262116;LOC100624788;STATH |
| Belted vs spotted | 8:110700001-110800001 | 0.215 | 757 | - | C8H4orf32 |
| Belted vs spotted | 10:29700001-29800001 | 0.210 | 718 | - | AGTPBP1 |
| Belted vs spotted | 14:63800001-63900001 | 0.221 | 917 | ANK3 | ANK3;LOC110256822 |
| Belted vs spotted | 14:119700001-119800001 | 0.235 | 902 | - | - |
| Red vs all others | 15:53800001-53900001 | 0.510 | 373 | - | NRG1;WRN;PURG |
| Red vs all others | 15:53900001-54000001 | 0.518 | 498 | WRN | WRN;PURG |
| Red vs all others | 15:55700001-55800001 | 0.561 | 368 | - | TNKS;DUSP4 |
| Red vs all others | 15:56300001-56400001 | 0.466 | 1225 | - | HERC2;MFHAS1;ERI1 |
| Red vs all others | 15:56700001-56800001 | 0.623 | 737 | OCA2 | LOC100515176;HERC2;GPR148;CFC1B;OCA2 |
| Red vs all others | 15:56800001-56900001 | 0.667 | 709 | OCA2 | AMER3;GPR148;HERC2;CFC1B;OCA2;LOC100515176 |
| Red vs all others | 15:57700001-57800001 | 0.571 | 995 | - | - |
| Red vs all others | 15:57800001-57900001 | 0.656 | 664 | - | - |
| Red vs all others | 15:57900001-58000001 | 0.538 | 798 | - | - |
| Red vs all others | 15:58000001-58100001 | 0.646 | 964 | - | - |
| Red vs all others | 15:58100001-58200001 | 0.540 | 512 | - | - |
| Red vs all others | 15:58200001-58300001 | 0.554 | 835 | - | - |
| Red vs solid white | 1:216400001-216500001 | 0.728 | 648 | RIC1 | CD274;PLGRKT;ERMP1;RIC1;KIAA2026;MLANA;PDCD1LG2 |
| Red vs solid white | 15:55700001-55800001 | 0.667 | 317 | - | TNKS;DUSP4 |
| Red vs solid white | 15:56100001-56200001 | 0.741 | 497 | ERI1 | PPP1R3B;MFHAS1;ERI1 |
| Red vs solid white | 15:56700001-56800001 | 0.708 | 674 | OCA2 | LOC100515176;HERC2;GPR148;CFC1B;OCA2 |
| Red vs solid white | 15:56800001-56900001 | 0.730 | 671 | OCA2 | AMER3;GPR148;HERC2;CFC1B;OCA2;LOC100515176 |
| Red vs solid white | 15:57100001-57200001 | 0.708 | 331 | LOC102162293 | FAM168B;GPR148;ARHGEF4;AMER3;LOC102162293;CFC1B;LOC100515176 |
| Red vs solid white | 15:57800001-57900001 | 0.790 | 574 | - | - |
| Red vs solid white | 15:57900001-58000001 | 0.764 | 619 | - | - |
| Red vs solid white | 15:58000001-58100001 | 0.764 | 848 | - | - |
| Red vs solid white | 15:58100001-58200001 | 0.714 | 407 | - | - |
| Red vs solid white | 15:58200001-58300001 | 0.752 | 649 | - | - |
| Red vs solid white | 15:58300001-58400001 | 0.699 | 334 | - | - |
| Small vs large sized | 8:13000001-13100001 | 0.179 | 866 | - | NCAPG;LCORL |
| Small vs large sized | 10:39900001-40000001 | 0.207 | 1006 | WAC;LOC100621751 | WAC;BAMBI;LOC102161970;MPP7;LOC100621751 |
| Small vs large sized | 13:129800001-129900001 | 0.184 | 698 | FGF12;MB21D2 | FGF12;MB21D2 |
| Small vs large sized | 13:161700001-161800001 | 0.185 | 1188 | LOC110256355;LOC110256354;LOC100623107 | LOC100514797;LOC110256355;LOC110256289;LOC100514252;LOC100623293;LOC110256354;LOC110256356;LOC100623107;LOC110256353 |
| Small vs large sized | 13:166200001-166300001 | 0.180 | 545 | - | - |
| Small vs large sized | 15:9300001-9400001 | 0.170 | 505 | - | - |
| Small vs large sized | 15:77500001-77600001 | 0.173 | 807 | METTL8;DCAF17 | METTL8;DCAF17;TLK1;CYBRD1 |
| Small vs large sized | 15:104800001-104900001 | 0.170 | 222 | CASP10;CFLAR | ORC2;NDUFB3;CASP8;CASP10;CFLAR;ALS2CR12;NIF3L1;TRAK2;STRADB |
| Small vs large sized | 15:104900001-105000001 | 0.205 | 324 | TRAK2;ALS2CR12;CASP8 | C2CD6;TRAK2;NDUFB3;CASP8;CASP10;CFLAR;ALS2CR12;STRADB |
| Small vs large sized | 15:105000001-105100001 | 0.171 | 88 | TRAK2;STRADB | C2CD6;TRAK2;CASP8;MPP4;CASP10;CFLAR;ALS2CR12;TMEM237;STRADB |
| Small vs large sized | 15:105200001-105300001 | 0.312 | 213 | TMEM237;C2CD6;MPP4 | C2CD6;TRAK2;ALS2;MPP4;TMEM237;CDK15;STRADB |
| Small vs large sized | 15:105300001-105400001 | 0.240 | 210 | ALS2;MPP4 | TMEM237;CDK15;ALS2;C2CD6;MPP4 |
| Middle vs large sized | 1:10000001-10100001 | 0.191 | 1470 | ARID1B | ARID1B |
| Middle vs large sized | 1:10400001-10500001 | 0.146 | 1442 | - | LOC110259923;ARID1B |
| Middle vs large sized | 1:27400001-27500001 | 0.139 | 1602 | MAP3K5 | SLC35D3;MAP7;PEX7;MAP3K5 |
| Middle vs large sized | 1:173400001-173500001 | 0.151 | 997 | - | - |
| Middle vs large sized | 1:173700001-173800001 | 0.159 | 810 | - | - |
| Middle vs large sized | 2:41900001-42000001 | 0.157 | 933 | PIK3C2A | RPS13;KCNJ11;PIK3C2A;PLEKHA7;ABCC8;NUCB2 |
| Middle vs large sized | 2:42000001-42100001 | 0.136 | 1230 | RPS13;PIK3C2A;PLEKHA7 | RPS13;PIK3C2A;PLEKHA7;NUCB2 |
| Middle vs large sized | 8:101000001-101100001 | 0.144 | 770 | SPATA5 | SPRY1;NUDT6;SPATA5;FGF2 |
| Middle vs large sized | 15:104700001-104800001 | 0.142 | 136 | CFLAR;NDUFB3 | NDUFB3;ORC2;CASP8;CASP10;CFLAR;ALS2CR12;PPIL3;NIF3L1;TRAK2;CLK1;BZW1 |
| Middle vs large sized | 15:104900001-105000001 | 0.175 | 339 | TRAK2;ALS2CR12;CASP8 | C2CD6;TRAK2;NDUFB3;CASP8;CASP10;CFLAR;ALS2CR12;STRADB |
| Middle vs large sized | 15:105200001-105300001 | 0.237 | 250 | TMEM237;C2CD6;MPP4 | C2CD6;TRAK2;ALS2;MPP4;TMEM237;CDK15;STRADB |
| Middle vs large sized | 15:105300001-105400001 | 0.189 | 272 | ALS2;MPP4 | TMEM237;CDK15;ALS2;C2CD6;MPP4 |
| Cosmopolitan-derived vs local | 1:159800001-159900001 | 0.267 | 901 | CDH20 | RNF152;CDH20 |
| Cosmopolitan-derived vs local | 1:161400001-161500001 | 0.213 | 845 | CCBE1 | LMAN1;RAX;CPLX4;CCBE1 |
| Cosmopolitan-derived vs local | 5:29300001-29400001 | 0.220 | 863 | LOC106510322 | GNS;RASSF3;TBC1D30;WIF1;LOC106510322;LEMD3 |
| Cosmopolitan-derived vs local | 9:85100001-85200001 | 0.213 | 689 | - | AGMO;MEOX2 |
| Cosmopolitan-derived vs local | 9:87300001-87400001 | 0.231 | 586 | HDAC9 | PRPS1L1;HDAC9 |
| Cosmopolitan-derived vs local | 10:39900001-40000001 | 0.214 | 1074 | WAC;LOC100621751 | WAC;BAMBI;LOC102161970;MPP7;LOC100621751 |
| Cosmopolitan-derived vs local | 11:39700001-39800001 | 0.222 | 1141 | - | - |
| Cosmopolitan-derived vs local | 11:39900001-40000001 | 0.212 | 940 | - | - |
| Cosmopolitan-derived vs local | 13:167400001-167500001 | 0.223 | 1011 | LOC100738961 | LOC100738961 |
| Cosmopolitan-derived vs local | 15:25500001-25600001 | 0.212 | 1427 | GYPC | TEX51;GYPC;BIN1 |
| Cosmopolitan-derived vs local | 15:25600001-25700001 | 0.226 | 1500 | GYPC | TEX51;GYPC |
| Cosmopolitan-derived vs local | 15:105200001-105300001 | 0.220 | 250 | TMEM237;C2CD6;MPP4 | C2CD6;TRAK2;ALS2;MPP4;TMEM237;CDK15;STRADB |
| Wild Boar vs local | 1:116300001-116400001 | 0.402 | 534 | DNAAF4;PYGO1 | RAB27A;PIGB;PYGO1;CCPG1;PRTG;C1H15orf65;PIGBOS1;DNAAF4 |
| Wild Boar vs local | 1:160900001-161000001 | 0.430 | 768 | - | PMAIP1;LOC110261667;MC4R |
| Wild Boar vs local | 1:265400001-265500001 | 0.413 | 167 | NR6A1 | OLFML2A;ARPC5L;NR6A1;RPL35;ADGRD2;GOLGA1;NR5A1;PSMB7;WDR38 |
| Wild Boar vs local | 1:265500001-265600001 | 0.507 | 579 | OLFML2A;NR6A1 | SCAI;OLFML2A;ARPC5L;NR6A1;RPL35;GOLGA1;NR5A1;WDR38 |
| Wild Boar vs local | 7:62000001-62100001 | 0.545 | 811 | - | SSTR1;TTC6;CLEC14A |
| Wild Boar vs local | 7:62100001-62200001 | 0.534 | 766 | - | SSTR1;TTC6 |
| Wild Boar vs local | 7:77800001-77900001 | 0.413 | 597 | SUPT16H;RPGRIP1 | ZNF219;LOC100626020;RNASE13;SUPT16H;CHD8;METTL3;TMEM253;LOC110261607;RAB2B;TOX4;SALL2;RPGRIP1;LOC106504487;HNRNPC;ARHGEF40 |
| Wild Boar vs local | 8:48800001-48900001 | 0.409 | 1077 | - | - |
| Wild Boar vs local | 13:75600001-75700001 | 0.436 | 565 | CEP63;ANAPC13 | CEP63;KY;AMOTL2;ANAPC13 |
| Wild Boar vs local | 15:89000001-89100001 | 0.472 | 810 | - | - |
| Wild Boar vs local | 15:97300001-97400001 | 0.406 | 1228 | - | - |

**Table S15.** Putative deleterious variants that showed a marked allele frequency difference between pig breeds and wild boars (>80% in one group, <20% in the other, and vice versa).

| **Chr1** | **POSg2** | **Ref3** | **Alt4** | **ENSEMBL Gene ID** | **Gene name** | **POSp5** | **SAP6** | **UniProt ACC** | **SIFT*7** | **FWB8** | **FPIG9** | **A/R10** | **Class** |
| --- | --- | --- | --- | --- | --- | --- | --- | --- | --- | --- | --- | --- | --- |
| 1 | 52986683 | G | A | ENSSSCG00000004283 | DPPA5 | 101 | R/C | F1RTS2 | 0,04 | 0.889 | 0.182 | ALT | nsNSP |
| 1 | 64324059 | C | A | ENSSSCG00000004344 | MMS22L | 1139 | D/Y | F1RXZ9 | 0,01 | 1.000 | 0.083 | ALT | nsNSP |
| 1 | 72748730 | C | T | ENSSSCG00000004371 | CRYBG1 | 1805 | H/Y | K7GPE1 | 0,03 | 1.000 | 0.121 | ALT | nsNSP |
| 1 | 107121769 | C | A | ENSSSCG00000004545 | ANKDD1A | 179 | G/V | A0A287AX50 | 0,02 | 0.833 | 0.119 | ALT | nsNSP |
| 1 | 114533544 | C | T | ENSSSCG00000035038 | CGNL1 | 388 | V/M | A0A286ZRZ8 | 0,00 | 0.857 | 0.108 | ALT | nsNSP |
| 1 | 137008425 | G | A | ENSSSCG00000040466 | FMN1 | 37 | S/F | A0A287B9C3 | 0,00 | 0.857 | 0.049 | ALT | nsNSP |
| 1 | 158643871 | G | A | ENSSSCG00000004896 | PHLPP1 | 1673 | R/W | F1SMW1 | 0,00 | 0.875 | 0.066 | ALT | nsNSP |
| 1 | 188597983 | A | T | ENSSSCG00000037854 | L3HYPDH^ | 120 | L/H | A0A286ZTS1 | 0,00 | 1.000 | 0.115 | ALT | nsNSP |
| 1 | 249940748 | A | G | ENSSSCG00000039808 | - | 30 | V/A | A0A286ZVA7 | 0,00 | 1.000 | 0.195 | ALT | nsNSP |
| 1 | 265577781 | C | T | ENSSSCG00000005593 | OLFML2A* | 72 | R/C | A0A2C9F384 | 0,00 | 1.000 | 0.073 | ALT | nsNSP |
| 3 | 5396493 | C | T | ENSSSCG00000007599 | LMTK2 | 915 | R/C | A0A287BC41 | 0,03 | 0.833 | 0.167 | ALT | nsNSP |
| 3 | 6509226 | C | G | ENSSSCG00000007617 | ZNF655 | 172 | P/A | A0A287B8H2 | 0,00 | 0.875 | 0.096 | ALT | nsNSP |
| 3 | 28815947 | C | A | ENSSSCG00000040412 | - | 277 | R/L | A0A287AT13 | 0,00 | 1.000 | 0.014 | ALT | nsNSP |
| 3 | 71584432 | A | G | ENSSSCG00000033145 | - | 57 | S/P | A0A287BQ20 | 0,00 | 0.857 | 0.016 | ALT | nsNSP |
| 3 | 110232279 | G | T | ENSSSCG00000008534 | TOGARAM2 | 901 | L/M | I3LH82 | 0,00 | 0.917 | 0.082 | ALT | nsNSP |
| 4 | 89880059 | G | A | ENSSSCG00000006380 | SLAMF1 | 84 | V/M | K7GQV7 | 0,01 | 0.875 | 0.057 | ALT | nsNSP |
| 4 | 124003320 | T | C | ENSSSCG00000006897 | CCDC18 | 157 | K/E | A0A287BIW9 | 0,00 | 0.917 | 0.057 | ALT | nsNSP |
| 4 | 127244817 | A | C | ENSSSCG00000030801 | LOC100523310 | 341 | N/T | A0A286ZJQ6 | 0,03 | 1.000 | 0.157 | ALT | nsNSP |
| 4 | 127339439 | G | A | ENSSSCG00000024973 | GBP1 | 357 | D/N | A0A287A3N2 | 0,02 | 0.818 | 0.042 | ALT | nsNSP |
| 5 | 17573283 | G | A | ENSSSCG00000033235 | LOC100621844 | 412 | R/W | A0A287AQ18 | 0,00 | 1.000 | 0.059 | ALT | nsNSP |
| 5 | 62618603 | C | T | ENSSSCG00000000664 | A2ML1 | 714 | R/Q | F1SLW8 | 0,04 | 0.889 | 0.135 | ALT | nsNSP |
| 5 | 82907889 | T | C | ENSSSCG00000000869 | UTP20 | 164 | T/A | F1SRI4 | 0,00 | 0.900 | 0.120 | ALT | nsNSP |
| 6 | 27615687 | C | T | ENSSSCG00000035810 | - | 13 | R/C | A0A287A5Q3 | 0,05 | 1.000 | 0.192 | ALT | nsNSP |
| 6 | 39373832 | G | C | ENSSSCG00000027085 | LOC100522678 | 14 | S/W | A0A287ASX6 | 0,01 | 0.889 | 0.158 | ALT | nsNSP |
| 6 | 51497943 | G | A | ENSSSCG00000040264 | - | 88 | R/H | A0A286ZYA8 | 0,01 | 0.889 | 0.069 | ALT | nsNSP |
| 6 | 55681068 | C | T | ENSSSCG00000038283 | KLK11 | 179 | G/S | A0A287AZX1 | 0,01 | 0.889 | 0.006 | ALT | nsNSP |
| 6 | 57631484 | G | A | ENSSSCG00000002941 | - | 48 | G/R | F1RKL4 | 0,04 | 0.900 | 0.174 | ALT | nsNSP |
| 6 | 58957497 | T | C | ENSSSCG00000032445 | - | 37 | M/V | A0A287BJA7 | 0,00 | 0.923 | 0.023 | ALT | nsNSP |
| 6 | 58984709 | G | T | ENSSSCG00000037426 | LOC100517285 | 581 | G/W | A0A287APE1 | 0,01 | 0.833 | 0.039 | ALT | nsNSP |
| 6 | 59425540 | C | T | ENSSSCG00000003302 | TMEM86B | 95 | E/K | F1RMN2 | 0,03 | 1.000 | 0.025 | ALT | nsNSP |
| 6 | 59727505 | A | G | ENSSSCG00000024661 | ZNF524 | 273 | R/G | I3L8G6 | 0,02 | 0.875 | 0.004 | ALT | nsNSP |
| 6 | 61287263 | T | C | ENSSSCG00000003326 | PEG3 | 518 | K/R | A0A286ZT70 | 0,02 | 0.909 | 0.144 | ALT | nsNSP |
| 6 | 61988327 | G | A | ENSSSCG00000035387 | ZNF550 | 79 | R/C | A0A287BKE4 | 0,03 | 0.933 | 0.152 | ALT | nsNSP |
| 6 | 64389254 | G | A | ENSSSCG00000029744 | PLCH2 | 1118 | D/N | A0A287ADQ6 | 0,04 | 1.000 | 0.071 | ALT | nsNSP |
| 6 | 111146999 | C | T | ENSSSCG00000003719 | KCTD1 | 306 | G/R | I3LMF8 | 0,02 | 0.905 | 0.117 | ALT | nsNSP |
| 6 | 111147007 | G | A | ENSSSCG00000003719 | KCTD1 | 303 | T/M | I3LMF8 | 0,00 | 0.913 | 0.110 | ALT | nsNSP |
| 6 | 111147556 | G | A | ENSSSCG00000003719 | KCTD1 | 120 | P/L | I3LMF8 | 0,04 | 0.984 | 0.035 | ALT | nsNSP |
| 6 | 164390657 | C | G | ENSSSCG00000031778 | LOC110255328 | 507 | P/A | A0A286ZS15 | 0,00 | 0.875 | 0.123 | ALT | nsNSP |
| 6 | 168668173 | C | T | ENSSSCG00000027374 | C6H1orf50 | 324 | R/Q | I3LLQ7 | 0,00 | 1.000 | 0.160 | ALT | nsNSP |
| 6 | 168670545 | T | C | ENSSSCG00000039676 | - | 31 | F/L | A0A286ZZF9 | 0,02 | 0.857 | 0.199 | ALT | nsNSP |
| 6 | 168670547 | C | A | ENSSSCG00000039676 | - | 31 | F/L | A0A286ZZF9 | 0,02 | 0.875 | 0.195 | ALT | nsNSP |
| 6 | 168670557 | T | C | ENSSSCG00000039676 | - | 35 | W/R | A0A286ZZF9 | 0,00 | 0.857 | 0.191 | ALT | nsNSP |
| 7 | 2439314 | C | A | ENSSSCG00000001000 | ECI2 | 201 | S/I | F1RWZ4 | 0,03 | 1.000 | 0.058 | ALT | nsNSP |
| 7 | 24070080 | T | C | ENSSSCG00000001427 | C4A | 264 | M/T | F1RQW2 | 0,00 | 1.000 | 0.161 | ALT | nsNSP |
| 7 | 54835480 | A | G | ENSSSCG00000001837 | FANCI | 302 | I/V | A0A287AD78 | 0,04 | 0.818 | 0.058 | ALT | nsNSP |
| 7 | 76850671 | C | A | ENSSSCG00000039980 | - | 38 | R/L | A0A287BFA7 | 0,00 | 1.000 | 0.142 | ALT | nsNSP |
| 7 | 77862705 | G | C | ENSSSCG00000002129 | RPGRIP1§ | 834 | A/G | F1S8J2 | 0,04 | 0.000 | 0.939 | REF | nsNSP |
| 7 | 77865573 | T | A | ENSSSCG00000002129 | RPGRIP1§ | 508 | Q/L | F1S8J2 | 0,02 | 0.083 | 0.852 | REF | nsNSP |
| 7 | 80873504 | G | T | ENSSSCG00000033958 | RYR3 | 928 | L/I | A0A287AQ64 | 0,03 | 1.000 | 0.174 | ALT | nsNSP |
| 7 | 115985002 | G | A | ENSSSCG00000002487 | SERPINA3-2 | 95 | T/I | F1SCD1 | 0,05 | 1.000 | 0.183 | ALT | nsNSP |
| 7 | 117465468 | T | A | ENSSSCG00000027030 | BDKRB2 | 62 | M/K | I3LMT9 | 0,00 | 0.833 | 0.139 | ALT | nsNSP |
| 8 | 73410875 | T | A | ENSSSCG00000008990 | MRPL1 | 302 | L/* | A0A287AF82 |  | 0.923 | 0.047 | ALT | SG |
| 9 | 13017984 | G | A | ENSSSCG00000035703 | - | 14 | C/Y | A0A287B052 | 0,05 | 0.867 | 0.148 | ALT | nsNSP |
| 9 | 60835432 | G | A | ENSSSCG00000028123 | VPS26B | 393 | C/Y | A0A286ZMQ9 | 0,00 | 0.818 | 0.159 | ALT | nsNSP |
| 9 | 67589079 | C | A | ENSSSCG00000030821 | FCAMR | 329 | V/L | K7GP71 | 0,00 | 1.000 | 0.190 | ALT | nsNSP |
| 9 | 71100332 | C | G | ENSSSCG00000015308 | - | 221 | G/A | F1S319 | 0,01 | 1.000 | 0.175 | ALT | nsNSP |
| 10 | 12498391 | G | T | ENSSSCG00000034443 | - | 38 | V/F | A0A286ZIQ4 | 0,00 | 1.000 | 0.183 | ALT | nsNSP |
| 10 | 15206200 | G | A | ENSSSCG00000010866 | SCCPDH | 417 | R/C | F1S8P1 | 0,04 | 0.874 | 0.062 | ALT | nsNSP |
| 10 | 15206238 | G | C | ENSSSCG00000010866 | SCCPDH | 404 | A/G | F1S8P1 | 0,00 | 0.892 | 0.061 | ALT | nsNSP |
| 10 | 24930150 | C | T | ENSSSCG00000010929 | CYB5R1 | 121 | V/M | F1S4N2 | 0,01 | 0.916 | 0.035 | ALT | nsNSP |
| 10 | 25822398 | C | T | ENSSSCG00000010936 | - | 89 | W/* | A0A287B9U4 |  | 1.000 | 0.161 | ALT | SG |
| 10 | 64994000 | C | T | ENSSSCG00000022849 | IL2RA | 270 | R/* | I3LG43 |  | 1.000 | 0.064 | ALT | SG |
| 11 | 16488745 | T | C | ENSSSCG00000009385 | SERPINE3 | 269 | D/G | A0A286ZJ01 | 0,02 | 0.846 | 0.097 | ALT | nsNSP |
| 11 | 20664476 | A | G | ENSSSCG00000009407 | ESD | 196 | E/G | Q9GJT2 | 0,02 | 0.818 | 0.098 | ALT | nsNSP |
| 12 | 3737808 | G | A | ENSSSCG00000035360 | TMEM235 | 144 | S/L | A0A286ZPI6 | 0,00 | 0.167 | 0.879 | REF | nsNSP |
| 12 | 6582094 | A | T | ENSSSCG00000035960 | - | 191 | */L | A0A287B5J1 |  | 0.833 | 0.146 | ALT | SL |
| 12 | 11203432 | A | G | ENSSSCG00000032844 | LOC100626206 | 765 | N/S | A0A287BEI0 | 0,00 | 0.833 | 0.027 | ALT | nsNSP |
| 12 | 21235654 | C | G | ENSSSCG00000021755 | LOC100516036 | 118 | P/A | A0A287ART4 | 0,02 | 1.000 | 0.166 | ALT | nsNSP |
| 12 | 21465777 | G | A | ENSSSCG00000036323 | - | 57 | V/M | A0A286ZVK2 | 0,03 | 0.833 | 0.106 | ALT | nsNSP |
| 13 | 51813079 | C | T | ENSSSCG00000036126 | - | 120 | C/Y | A0A287BML2 | 0,03 | 0.125 | 0.833 | REF | nsNSP |
| 13 | 61495416 | C | T | ENSSSCG00000034482 | - | 2 | S/L | A0A287A5F6 | 0,05 | 0.818 | 0.178 | ALT | nsNSP |
| 13 | 89095172 | A | C | ENSSSCG00000035354 | - | 35 | K/T | A0A287APP2 | 0,02 | 1.000 | 0.171 | ALT | nsNSP |
| 13 | 90814192 | G | A | ENSSSCG00000031886 | - | 87 | R/* | A0A287ACF4 |  | 0.846 | 0.014 | ALT | SG |
| 13 | 124971001 | T | C | ENSSSCG00000011806 | MASP1 | 500 | D/G | D5L7X3 | 0,02 | 1.000 | 0.133 | ALT | nsNSP |
| 14 | 12961329 | C | T | ENSSSCG00000038395 | - | 6 | H/Y | A0A287ALL8 | 0,00 | 0.000 | 0.827 | REF | nsNSP |
| 14 | 92113706 | A | G | ENSSSCG00000039475 | - | 447 | L/S | A0A287BLE4 | 0,00 | 0.857 | 0.120 | ALT | nsNSP |
| 15 | 131199995 | T | G | ENSSSCG00000035356 | - | 137 | L/R | A0A286ZYA5 | 0,00 | 0.818 | 0.134 | ALT | nsNSP |
| 15 | 133035808 | C | T | ENSSSCG00000016286 | PRSS56 | 104 | R/W | F1SMT5 | 0,03 | 0.857 | 0.173 | ALT | nsNSP |
| 16 | 25810612 | G | A | ENSSSCG00000029367 | CARD6 | 28 | G/R | I3LI18 | 0,00 | 0.050 | 0.900 | REF | nsNSP |
| 17 | 31861518 | G | T | ENSSSCG00000007143 | MAVS | 152 | A/D | F1S8C6 | 0,02 | 1.000 | 0.008 | ALT | nsNSP |
| 17 | 31936717 | G | A | ENSSSCG00000007149 | SPEF1 | 90 | R/H | D3K5J5 | 0,02 | 1.000 | 0.165 | ALT | nsNSP |
| 17 | 47523550 | T | G | ENSSSCG00000035053 | PI3 | 84 | V/G | A0A287BEB9 | 0,03 | 0.091 | 0.858 | REF | nsNSP |
| 18 | 7336944 | T | G | ENSSSCG00000016469 | TRPV5 | 29 | S/A | A0A287BLI8 | 0,03 | 0.875 | 0.150 | ALT | nsNSP |
| 18 | 39598668 | C | A | ENSSSCG00000016665 | BMPER | 142 | A/S | I3LAF3 | 0,03 | 0.846 | 0.123 | ALT | nsNSP |
| NW_018084979.1 | 2573741 | G | A | ENSSSCG00000035093 | KIF26A | 618 | G/R | A0A287BP20 | 0,00 | 1.000 | 0.034 | ALT | nsNSP |
| NW_018085364.1 | 211285 | C | G | ENSSSCG00000032410 | - | 885 | I/M | A0A286ZNZ5 | 0,01 | 1.000 | 0.069 | ALT | nsNSP |

^1^Chromosome; ^2^Position on the *Sus scrofa* reference genome v.11; ^3^Reference allele; ^4^Alternative allele; ^5^Position on the protein (see. UniProt ACC); ^6^Amino acid substitution; *7*SIFT score of prediction (deleteriousness); ^8^Frequency of the alternative allele in the Wild Boar population; ^9^Average frequency of the alternative allele in the pig breed populations; ^10^ALT indicates that the alternative allele was more frequent (>0.5) in the wild boar population compared with the pig breed populations while REF indicate the opposite. ^gene in the sweep region SSC1:18850000-188600001 identified in the H_P_ analysis of Lithuan indigenus wattle; *gene in the sweep region SSC1:265500001-265600001 near the *NR6A1* one; ^§^genes in the sweep region SSC7:77800001-77900001 identified in the F_ST_ analysis of Wild boar.

**Table S16.** Regions of signatures of selection identified by whole-genome resequencing data produced in this study and SNP chip data produced by Muñoz et al. [30].

| **Pig population** | **Genome Windows (SSC:start-end bp)** | **Annotated genes** | **Annotated genes (±200 Kbp)** |
| --- | --- | --- | --- |
| Alentejana | 1:96500001-96600001 | PIAS2, KATNAL2, LOC110260788 | HDHD2, ST8SIA5, IER3IP1, PIAS2, KATNAL2, LOC110260788, SKOR2 |
| Alentejana | 1:96600001-96700001 | KATNAL2, HDHD2 | HDHD2, IER3IP1, PIAS2, KATNAL2, LOC110260788, SKOR2 |
| Alentejana | 5:29400001-29500001 | WIF1 | TBC1D30, LOC106510322, WIF1, LEMD3, MSRB3 |
| Alentejana | 14:128300001-128400001 | FAM204A | FAM204A, RAB11FIP2 |
| Basque | 4:127900001-128000001 | - | PKN2 |
| Basque | 8:114300001-114400001 | PAPSS1 | PAPSS1, SGMS2 |
| Black Slavonian | 6:1300001-1400001 | - | CA5A, BANP, ZFPM1, LOC110260888, ZNF469, LOC110261212 |
| Black Slavonian | 6:1400001-1500001 | CA5A, BANP | CA5A, BANP, LOC110260888, JPH3, ZNF469, KLHDC4 |
| Casertana | 10:52600001-52700001 | COMMD3 | SPAG6, COMMD3, PIP4K2A, BMI1, DNAJC1 |
| Gascon | 15:18700001-18800001 | NCKAP5 | NCKAP5 |
| Gascon | 15:19600001-19700001 | GPR39 | GPR39, NCKAP5, LYPD1 |
| Krškopolje | 1:74800001-74900001 | - | FOXO3, ARMC2, SESN1 |
| Krškopolje | 8:38700001-38800001 | OCIAD2, OCIAD1, FRYL | OCIAD2, OCIAD1, FRYL, DCUN1D4, CWH43 |
| Lithuanian White Old Type | 13:86200001-86300001 | - | PLOD2 |
| Majorcan Black | 4:29900001-30000001 | ANGPT1 | ANGPT1 |
| Moravka | 1:223900001-224000001 | TRPM3 | KLF9, TRPM3 |
| Sarda | 8:84500001-84600001 | USP38 | USP38, GAB1 |
| Swallow-Bellied Mangalitsa | 2:53300001-53400001 | LOC100515195, LOC100623897 | LOC100623406, LOC100623024, LOC100514828, LOC100622639, LOC110259584, LOC100623897, LOC100623804, LOC100515195, LOC110259736, LOC110259737, LOC100623714, LOC100623613 |
| Swallow-Bellied Mangalitsa | 2:53400001-53500001 | LOC100514828 | LOC100623024, LOC100514828, LOC100622639, LOC100623897, LOC100623804, LOC100515195, LOC100623613, LOC100623714, LOC100622735, LOC106508617 |
