# Supplementary material for: Whole-genome sequencing of European autochthonous and commercial pig breeds allows the detection of signatures of selection for adaptation of genetic resources to different breeding and production systems
Source: Genet Sel Evol. 2020 Jun 26;52:33. doi: 10.1186/s12711-020-00553-7 (PMC7318759; doi:10.1186/s12711-020-00553-7)

**Additional information**

**Whole-genome sequencing of European autochthonous and commercial pig breeds allows the detection of signatures of selection for adaptation of genetic resources to different breeding and production systems**

Samuele Bovo, Anisa Ribani, Maria Muñoz, Estefania Alves, Jose P. Araujo, Riccardo Bozzi, Marjeta Čandek-Potokar, Rui Charneca, Federica Di Palma, Graham Etherington, Ana I. Fernandez, Fabián García, Juan García-Casco, Danijel Karolyi, Maurizio Gallo, Vladimir Margeta, José Manuel Martins, Marie J. Mercat, Giulia Moscatelli, Yolanda Núñez, Raquel Quintanilla, Čedomir Radović, Violeta Razmaite, Juliette Riquet, Radomir Savić, Giuseppina Schiavo, Graziano Usai, Valerio J. Utzeri, Christoph Zimmer, Cristina Ovilo, Luca Fontanesi

**Table of content**

**Additional File 2: Figure S1.** Evaluation of the D-statistics for the Kolmogorov-Smirnov test.

**Additional File 2: Figure S2.** Selection of the window size. (a) The number of windows with less than 10 SNPs over windows of variable size (in the range from 50 to 300-kb) is presented. Red dots represent windows larger than 100 kb, for which the number of windows with less than 10 SNPs started to asymptotically decrease. (b to d) Distribution of the number of SNPs contained in the 50-, 100- and 150-kb windows, respectively.

**Additional File 2: Figure S3.** F_ST_ based Neighbour-Joining tree. Next to the branches, the bootstrap test values expressed as percentage over 10,000 replicates are indicated in red.

**Additional File 2: Figure S4**. Mantel test between F_ST_ distance and the geographical distances (based on longitudinal and latitudinal coordinates) among autochthonous pig populations.

**Additional File 2: Figure S5.** Manhattan plots of the genome-wide H_P_ analyses. Each dot represents a 100-kb genome window.

**Additional File 2: Figure S6.** Manhattan plots of the genome-wide F_ST_ analyses. Each dot represents a 100-kb genome window.

**Additional File 2: Figure S7.** Manhattan plots of the genome-wide FST analysis of breed groups Each dot represents a 100-kb genome window.

**Additional File 2: Figure S8.** Allele frequencies of SNPs in putative regions of signatures of selection detected in the F_ST_ analysis of middle vs large-sized pig breeds. Major signals were detected on: (a) SSC15 that carries the *CASP10* gene, (b) SSC1 that carries the *ARID1B* gene, (c) SSC1 that carries the *MAP3K5* gene (and the nearby *PEX7*) and (d) SSC2 that carries the *PIK3C2A* gene.

**Figure S1.** Evaluation of the D-statistics for the Kolmogorov-Smirnov test.

**
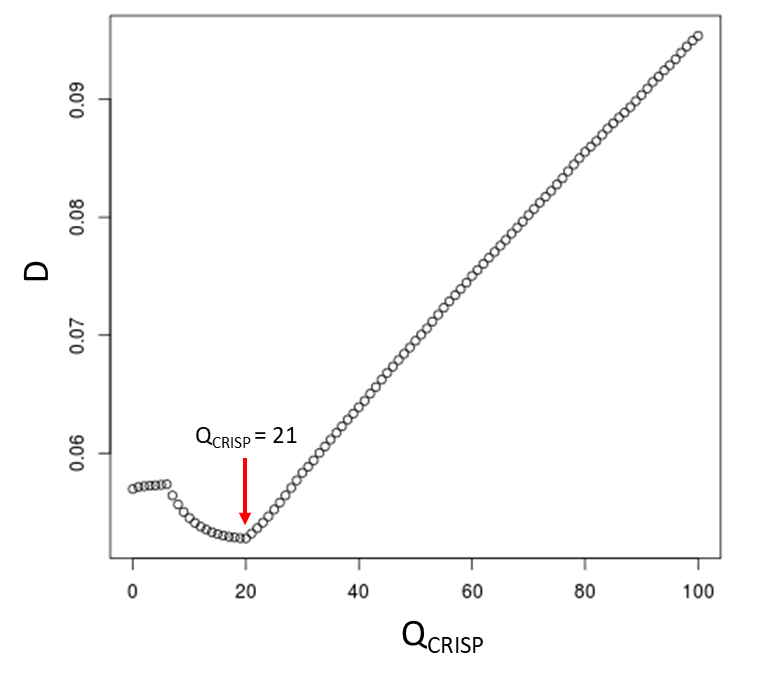
**

**Figure S2.** Selection of the window size. (a) The number of windows with less than 10 SNPs over windows of variable size (in the range from 50 to 300-kb) is presented. Red dots represent windows larger than 100 kb, for which the number of windows with less than 10 SNPs started to asymptotically decrease. (b to d) Distribution of the number of SNPs contained in the 50-, 100- and 150-kb windows, respectively.


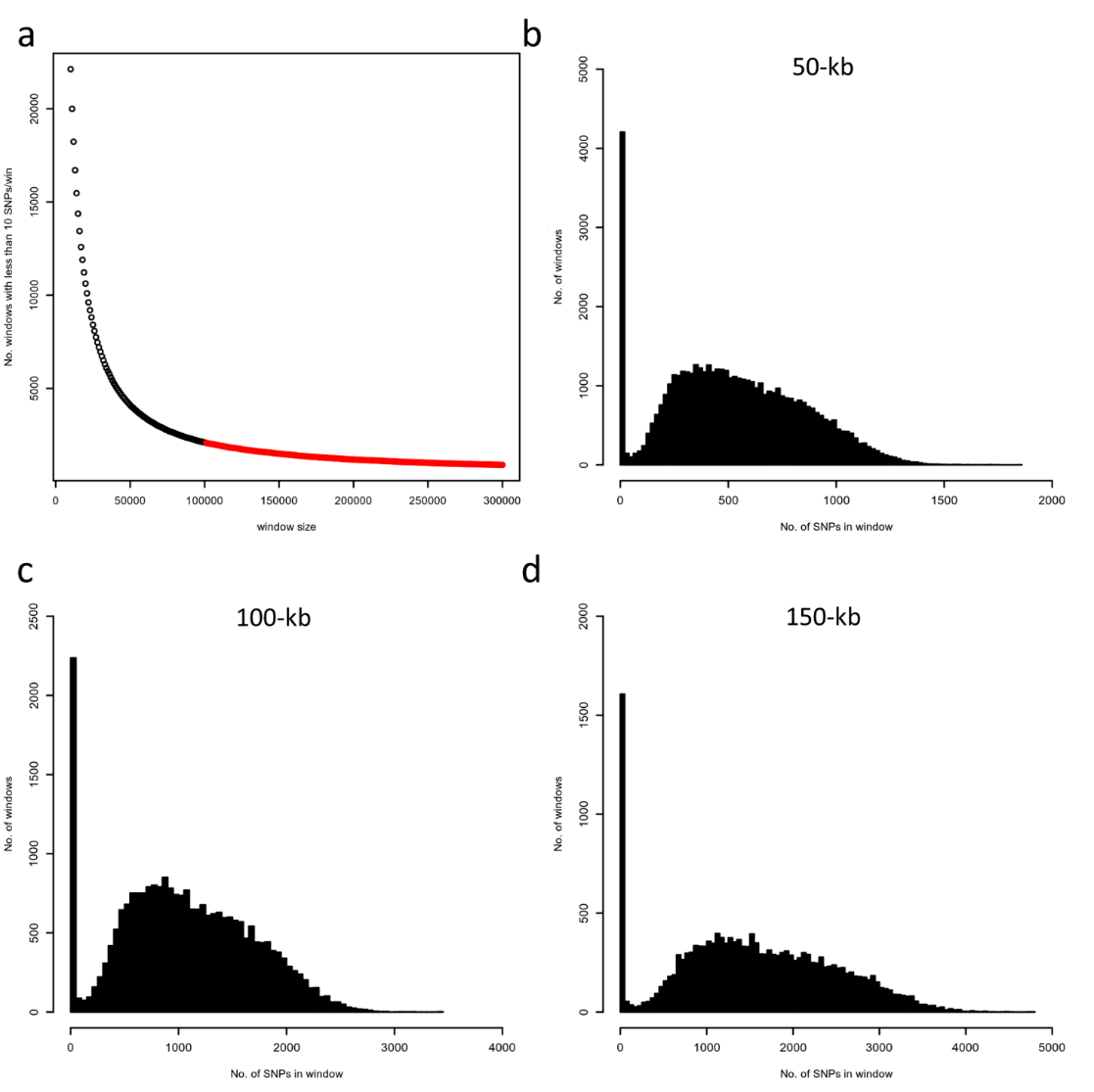


**Figure S3.** F_ST_ based Neighbour-Joining tree. Next to the branches, the bootstrap test values expressed as percentage over 10,000 replicates are indicated in red.


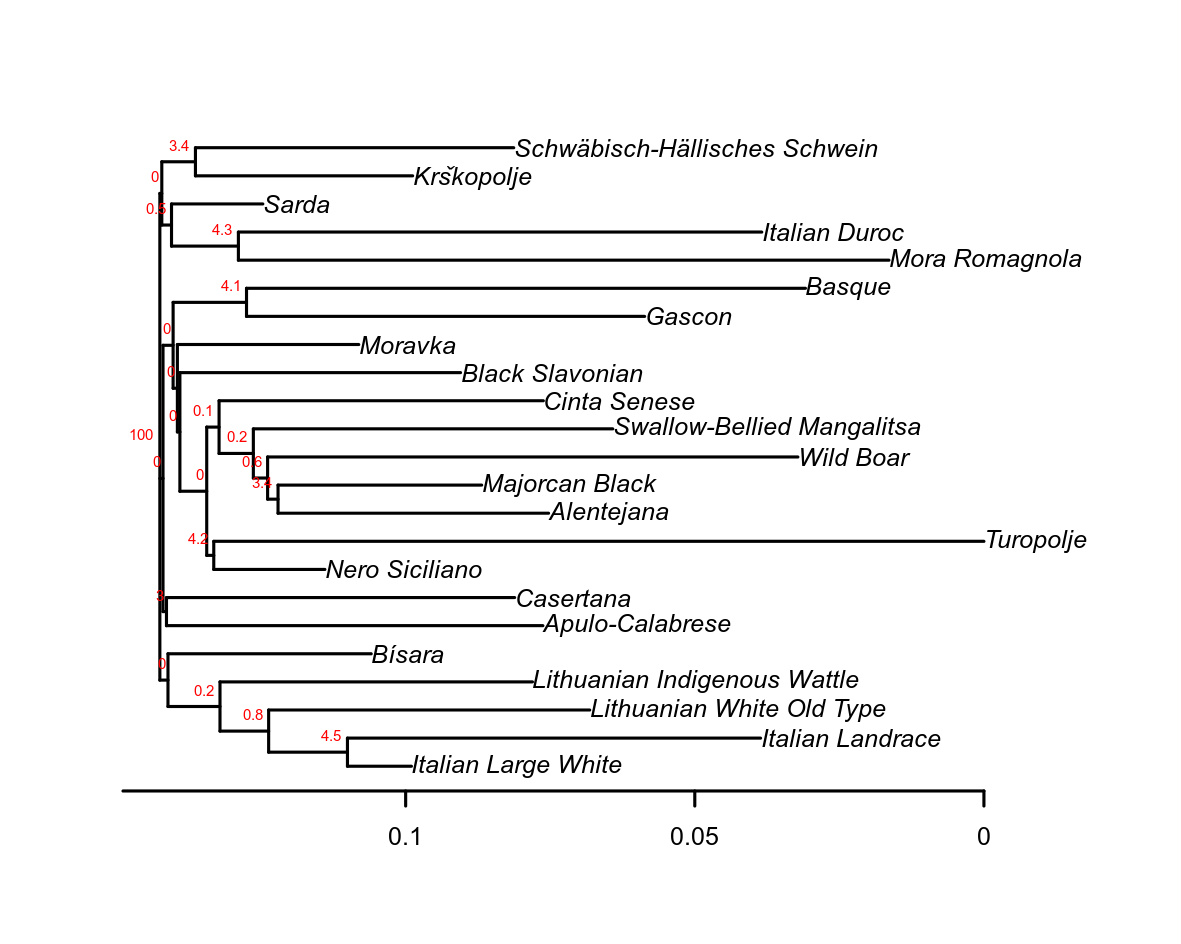


**Figure S4.** Mantel test between F_ST_ distance and the geographical distances (based on longitudinal and latitudinal coordinates) among autochthonous pig populations.

**
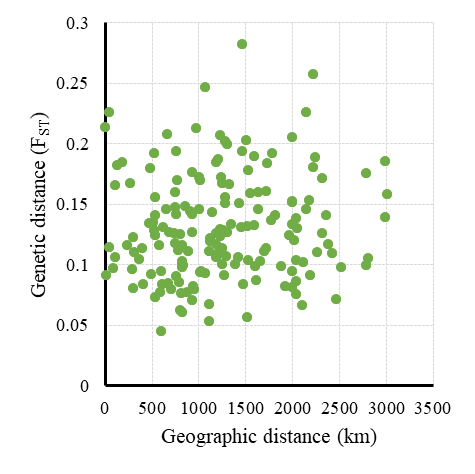
**

**Figure S5.** Manhattan plots of the genome-wide H_P_ analyses. Each dot represents a 100-kb genome window.

**
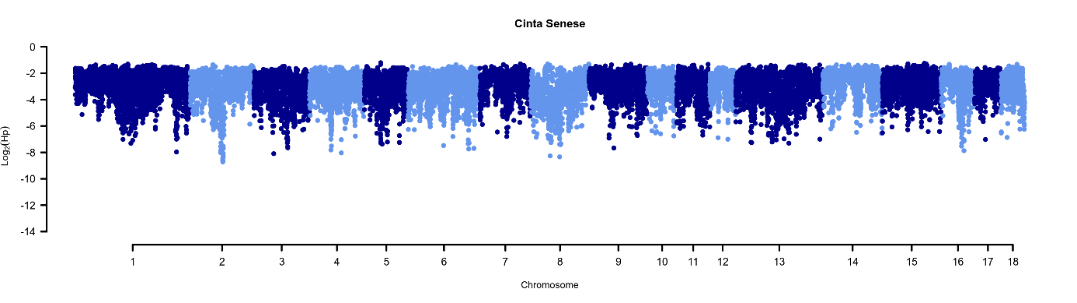

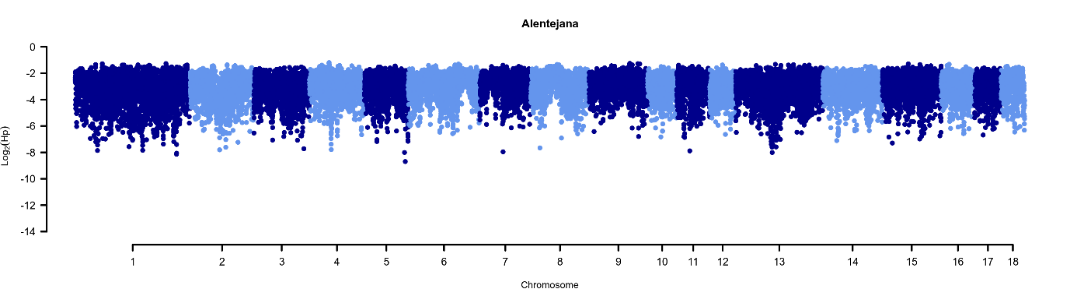

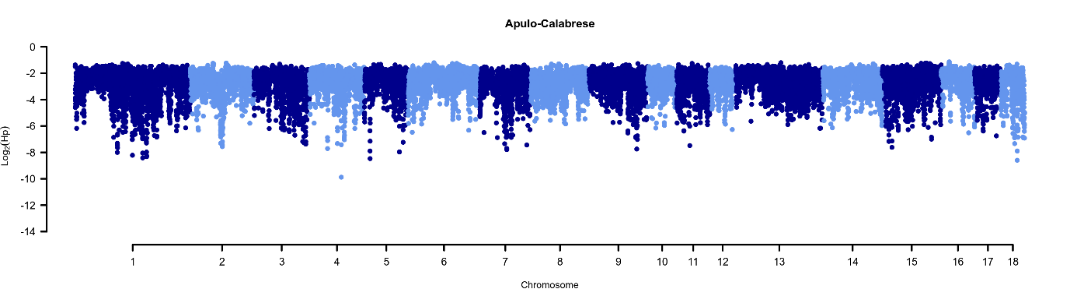

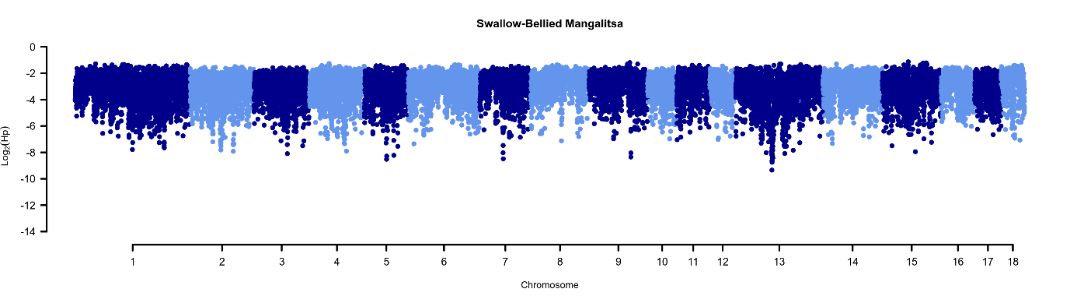

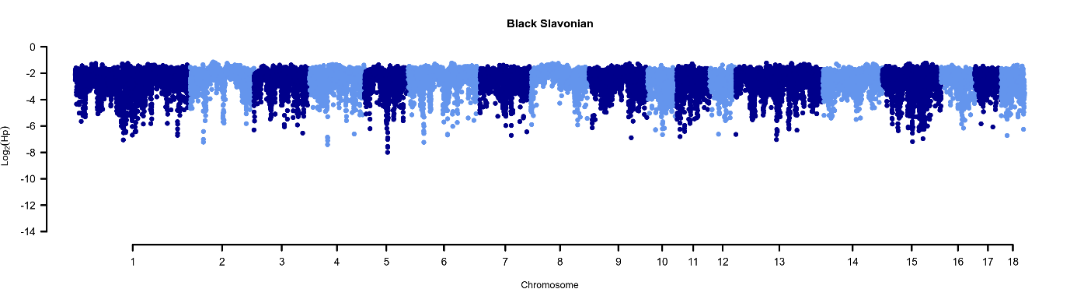

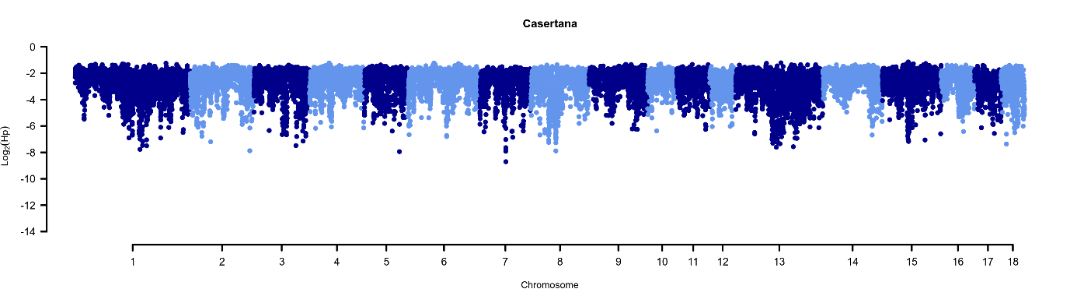

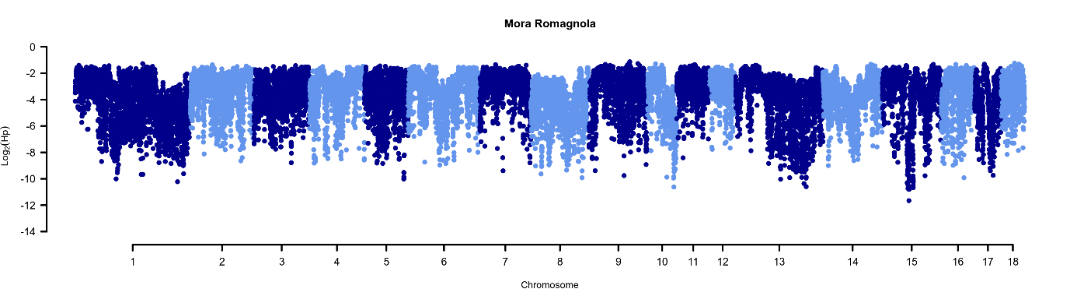

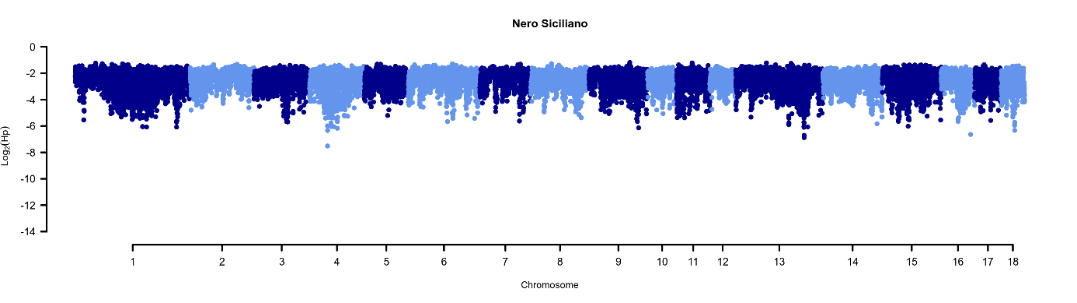

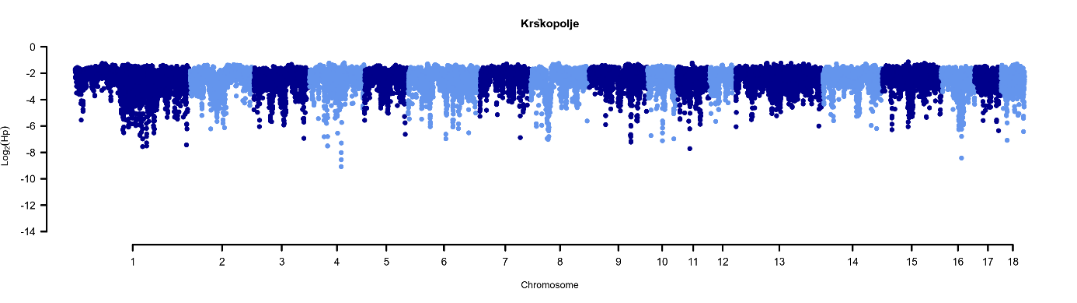

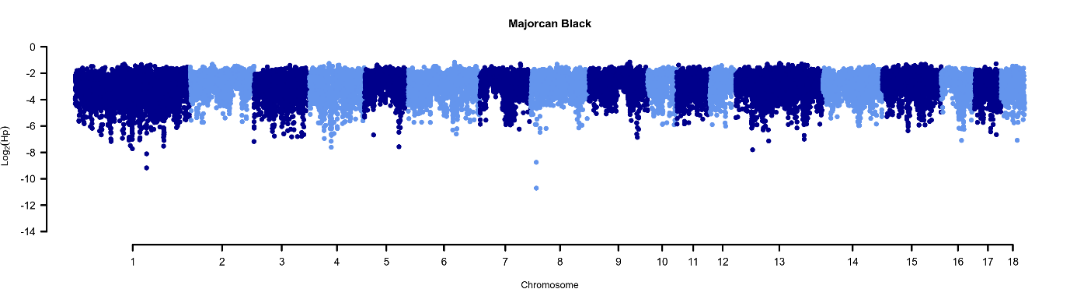

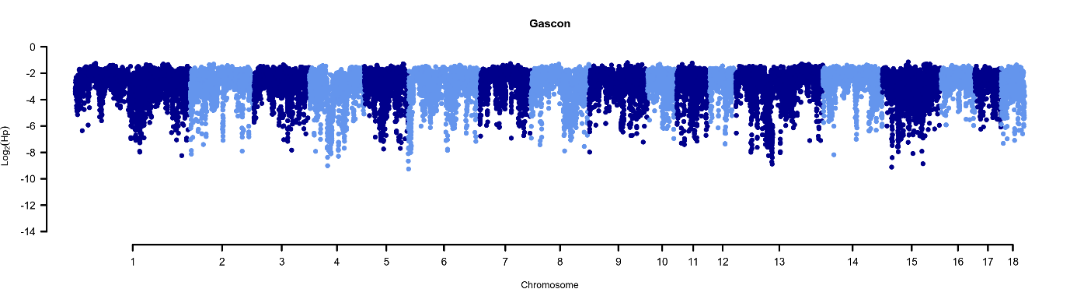

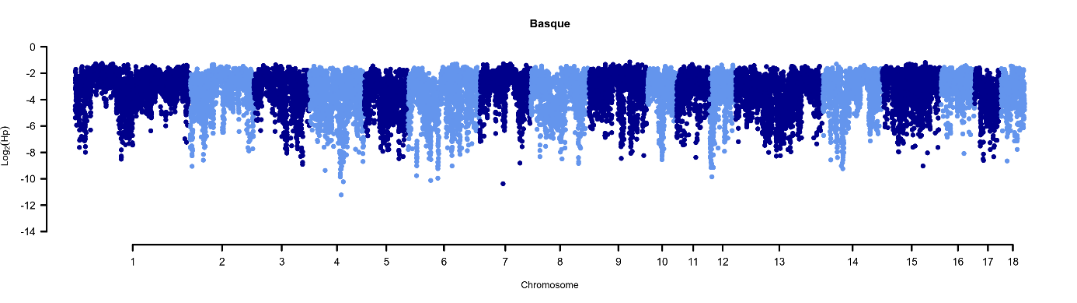

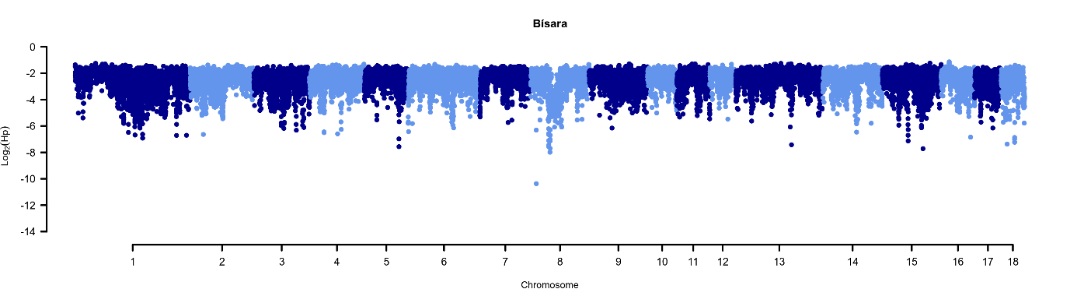

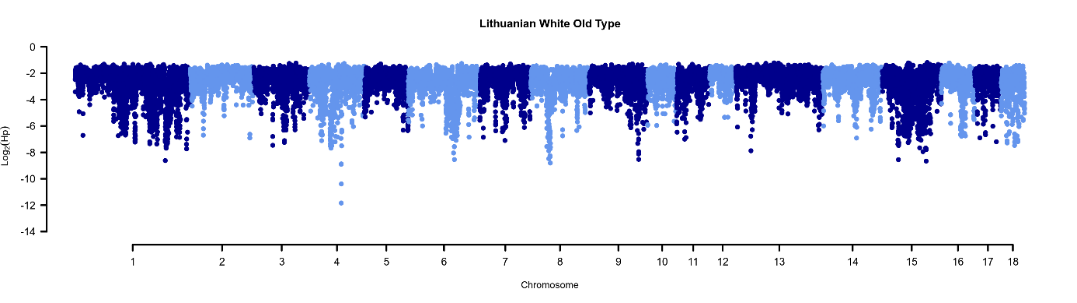

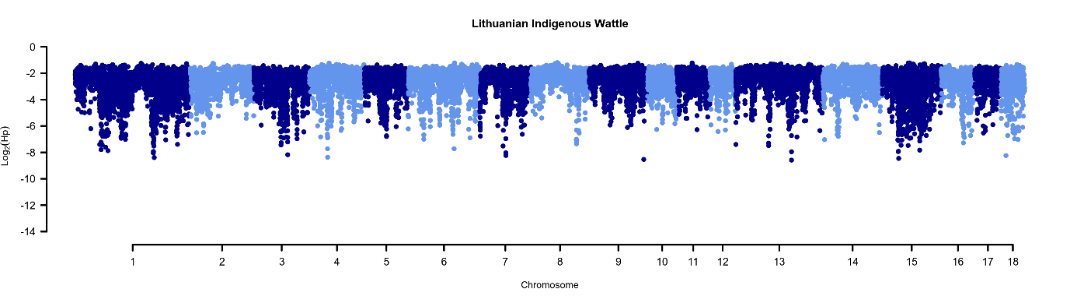

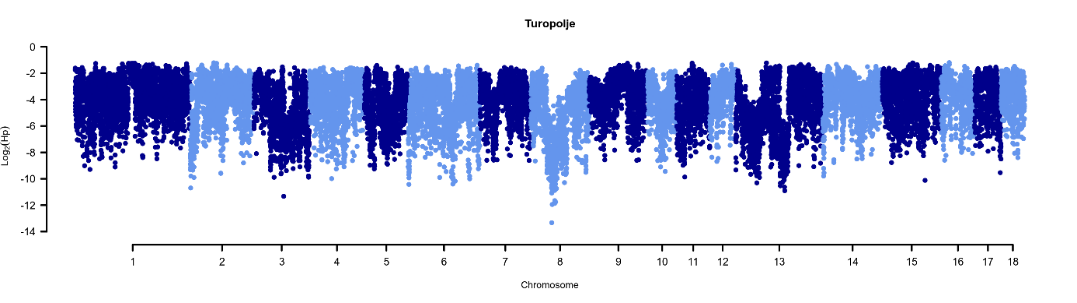

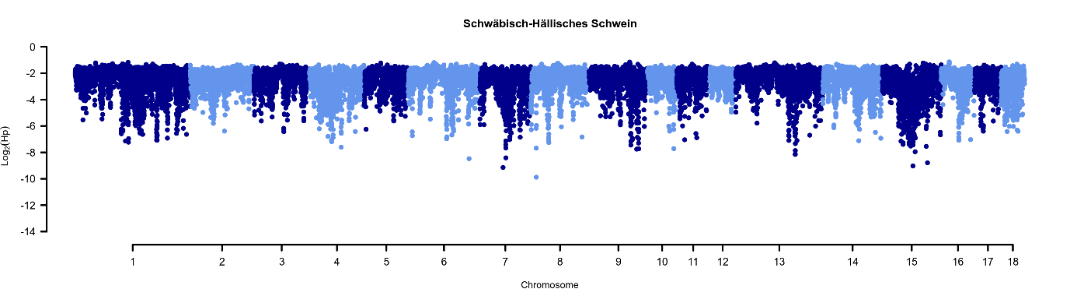

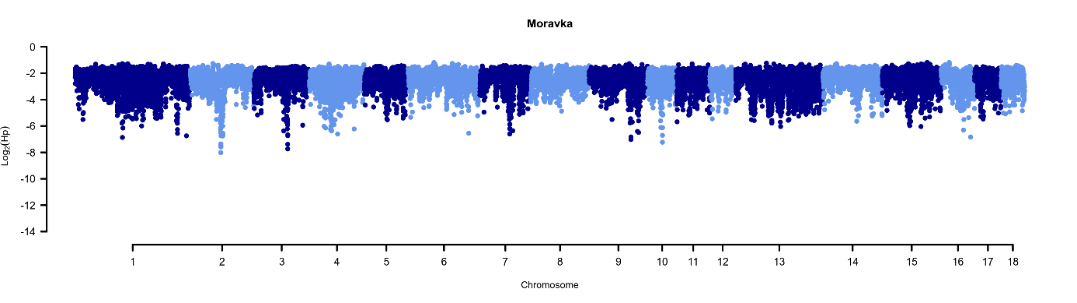

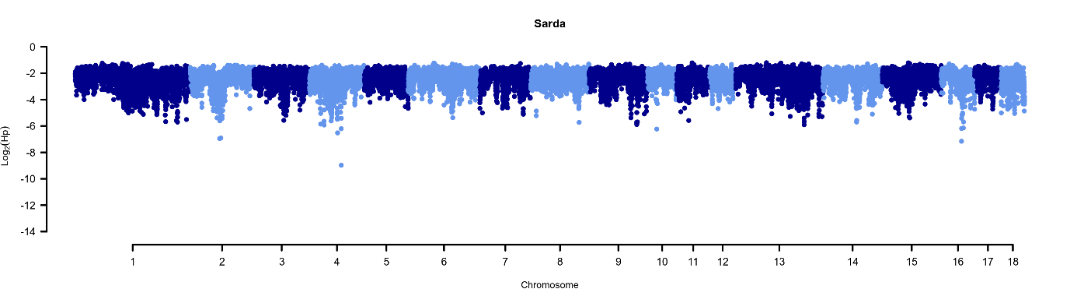

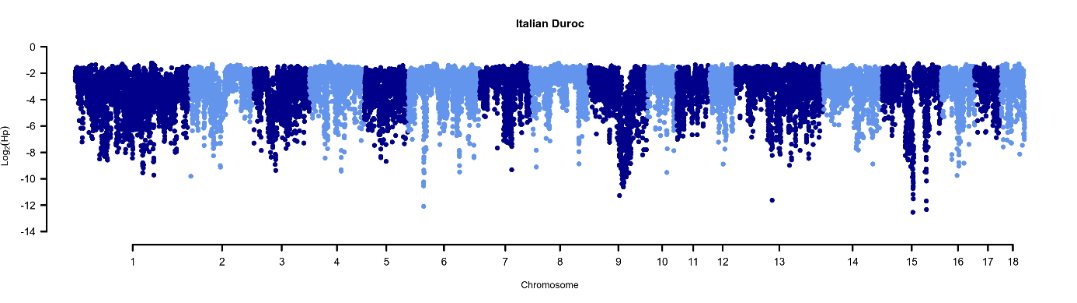

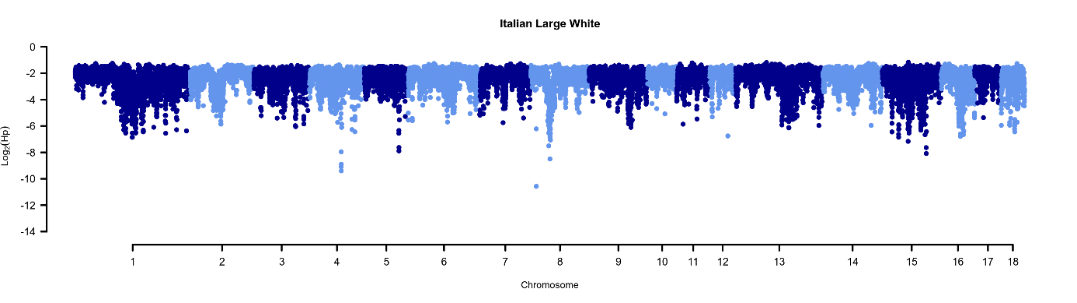

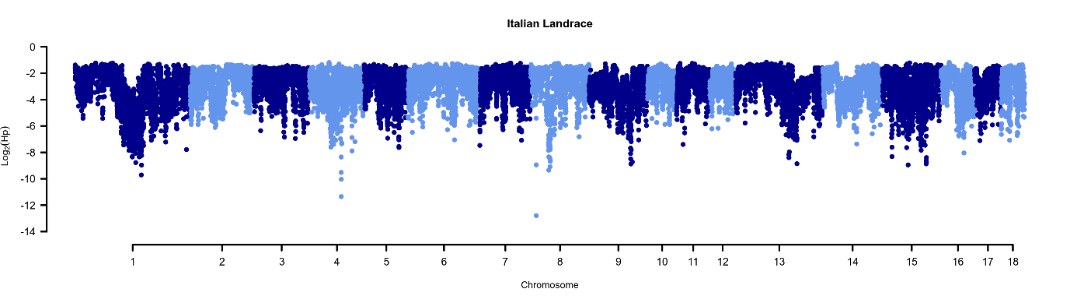

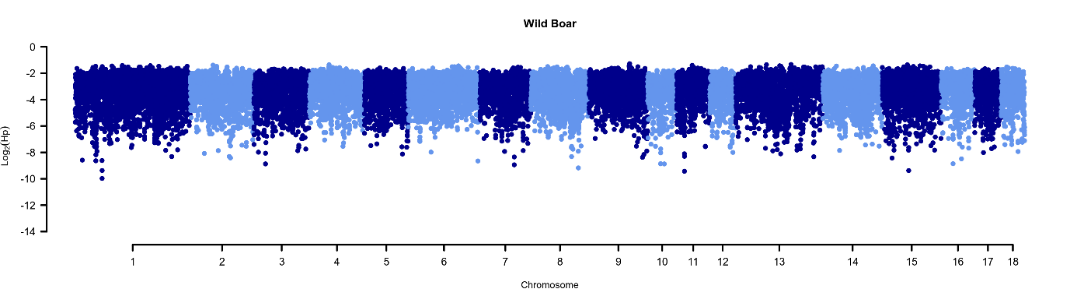
**

**Figure S6.** Manhattan plots of the genome-wide F_ST_ analyses. Each dot represents a 100-kb genome window.

**
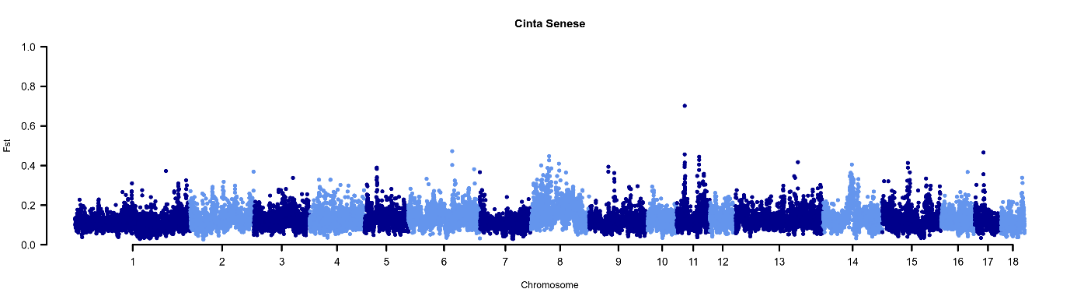

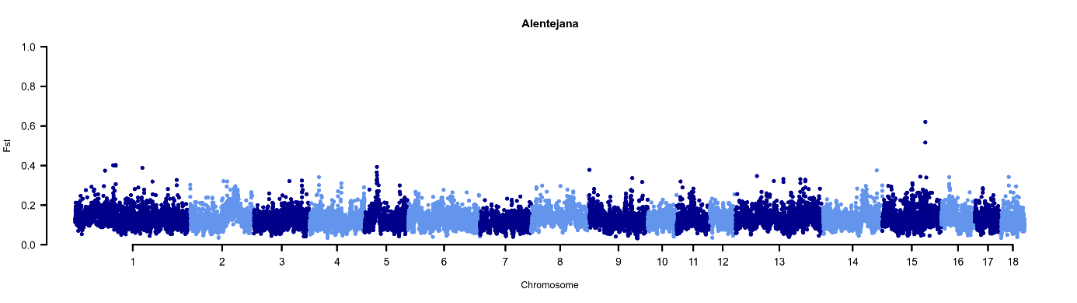

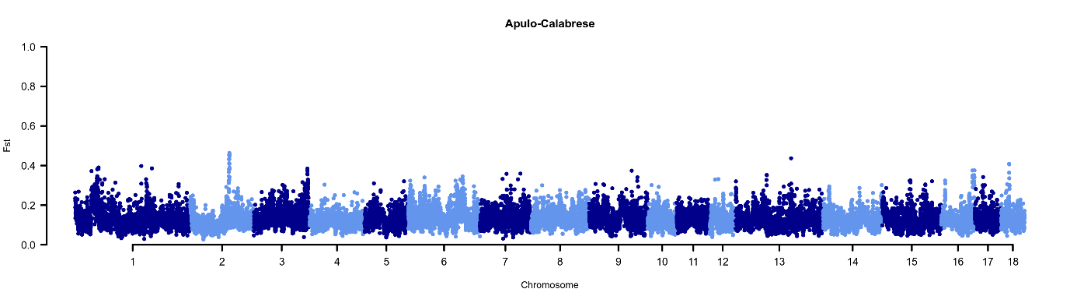

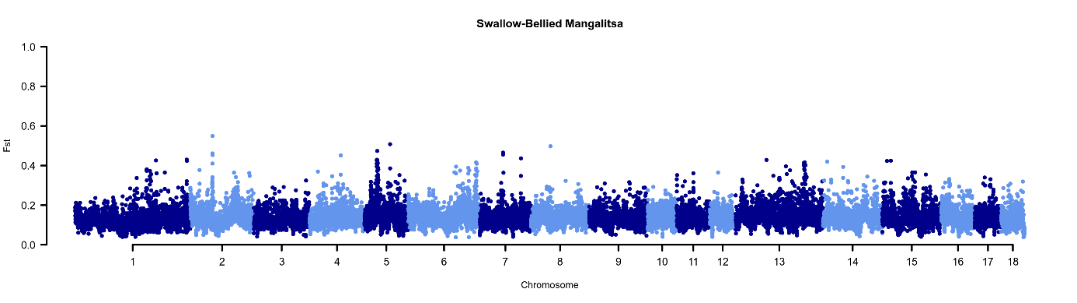

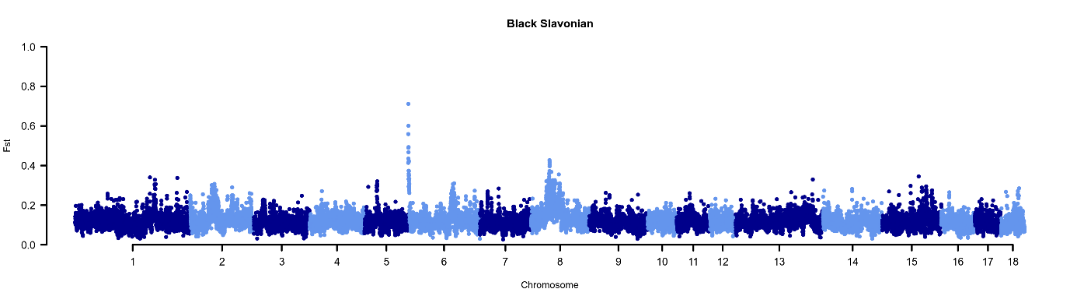

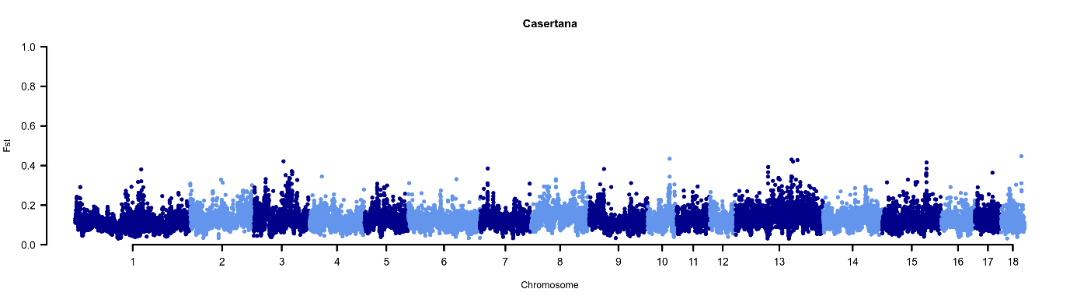

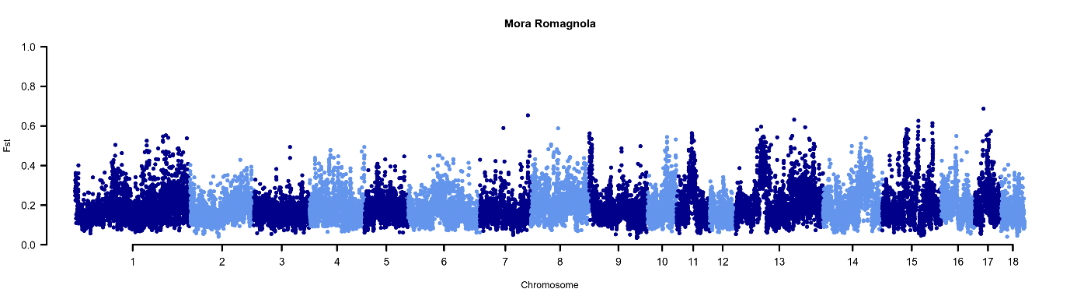

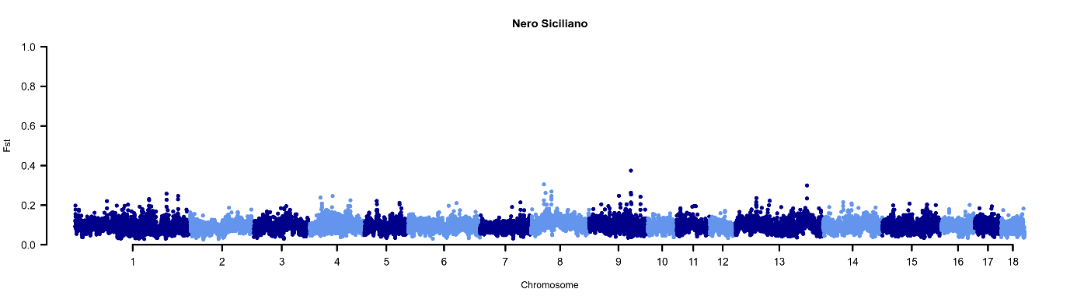

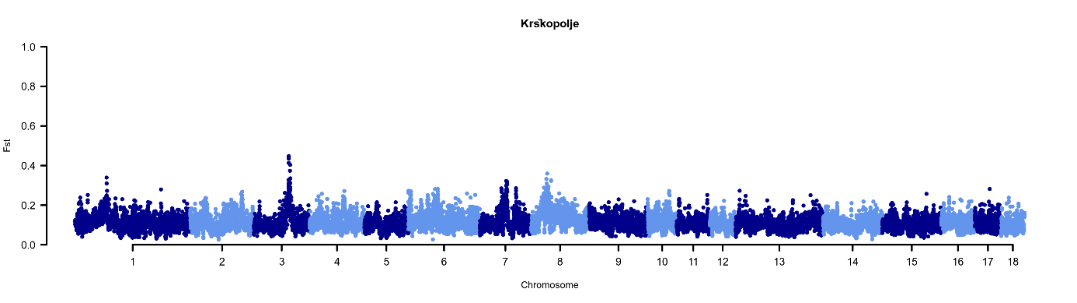

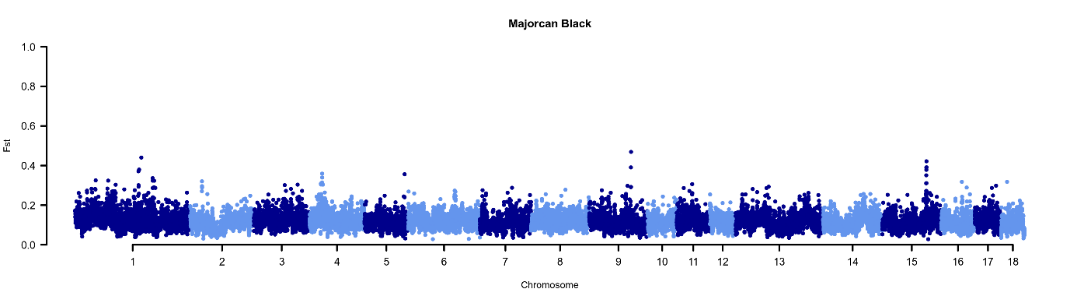

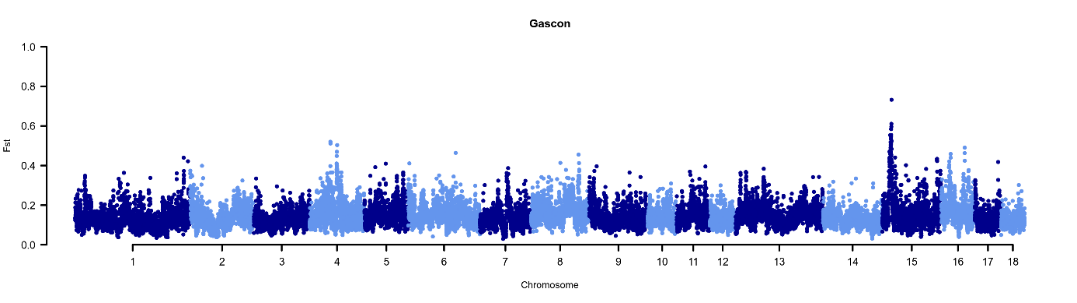

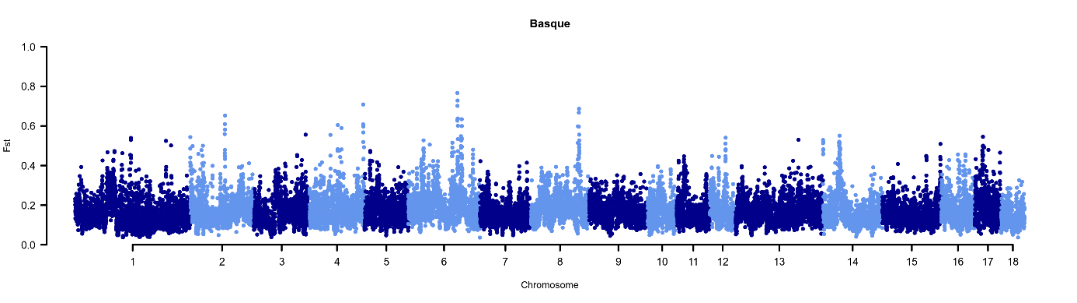

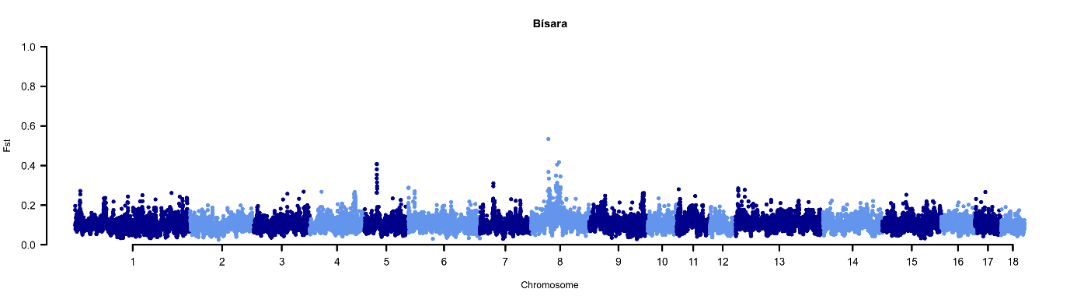

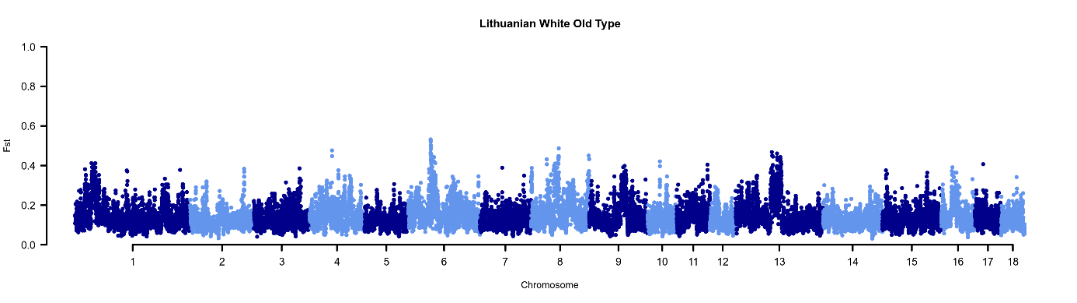

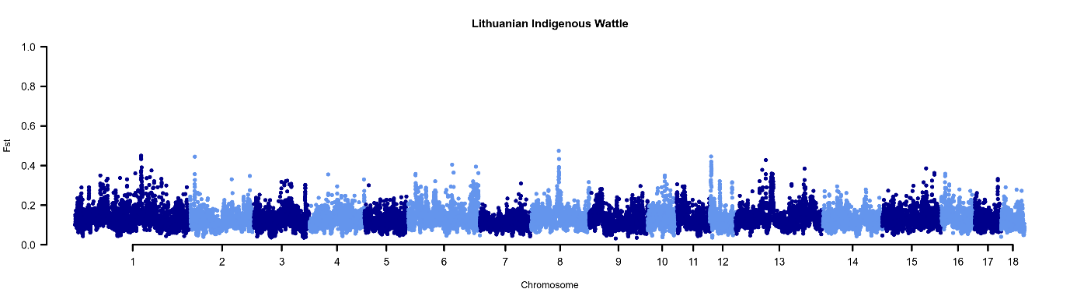

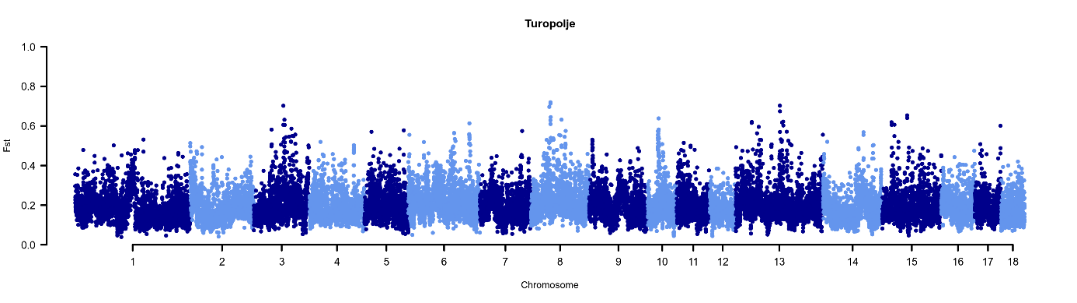

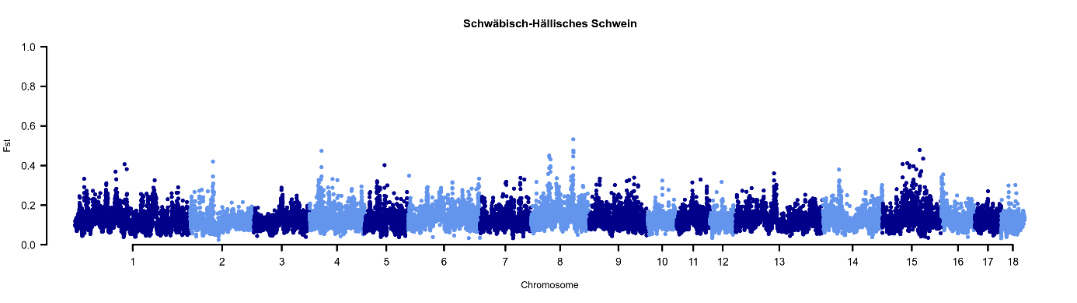

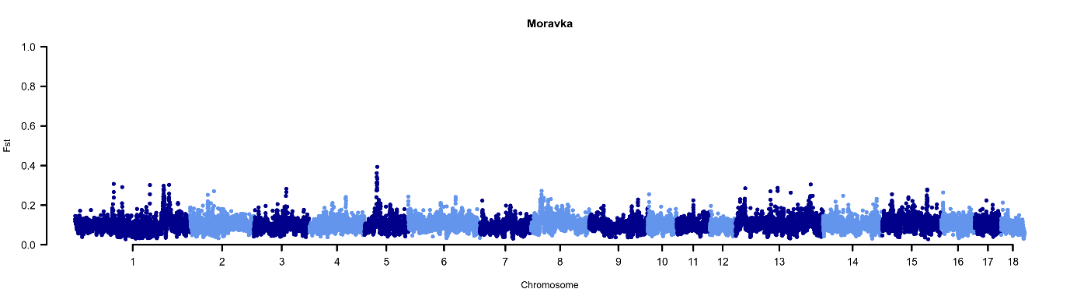

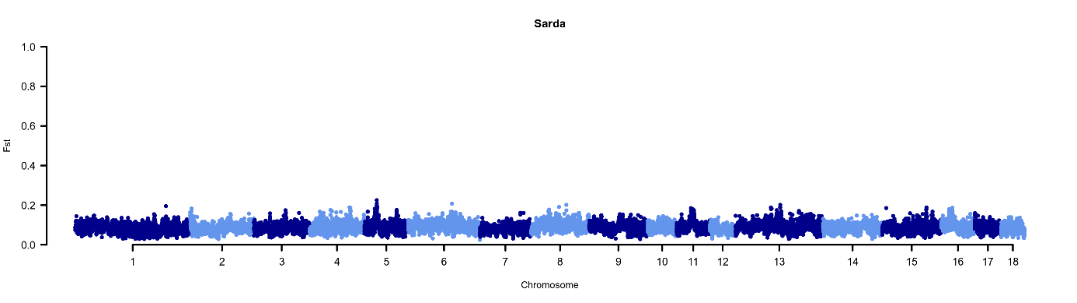

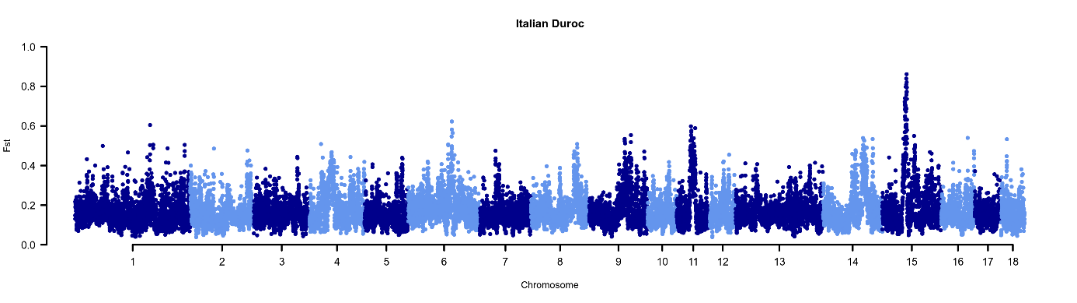

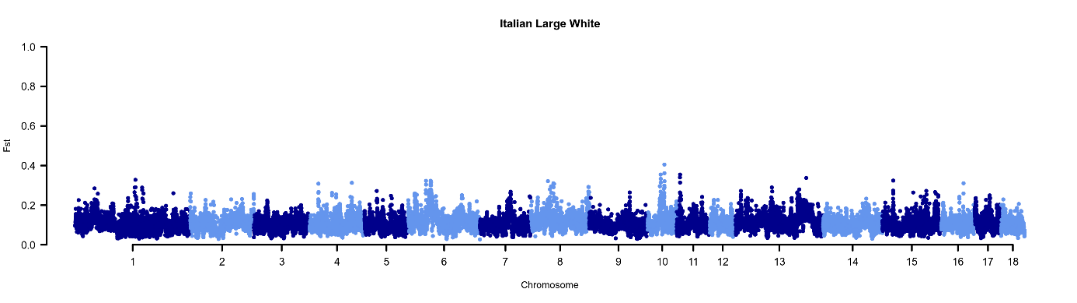

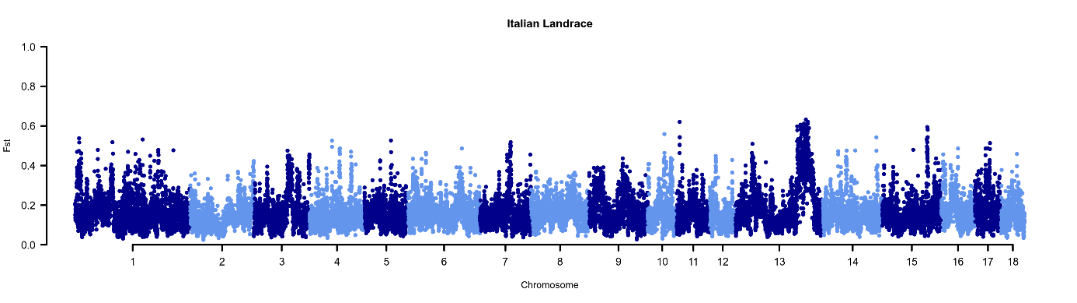

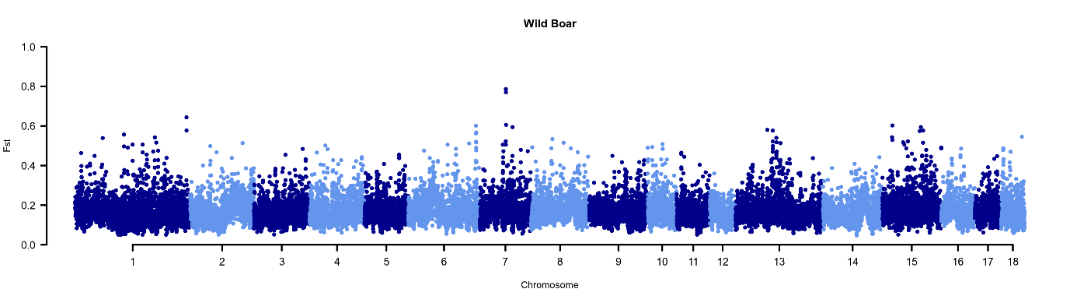
**

**Figure S7.** Manhattan plots of the genome-wide F_ST_ analysis of breed groups Each dot represents a 100-kb genome window.


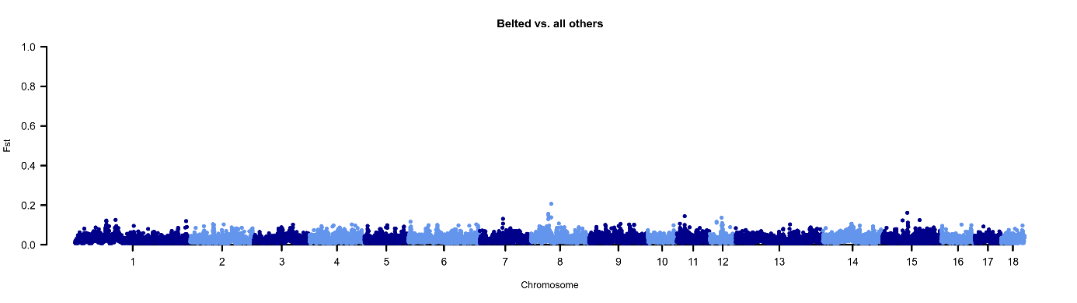

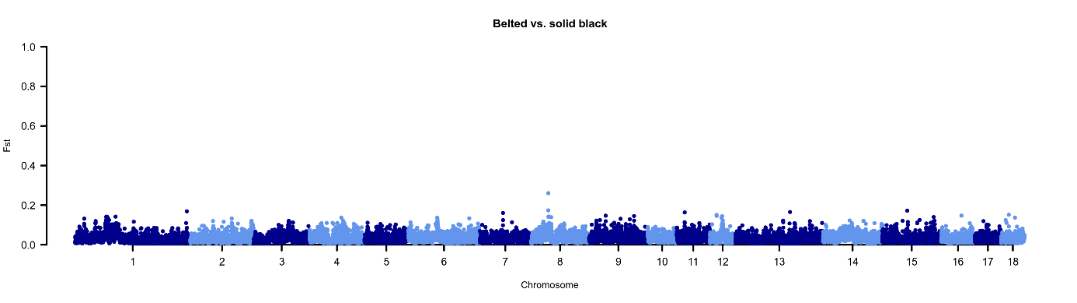

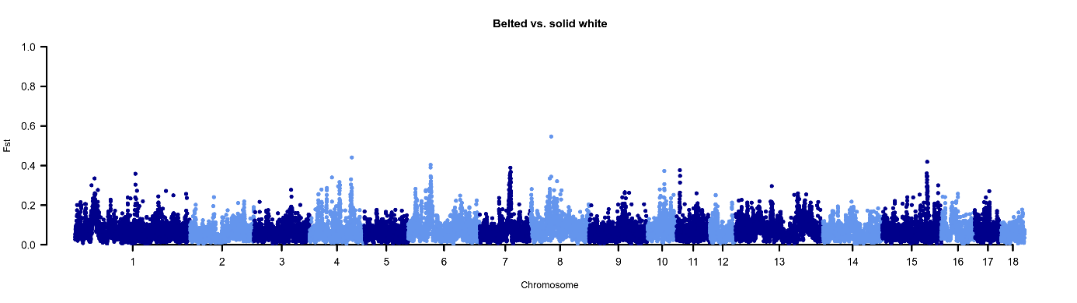

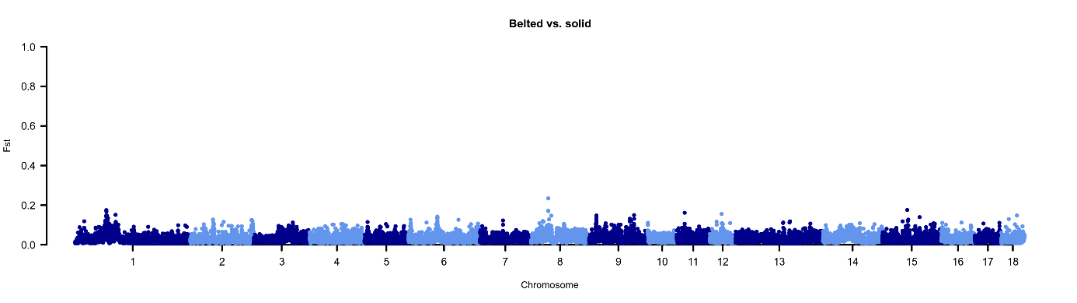

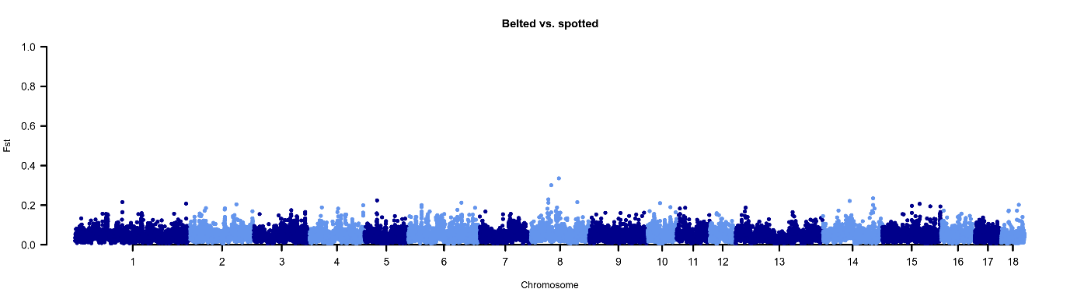

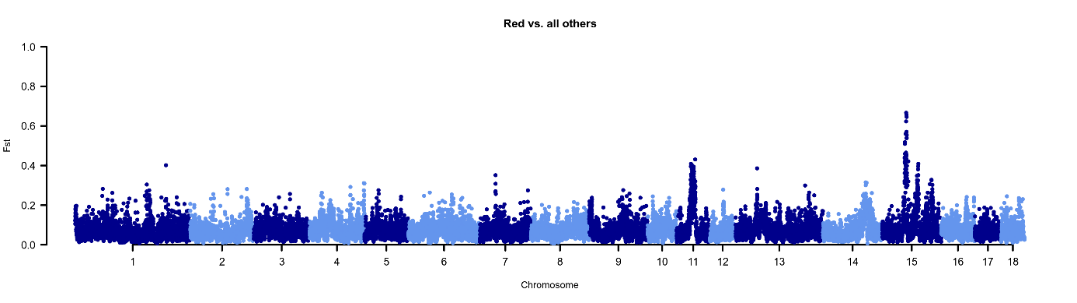

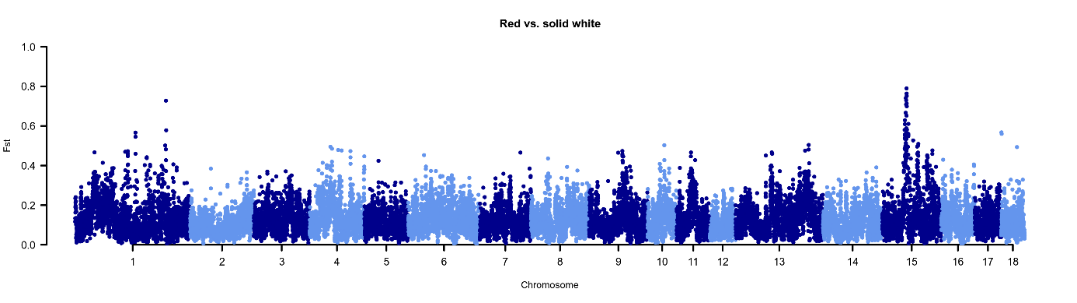


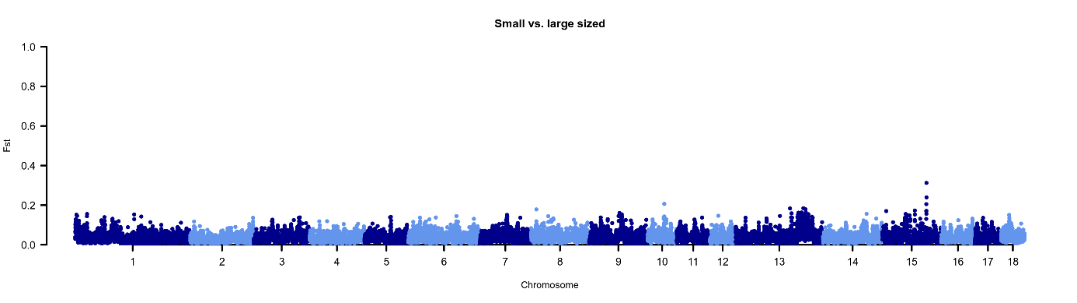


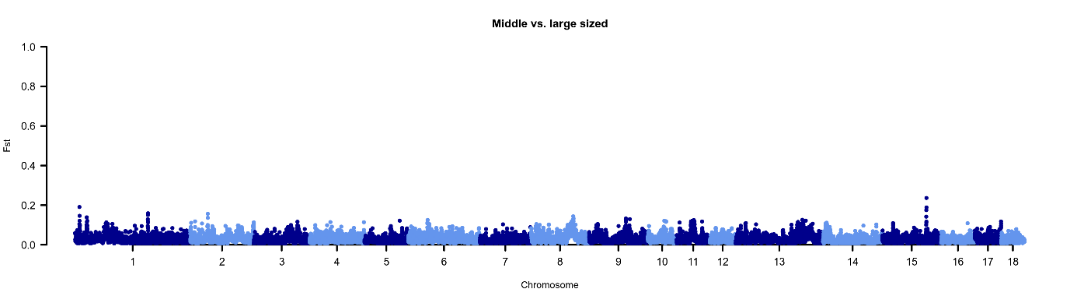


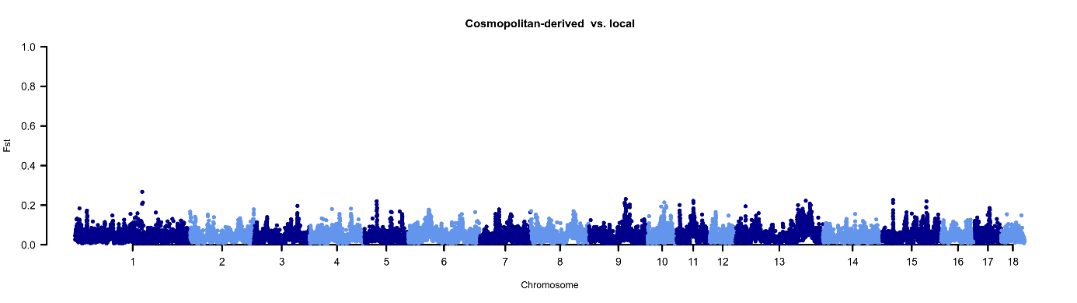


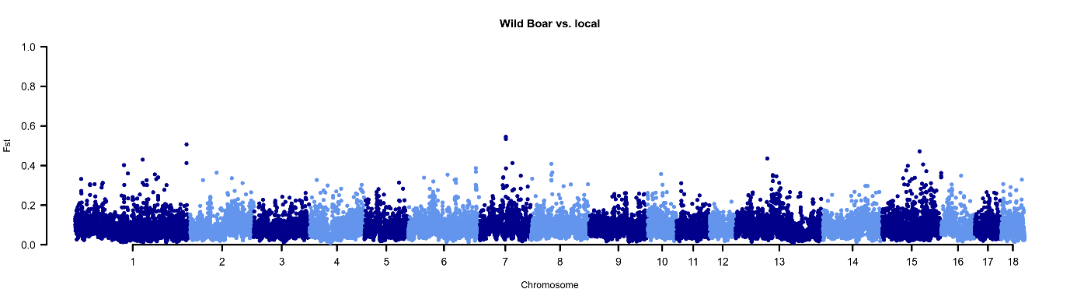


**Figure S8.** Allele frequencies of SNPs in putative regions of signatures of selection detected in the F_ST_ analysis of middle vs large-sized pig breeds. Major signals were detected on: (a) SSC15 that carries the *CASP10* gene, (b) SSC1 that carries the *ARID1B* gene, (c) SSC1 that carries the *MAP3K5* gene (and the nearby *PEX7*) and (d) SSC2 that carries the *PIK3C2A* gene.


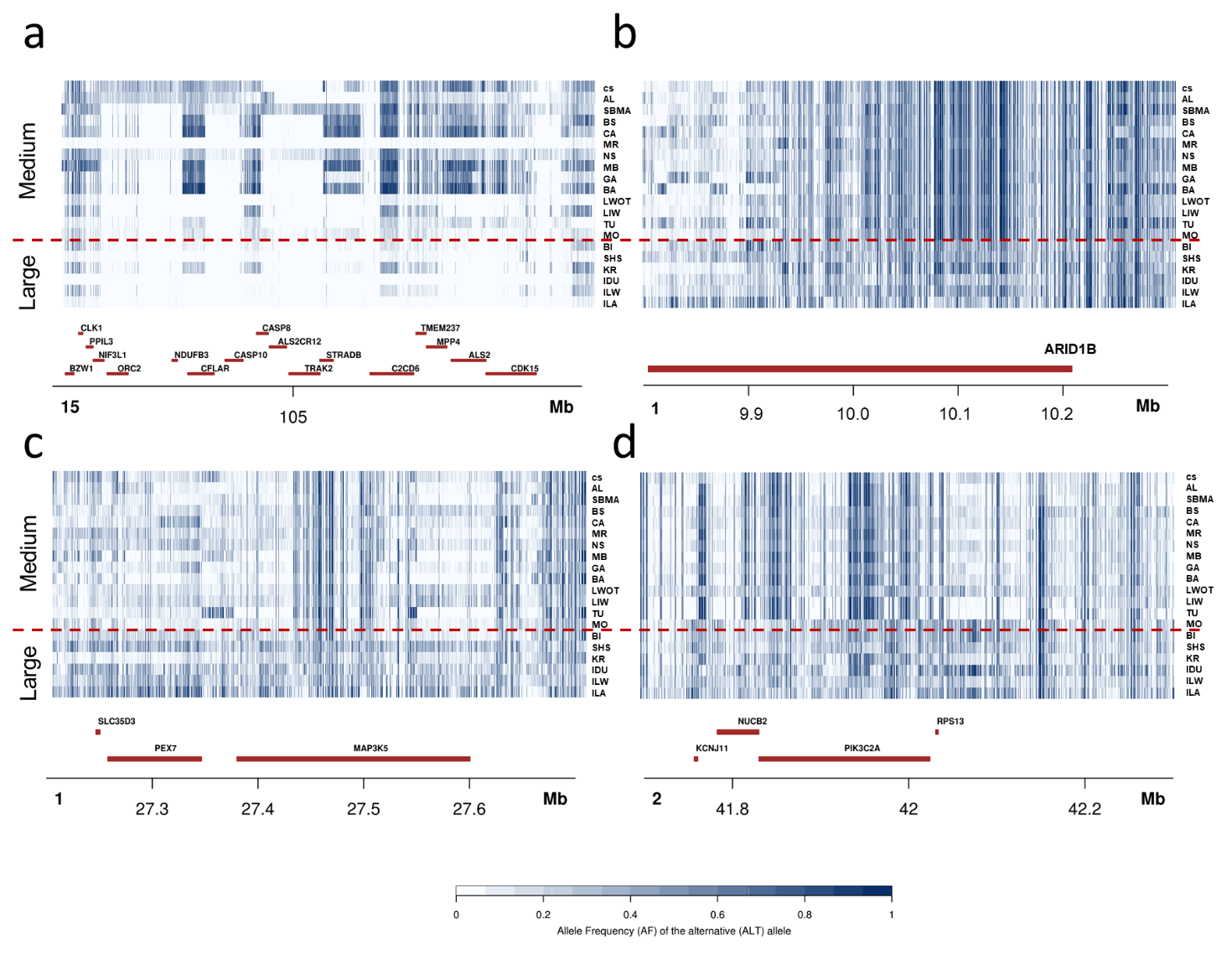

Supplement: Supplementary file 2 — Additional file 2: Figure S1. Evaluation of the D-statistics for the Kolmogorov–Smirnov test. Figure S2. Selection of the window size. (a) The number of windows with less than 10 SNPs over windows of variable size (in the range from 50 to 300-kb) is presented. Red dots represent windows larger than 100 kb, for which the number of windows with less than 10 SNPs started to asymptotically decrease. (b to d) Distribution of the number of SNPs contained in the 50-, 100- and 150-kb windows, respectively. Figure S3. FST based Neighbour-Joining tree. Next to the branches, the bootstrap test values expressed as percentage over 10,000 replicates are indicated in red. Figure S4. Mantel test between FST distance and the geographical distances (based on longitudinal and latitudinal coordinates) among autochthonous pig populations. Figure S5. Manhattan plots of the genome-wide HP analyses. Each dot represents a 100-kb genome window. Figure S6. Manhattan plots of the genome-wide FST analyses. Each dot represents a 100-kb genome window. Figure S7. Manhattan plots of the genome-wide FST analysis of breed groups Each dot represents a 100-kb genome window. Figure S8. Allele frequencies of SNPs in putative regions of signatures of selection detected in the FST analysis of middle vs large-sized pig breeds. Major signals were detected on: (a) SSC15 that carries the CASP10 gene, (b) SSC1 that carries the ARID1B gene, (c) SSC1 that carries the MAP3K5 gene (and the nearby PEX7) and (d) SSC2 that carries the PIK3C2A gene. [file 12711_2020_553_MOESM2_ESM.docx]
